# Estimation of Underlying Normal Distribution Parameters From Dichotomized Data

**DOI:** 10.1002/bimj.70173

**Published:** 2026-09-15

**Authors:** Zhaoze Liu, Longwen Shang, Mary Lesperance, Shuiqing Zhou, Xuekui Zhang

**Affiliations:** ^1^ Department of Mathematics and Statistics University of Victoria British Columbia Canada; ^2^ Innovation Research Institute of Zhejiang University of Technology Shengzhou China

**Keywords:** Bayesian MCMC, dichotomized data, GLM, GLMM, hierarchical model, MLE

## Abstract

Accurately estimating the parameters of a continuous distribution from dichotomized or aggregated data is a common problem in biomedical and environmental research. Many studies report only the proportion of subjects exceeding a threshold, without releasing individual‐level measurements. To address this limitation, we develop a hierarchical binomial‐probit modeling framework to reconstruct the parameters of an underlying normal distribution from threshold‐based data. The framework considers two principal settings. In the *fixed‐mean model*, all studies are assumed to share a common mean and variance, and the parameters μ and σ are estimated using a maximum‐likelihood estimator (MLE) and a generalized linear model (GLM) approximation. In the *random‐mean model*, each study has its own mean $\mu_{i}$ drawn from a population distribution $N(\mu_{0},\tau^{2})$ with a common within‐study variance $\sigma^{2}$; parameters $\mu_{0}$, σ, and τ are estimated using MLE, a generalized linear mixed model (GLMM) approximation, and a fully Bayesian Markov chain Monte Carlo (MCMC) method. Extensive simulations varying the number of studies, sample size, and heterogeneity ratio $r=\tau^{2}/\left(\sigma^{2}+\tau^{2}\right)$ evaluate estimator bias, variance, mean squared error, and coverage probability. Results show that MLE performs efficiently under well‐identified conditions, whereas GLMM and Bayesian estimators are more robust with small samples or strong heterogeneity. The proposed framework provides a unified and practical approach for inferring latent distributions from aggregated or privacy‐restricted data, with applications in clinical trial design, biomarker analysis, environmental monitoring, and quality control.

**Abbreviations**
GLM: generalized linear model
GLMM: generalized linear mixed model
MLE: maximum likelihood estimation
MCMC: Markov chain Monte Carlo

## 1 Introduction

Accurate estimation of a random variable's distribution from limited or aggregate data is a common challenge in life‐science studies, particularly clinical and biomedical research. Often, multiple studies may publish only the proportion of subjects that exceed certain thresholds, along with the total sample size, but not the full underlying continuous measurements. Such *thresholded* or *dichotomized* Summaries are pervasive in meta‐analyses of rare events or biomarkers (DerSimonian and Laird 1986; Jackson et al. 2018; Whitehead 2002), where continuous data cannot be shared or are simply unavailable. In clinical contexts, knowledge of the full distribution is particularly important for designing new trials, assessing population variability, and conducting power calculations (Deaton and Cartwright 2018). When only dichotomized data are available, the inability to recover latent parameters can obscure treatment effects, distort risk assessments, and limit reproducibility across trials.

This paper addresses the need to reconstruct the parameters of an underlying normal distribution—or a hierarchical normal model—using only such threshold‐based, dichotomized data. Although our initial motivation arises from the analysis of biomarker studies in oncology, the methodology is broadly applicable to any setting where continuous measurements are replaced by binomial summaries. Many clinical investigations report the fraction of patients whose biomarker levels, physiological values, or treatment responses exceed a predefined cutoff, without releasing the original measurements (Brahmer et al. 2012; Herbst et al. 2014). For such studies, direct access to the mean and variance of the latent distribution is impossible, yet these quantities are critical for designing follow‐up studies, assessing between‐trial heterogeneity, and evaluating treatment efficacy across different diagnostic thresholds. The proposed framework provides a rigorous statistical foundation for recovering these latent parameters, thereby enabling meta‐analytic synthesis and population‐level simulation even when only binary summaries are reported.

Beyond oncology, thresholded proportions arise in many other fields, such as: (1) Environmental science, where only fractions of pollutant concentrations exceeding a regulatory limit are published (Gibbons and Coleman 2001); (2) Reliability engineering, where the number of components surpassing a stress or lifetime limit is observed (Meeker et al. 2021); and (3) Quality control, where industrial processes are often summarized by the proportion of products failing a specification limit (Montgomery 2020). These applications share a common inferential challenge: how to estimate the parameters of the underlying distribution from aggregated binary data. In all these domains, the ability to reconstruct latent continuous distributions from thresholded information can guide policy evaluation, optimize process control, and improve decision‐making under uncertainty.

Classical approaches to meta‐analysis typically require either individual‐level data or continuous summary statistics (e.g., means and standard deviations). When only binary cut point‐based proportions are available, one might attempt naive logistic or probit regressions, but special care is needed to connect the cut point with the latent continuous scale (Christensen 2019; Cox 1972). Also, the standard random‐effects Meta‐analytic methods (DerSimonian and Laird 1986; Whitehead 2002) address heterogeneity in event proportions but do not directly reconstruct a continuous distribution under threshold‐based reporting. These traditional approaches cannot properly capture the link between the binomially observed proportions and the latent mean and variance that define the true population distribution. As a result, estimates derived from naive analyses can be biased or inefficient, particularly when study‐specific thresholds differ or sample sizes are small.

Here, we describe procedures—both maximum likelihood and regression‐based approaches—that properly account for the binomial nature of the data and the hierarchical structure when a random mean is involved. The proposed hierarchical binomial‐probit framework models each study's observed proportion as a binomial realization of the probability that a normally distributed variable falls below its cutoff. This structure allows estimation of the global mean, within‐study variance, and between‐study variance heterogeneity under a unified probabilistic model. It offers particular value in clinical trial design, where investigators often seek to integrate results from heterogeneous studies that report only binary response rates. By recovering the latent distribution of biomarker levels or treatment effects, the proposed method can improve population modeling, sample‐size planning, and cross‐trial comparisons, thereby enhancing evidence‐based decision‐making in translational medicine. Beyond clinical research, the same inferential strategy can be used in environmental regulation, toxicology, and manufacturing, where only aggregated compliance or exceedance rates are available. Overall, the method provides a rigorous, flexible, and broadly applicable solution to one of the most pervasive statistical problems across applied sciences: inferring the shape and variability of a hidden continuous distribution from limited, dichotomized data. In what follows, we formalize the problem, discuss three scenarios of increasing complexity (fixed mean, random mean with one threshold per study, and random mean with multiple thresholds per study), and outline the estimation procedures to address each setting.

The methodological contribution lies in the statistical treatment of threshold‐based data: (i) a unified likelihood‐based treatment of fixed‐ and random‐mean threshold models, (ii) a careful characterization of identifiability under dichotomization, and (iii) a systematic comparison of exact, approximate, and Bayesian inferential strategies under realistic reporting constraints.

## 2 Methods

### 2.1 Problem Setup

We consider a Gaussian random variable X measured across multiple studies indexed by i∈{1,…,I}. Each study uses a threshold or cutoff point $c_{i}$ to classify observations into “above” versus “below.” Let $p_{i}$ denote the *true* probability that X exceeds $c_{i}$ in each study i, that is:

(1) $$\begin{array}{c} p_{i}\;=\;P\left(X>c_{i}\right). \end{array}$$

We aim to estimate parameters of random variable X’s underlying normal distribution(s) based on observed data. For each ith study, we know threshold $c_{i}$, total sample size $n_{i}$, and $k_{i}$, which is the number of samples above the threshold. The observed probability that X exceeds the threshold in each study can be estimated as

(2) $$\begin{array}{c} p_{i}^{\mathrm{obs}}\;=\;k_{i}/n_{i}, \end{array}$$

i. *Scenario 1: Fixed mean*. Assume all I studies share the same distribution
(3) $$\begin{array}{c} X∼N(\mu ,\;\sigma^{2}). \end{array}$$
  Using observed data ${\left\{\left(n_{i},c_{i},p_{i}^{\mathrm{obs}}\right)\right\}}_{i=1}^{I}$ or equivalently ${\left\{\left(n_{i},c_{i},k_{i}\right)\right\}}_{i=1}^{I}$, we aim to estimate (μ,σ).
ii. *Scenario 2: Random mean*. Assume all settings are the same as in Scenario 1, except that the mean varies across studies and follows an unknown normal distribution, that is,
(4) $$\begin{array}{c} X_{i}\;∼\;N\left(\mu_{i},\;\sigma^{2}\right),\;\mu_{i}\;∼\;N\left(\mu_{0},\;\tau^{2}\right). \end{array}$$
  However, we will show that, given these data, one can only estimate the parameters ($\mu_{0},\sigma^{2}+\tau^{2}$)—that is, the overall mean and total variance, but not $\sigma^{2}$ or $\tau^{2}$ separately. We will propose two approaches to address this unidentifiable issue: (1) reporting the total variance $\sigma^{2}+\tau^{2}$, which is sufficient for many applications, or (2) collecting more data when possible to estimate all parameters, which leads to Scenario 3 below.
iii. *Scenario 3: Multiple thresholds per study*. If a study i reports multiple thresholds or cutoff points $\left\{c_{ij}\right\}$ with proportions $\left\{p_{ij}^{\mathrm{obs}}\right\}$, which means the observed probability X exceeding threshold $c_{ij-1}$ and below $c_{ij}$ is $p_{ij}^{\mathrm{obs}}$, one can break the degeneracy and estimate σ versus τ separately, because each study has enough within‐study information about the shape of the distribution.

### 2.2 Scenario 1: Fixed Mean and Variance

Let each study i report $\left\{n_{i},c_{i},\;p_{i}^{\mathrm{obs}},k_{i}\right\}$. The *true* probability is

(5) $$\begin{array}{c} p_{i}\;=\;P\left(X>c_{i}\right)\;=\;1-\Phi \;\left(\frac{c_{i}-\mu }{\sigma }\right). \end{array}$$

To estimate the parameters (μ,σ) from the data and model described above, we propose two approaches below, including maximum likelihood estimation (MLE) and generalized linear model (GLM) probit.

#### 2.2.1 MLE via Binomial Likelihood Using Weighted Ordinary Least Squares (WOLS) as Initial Value

Rearranging (5), we get

(6) $$\begin{array}{c} \Phi^{-1}\left(1-p_{i}\right)=c_{i}/\sigma -\mu /\sigma , \end{array}$$

which can be approximated by

(7) $$\begin{array}{c} c_{i}\;\approx \;\mu +\sigma \;z_{i}, \end{array}$$

where $z_{i}=\Phi^{-1}\;\left(1-p_{i}^{\mathrm{obs}}\right)$ approximates $\Phi^{-1}\left(1-p_{i}\right)$ by replacing the unknown true probability $p_{i}$ with the observed proportion $p_{i}^{\mathrm{obs}}\;=\;k_{i}/n_{i}$. We treat $\left(z_{i},\;c_{i}\right)$ as data in a linear model with coefficients (μ,σ) and weights $n_{i}$. The estimates of μ and σ can be obtained by solving a WOLS problem:

(8) $$\begin{array}{c} \left(\hat{\mu },\hat{\sigma }\right)=\min_{\mu ,\sigma }\sum_{i=1}^{I}n_{i}\;{\left(c_{i}-\mu -\sigma \;z_{i}\right)}^{2}. \end{array}$$

This linearized WOLS formulation can be interpreted as a first‐order delta‐method expansion of the probit likelihood and is asymptotically equivalent to the maximum likelihood estimator as $n_{i}\to \infty$. Since the WOLS estimator arises from a first‐order delta‐method linearization of the binomial‐probit likelihood. For study i, let $k_{i}∼\mathrm{Binomial}\left(n_{i},p_{i}\right)$ and ${\hat{p}}_{i}=k_{i}/n_{i}$. Under the probit model $p_{i}=1-\Phi \;\left(\frac{c_{i}-\mu }{\sigma }\right)$, we have $\Phi^{-1}\left(1-p_{i}\right)=\frac{c_{i}-\mu }{\sigma }$. By the central limit theorem, ${\hat{p}}_{i}=p_{i}+O_{p}\left(n_{i}^{-1/2}\right)$, and applying a first‐order Taylor expansion to $z_{i}=\Phi^{-1}\left(1-{\hat{p}}_{i}\right)$ yields $z_{i}=\frac{c_{i}-\mu }{\sigma }+O_{p}\left(n_{i}^{-1/2}\right)$, so that $c_{i}=\mu +\sigma z_{i}+O_{p}\left(n_{i}^{-1/2}\right)$. Moreover, $\mathrm{Var}\left(z_{i}\right)=O\left(n_{i}^{-1}\right)$, implying that the efficient weighting is proportional to $n_{i}$. Hence, the WOLS objective $\sum_{i}n_{i}{\left(c_{i}-\mu -\sigma z_{i}\right)}^{2}$ corresponds to the quadratic (second‐order) expansion of the binomial log‐likelihood. As $n_{i}\to \infty$ and the true probabilities satisfy $\varepsilon <p_{i}<1-\varepsilon$, the likelihood becomes asymptotically quadratic, and the higher‐order remainder terms vanish, yielding ${\hat{\theta }}_{\mathrm{WOLS}}-{\hat{\theta }}_{\mathrm{MLE}}\overset{p}{\to }0$, so that the two estimators are asymptotically equivalent and share the same limiting normal distribution.

For each study i, $k_{i}$ is number of sample above the threshold with probability $p_{i}$, so $k_{i}∼\mathrm{Binomial}\left(n_{i},\;p_{i}\right)$, so the likelihood is

(9) L(μ,σ)=∏i=1Iniki1−Φci−μσkiΦci−μσni−ki.

Since this likelihood inside contains a normal distribution, which is too complex for a closed‐form solution, we use numerical optimization. For the software implementation, we may employ the numerical solver optim() in R, with the Broyden–Fletcher–Goldfarb–Shanno (BFGS) method being a common choice for smooth likelihood functions. While MLE fully respects the distributional assumptions and yields statistically efficient estimates, its numerical optimization can be sensitive to starting values since it fully respects the binomial nature of the data. When $n_{i}$ is large and $p_{i}^{\mathrm{obs}}$ is not too close to 0 or 1 (σ may close to 0), $p_{i}^{\mathrm{obs}}$ approximates $p_{i}$ well; in this case, the difference from MLE is minor. In such situations, the weighted OLS approach is computationally more efficient and can also provide good initial values for the MLE optimization, potentially speeding up convergence.

#### 2.2.2 GLM Probit Regression Model

Assume a latent normal variable $X∼N(\mu ,\;\sigma^{2})$, and for each study i we check whether each draw exceeds the threshold $c_{i}$ and for each threshold $c_{i}$, define the binary response variable:

(10) $$\begin{array}{c} Y_{ij}=\left\{\begin{array}{cc} 1 & \text{if}\;X_{ij}>c_{i},\\ 0 & \text{if}\;X_{ij}\leq c_{i}, \end{array}\right.\;j=1,\cdots ,n_{i}. \end{array}$$

The probability of success (exceedance) is

(11) $$\begin{array}{c} \mathrm{Pr}\left(Y_{ij}=1|c_{i}\right)=\mathrm{Pr}\left(X>c_{i}\right). \end{array}$$

To rearrange in regression form, we can rewrite this probability as

(12) $$\begin{array}{c} \mathrm{Pr}\left(Y_{ij}=1|c_{i}\right)=\Phi \;\left(\frac{\mu }{\sigma }-\frac{c_{i}}{\sigma }\right). \end{array}$$

Define the transformed parameters

(13) $$\begin{array}{c} \alpha =\frac{\mu }{\sigma },\;\beta =\frac{1}{\sigma }. \end{array}$$

Then

(14) $$\begin{array}{c} \mathrm{Pr}\left(Y_{ij}=1|c_{i}\right)=\Phi \;\left(\alpha +\beta (-c_{i})\right). \end{array}$$

If we define the predictor variable as

(15) $$\begin{array}{c} x_{i}=-c_{i}. \end{array}$$

Then the model becomes

(16) $$\begin{array}{c} \mathrm{Pr}\left(Y_{ij}=1|x_{i}\right)=\Phi \left(\alpha +\beta x_{i}\right). \end{array}$$

This is exactly the form of a probit regression model:

- Binary response: $Y_{ij}\in \left\{0,1\right\}$,
- Linear predictor: $\eta_{i}=\alpha +\beta x_{i}$,
- Link function: probit, that is,
$$g\left(p_{i}\right)=\Phi^{-1}\left(p_{i}\right)=\alpha +\beta x_{i}.$$

After fitting the probit regression and obtaining estimates $\hat{\alpha },\hat{\beta }$, We can recover the original parameters as

(17) $$\begin{array}{c} \hat{\mu }=\frac{\hat{\alpha }}{\hat{\beta }},\;\hat{\sigma }=\frac{1}{\hat{\beta }}. \end{array}$$

The probit regression formulation provides a natural and computationally efficient way to estimate μ and σ. Rather than implementing custom optimization of the binomial likelihood (as above), the problem can be expressed as a GLM with a probit link, where the binary outcome indicates whether an observation exceeds the threshold and the predictor is the negative threshold value. This reformulation allows the estimation to be carried out directly using standard GLM routines in R and stats (R Core Team 2025) package's glm() function with family = binomial(link = ”probit”), which automatically handles iterative fitting, variance estimation, and inference. By mapping the regression coefficients back to (μ, σ), we obtain parameter estimates consistent with the latent normal threshold model while benefiting from the stability, diagnostics, and flexibility of widely used GLM software. This approach is particularly advantageous when working with multiple thresholds and moderate to large sample sizes, as it avoids the need to explicitly code the likelihood and its derivatives while still yielding estimates that respect the exact binomial‐probit structure of the data.

### 2.3 Scenario 2: Mean Varies Across Studies

The setup is identical to Scenario 1, except that the study‐specific means vary according to

(18) $$\begin{array}{c} X_{i}∼N\left(\mu_{i},\;\sigma^{2}\right),\;\mu_{i}∼N\left(\mu_{0},\;\tau^{2}\right). \end{array}$$

Each study reports a single threshold $c_{i}$, total sample size $n_{i}$, and the number of observations exceeding the threshold, $k_{i}$. For study i, the observed exceedance count satisfies

(19) $$\begin{array}{c} k_{i}|n_{i}∼\mathrm{Binomial}\left(n_{i},\;p_{i}\right), \end{array}$$

where the success probability is

(20) $$\begin{array}{c} p_{i}\;=\;\mathrm{Pr}\left(X_{i}>c_{i}\right)\;=\;E_{\mu_{i}}\;\left[1-\Phi \;\left(\frac{c_{i}-\mu_{i}}{\sigma }\right)\right]. \end{array}$$

Using the normal–normal hierarchical structure, this expectation has the closed‐form expression

(21) $$\begin{array}{c} p_{i}\;=\;1-\Phi \;\left(\frac{c_{i}-\mu_{0}}{\sqrt{\sigma^{2}+\tau^{2}}}\right). \end{array}$$

Equation (21) characterizes the sampling distribution of the observed data $\left(k_{i},n_{i},c_{i}\right)$. In particular, the binomial likelihood for $k_{i}$ depends on the variance components $\left(\sigma^{2},\tau^{2}\right)$ only through their sum

$$S^{2}=\sigma^{2}+\tau^{2}.$$

As a result, from single‐threshold data, the parameters $\sigma^{2}$ and $\tau^{2}$ are not separately identifiable: any pair $\left(\sigma^{2},\tau^{2}\right)$ yielding the same $S^{2}$ induces the same distribution for the observed exceedance counts. Consequently, only the population mean $\mu_{0}$ and the total variance $S^{2}$ can be identified under this design.

In practice, one may therefore either (i) report the combined scale parameter $S=\sqrt{\sigma^{2}+\tau^{2}}$, which is sufficient for many applications, or (ii) collect multiple thresholds per study to separately identify $\sigma^{2}$ and $\tau^{2}$, as discussed in Scenario 3 below.

### 2.4 Scenario 3: Multiple Thresholds per Study

To separate the within‐study variance $\sigma^{2}$ and between‐study variance $\tau^{2}$ from the total variance $\sigma^{2}+\tau^{2}$, one can collect more data from each study. Assume each study i provides $m_{i}$ cutoff data points and sample size $n_{i}$ ${\left\{(n_{i},c_{ij})\right\}}_{j=1}^{m_{i}}$ and ${\left\{(k_{ij},\pi_{ij})\right\}}_{j=1}^{m_{i}+1}$, where $(k_{i1},\text{…},k_{i,m_{i}+1}$) are observed counts in the $m_{i}+1$ intervals $k_{ij}|\mu_{i},\sigma \;∼\;\mathrm{Multinomial}\;\left(n_{i},\pi_{ij}\left(\mu_{i},\sigma \right)\right)$, and $\pi_{ij}$ is relevant probability that points in each interval. We adopt the random‐mean model from Scenario 2:

(22) $$\begin{array}{c} \mu_{i}∼N\left(\mu_{0},\;\tau^{2}\right),\;X_{i}∼N\left(\mu_{i},\;\sigma^{2}\right), \end{array}$$

so that the *true* probability in each interval defined by

(23) $$\pi_{ij}=\left\{\begin{array}{cc} 1-\Phi \left(\frac{c_{i1}-\mu_{i}}{\sigma }\right), & j=1\\ \Phi \left(\frac{c_{i,j-1}-\mu_{i}}{\sigma }\right)-\Phi \left(\frac{c_{ij}-\mu_{i}}{\sigma }\right), & 2\leq j\leq m_{i}\\ \Phi \left(\frac{c_{i,m_{i}}-\mu_{i}}{\sigma }\right), & j=m_{i}+1. \end{array}\right.$$

Since only the interval counts are observed, we work with the empirical proportions $\pi_{ij}^{\mathrm{obs}}=k_{ij}/n_{i}$ as estimates of the latent interval probabilities $\pi_{ij}\left(\mu_{i},\sigma \right)$ implied by the probit‐Normal model. Multiple thresholds within each study provide information on the within‐study variance $\sigma^{2}$, while variation across studies informs the between‐study variance $\tau^{2}$, allowing the full parameter vector $\left(\mu_{0},\sigma ,\tau \right)$ to be identified. We outline three inferential approaches for this setting below: maximum likelihood estimation via numerical integration, full Bayesian inference using Markov chain Monte Carlo (MCMC), and a generalized linear mixed model (GLMM) approximation.

#### 2.4.1 MLE, Integrate Out $\left\{\mu_{i}\right\}$

For study i=1,…,I, let ordered thresholds $c_{i0}<c_{i1}<\cdots <c_{im_{i}}$ define $m_{i}+1$ intervals with $c_{i0}=-\infty$ and $c_{i,m_{i}+1}=+\infty$. We assume the latent probit‐Normal model

$$Y_{ij}^{*}=\mu_{i}+\sigma Z_{ij},\;Z_{ij}∼N\left(0,1\right),\;\mu_{i}∼N\left(\mu_{0},\tau^{2}\right).$$

Conditional on $\mu_{i}$, the probability of falling in interval j is

(24) $$\pi_{ij}\left(\mu_{i},\sigma \right)=\Phi \;\left(\frac{c_{ij}-\mu_{i}}{\sigma }\right)-\Phi \;\left(\frac{c_{i,j-1}-\mu_{i}}{\sigma }\right),$$

and the multinomial likelihood for counts $k_{i\cdot }=\left(k_{i1},\text{…},k_{i,m_{i}+1}\right)$ with total $n_{i}=\sum_{j}k_{ij}$ is

(25) $$L_{i}\left(k_{i\cdot }|\mu_{i},\sigma \right)=\frac{n_{i}!}{\prod_{j=1}^{m_{i}+1}k_{ij}!}\prod_{j=1}^{m_{i}+1}\pi_{ij}{\left(\mu_{i},\sigma \right)}^{k_{ij}}.$$

Integrating over the random effect $\mu_{i}∼N\left(\mu_{0},\tau^{2}\right)$ yields the marginal

(26) $$\begin{array}{c} L_{i}\left(\mu_{0},\sigma ,\tau \right)=\int_{-\infty }^{\infty }L_{i}\left(k_{i\cdot }|\mu_{i},\sigma \right)\;\frac{1}{\sqrt{2\pi }\tau }\exp\;\left[-\frac{{\left(\mu_{i}-\mu_{0}\right)}^{2}}{2\tau^{2}}\right]\;d\mu_{i}, \end{array}$$

and $L\left(\mu_{0},\sigma ,\tau \right)=\prod_{i}L_{i}\left(\mu_{0},\sigma ,\tau \right)$.

Let U,Z∼N(0,1) be independent and write $\mu_{i}=\mu_{0}+\tau U$. For any cut point c,

$$\begin{array}{c} \begin{array}{cc} E_{\mu_{i}}\;\left[\Phi \;\left(\frac{\mu_{i}-c}{\sigma }\right)\right] & =\mathrm{Pr}(\mu_{0}+\tau U+\sigma Z>c)\\ & =\mathrm{Pr}\;\left(N(\mu_{0},\tau^{2}+\sigma^{2})>c\right)=\Phi \;\left(\frac{\mu_{0}-c}{S}\right),\\ & \;S=\sqrt{\sigma^{2}+\tau^{2}}. \end{array} \end{array}$$

Thus, the *marginal tail probability* depends on (σ,τ) only through the combined scale S. For a *single* threshold ($m_{i}=1$), this identity is exact for the integrated likelihood; with *multiple* thresholds ($m_{i}>1$) it provides a useful approximation that correctly indicates why the likelihood is nearly flat along contours $S=\sqrt{\sigma^{2}+\tau^{2}}$, making σ and τ hard to separate.

For multiple thresholds, the product in (25) prevents factorization of (26), so we evaluate the one‐dimensional integral numerically. With the change of variable

$$t=\frac{\mu_{i}-\mu_{0}}{\sqrt{2}\;\tau }\;⟹\;\mu_{i}=\mu_{0}+\sqrt{2}\;\tau \;t,\;d\mu_{i}=\sqrt{2}\;\tau \;dt,$$

(26) becomes

(27) $$\begin{array}{cc} L_{i}\left(\mu_{0},\sigma ,\tau \right) & =\frac{1}{\sqrt{\pi }}\int_{-\infty }^{\infty }e^{-t^{2}}\;\left[\frac{n_{i}!}{\prod_{j}k_{ij}!}\prod_{j=1}^{m_{i}+1}\pi_{ij}{\left(\mu_{0}+\sqrt{2}\tau t,\sigma \right)}^{k_{ij}}\right]dt. \end{array}$$

Applying a G‐point Gauss–Hermite (GH) rule, $\int e^{-t^{2}}f\left(t\right)\;dt\approx \sum_{g=1}^{G}w_{g}f\left(x_{g}\right)$, gives

(28) $$\begin{array}{c} L_{i}\left(\mu_{0},\sigma ,\tau \right)\approx \frac{1}{\sqrt{\pi }}\sum_{g=1}^{G}w_{g}\left[\frac{n_{i}!}{\prod_{j}k_{ij}!}\prod_{j=1}^{m_{i}+1}\pi_{ij}{\left(\mu_{0}+\sqrt{2}\tau x_{g},\sigma \right)}^{k_{ij}}\right], \end{array}$$

and the approximate log‐likelihood

(29) $$\begin{array}{cc} & \log L\left(\mu_{0},\sigma ,\tau \right)\\ & \approx \sum_{i=1}^{I}\log\left[\frac{1}{\sqrt{\pi }}\sum_{g=1}^{G}w_{g}\cdot \left(\frac{n_{i}!}{\prod_{j}k_{ij}!}\prod_{j=1}^{m_{i}+1}\pi_{ij}{\left(\mu_{0}+\sqrt{2}\tau x_{g},\sigma \right)}^{k_{ij}}\right)\right]. \end{array}$$

Here, “maximum likelihood” refers to maximization of the GH‐approximated marginal likelihood defined by Equation (28) and its corresponding summed log‐likelihood across studies (29). The exact marginal likelihood in Equation (26) is analytically intractable when multiple thresholds are present. Closed‐form expressions involving $S=\sqrt{\sigma^{2}+\tau^{2}}$ are used only to explain the near‐ridge structure of the likelihood and to guide initialization, not for likelihood evaluation. We maximize this numerically (e.g., optim in R) to obtain the MLE $\left({\hat{\mu }}_{0},\hat{\sigma },\hat{\tau }\right)$. Empirically, the GH‐based likelihood exhibits the same ridge structure anticipated by the S‐based argument above, confirming that σ and τ are weakly identified while S is well determined.

Alongside the fixed‐node GH approximation, we evaluated the same marginal likelihood using mode‐centered adaptive integration (MCAI) as a numerical sensitivity analysis. This calculation evaluates the same marginal likelihood in (26) under the same hierarchical model and does not define a new statistical estimator or modify the inferential target. Mode and curvature‐based adaptation are established ideas in adaptive quadrature for mixed models (Liu and Pierce 1994; Pinheiro and Bates 1995). For each study‐specific marginal integral, we first locate the mode of the integrand, which identifies the region contributing most to the integral. We then use the local curvature at that mode to define a study‐specific integration interval: sharply concentrated integrands receive a narrower interval, whereas flatter integrands receive a wider interval. Finally, the integrand is rescaled by its value at the mode before numerical integration to improve numerical stability. The parameter K controls the numerical half‐width of the mode‐centered integration domain, $\left[x_{i}^{*}-Ks_{i},\;x_{i}^{*}+Ks_{i}\right]$, where $x_{i}^{*}$ is the mode of the study‐specific integrand and $s_{i}$ is its local curvature scale. Thus, K specifies the number of local peak‐widths retained on each side of the dominant integration region. It is not a parameter of the hierarchical model, does not alter the likelihood target, and does not represent a scientific or statistical tuning parameter. Increasing K retains more of the integrand tails and can reduce numerical truncation error, although at additional computational cost. We used K=10 as the primary MCAI setting and evaluated K∈{4,6,8,10,12} in a paired numerical sensitivity analysis. Full implementation details are provided in the Supporting Information Section S1.

To stabilize optimization, within each study i we form $z_{ij}=\Phi^{-1}\left(1-p_{ij}^{\mathrm{obs}}\right)$ and fit $\;c_{ij}\approx \mu_{i}+\sigma z_{ij}$ by (weighted) least squares, taking the study intercepts ${\hat{\mu }}_{i}$ and a pooled slope $\hat{\sigma }$. We set

$$\mu_{0}^{\mathrm{init}}=\frac{1}{I}\sum_{i=1}^{I}{\hat{\mu }}_{i},\;\tau^{\mathrm{init}}=\mathrm{sd}\left({\hat{\mu }}_{1},\text{…},{\hat{\mu }}_{I}\right),$$

optionally bias‐reduced using intercept standard errors when available. This initializer respects the common σ, uses all cut points, and is computationally cheap.

#### 2.4.2 Full Bayesian MCMC, Sampling $\left\{\mu_{i}\right\}$

Instead of integrating out each $\mu_{i}$, one can use a full Bayesian approach (Bernardo et al. 1994). Recent Bayesian studies have demonstrated the use of posterior inference, prior specification, and MCMC methods in survival, reliability, and lifetime‐data settings involving censored or incomplete observations (Albadawy et al. 2024; Almetwally 2026a;;2026b; Almetwally et al. 2025; Elgarhy et al. 2025; Hamdy and Almetwally 2023; Hassan and Abdelghaffar 2025; Kalantan and Salem 2025). Although these studies consider different outcome distributions, censoring mechanisms, and scientific applications from the present threshold‐based hierarchical probit model, they illustrate the broader utility of Bayesian computation for distributional and reliability inference. The interval probabilities $\pi_{ij}\left(\mu_{i},\sigma \right)$ are defined above, and the within‐study likelihood is multinomial:

(30) $$\begin{array}{c} k_{i}|\mu_{i},\sigma \;∼\;\mathrm{Multinomial}\;\left(n_{i},\pi_{i}\left(\mu_{i},\sigma \right)\right),\;\pi_{i}=\left(\pi_{i1},\cdots ,\pi_{i,m_{i}+1}\right). \end{array}$$

With priors $p\left(\mu_{0}\right),p\left(\sigma \right),p\left(\tau \right)$ and the Gaussian random effects on $\mu_{i}$, the joint posterior is

(31) $$\begin{array}{cc} & p(\mu_{0},\sigma ,\tau ,\mu_{1:I}|\left\{k_{i}\right\})\\ & \propto \prod_{i=1}^{I}\{\prod_{j=1}^{m_{i}+1}\pi_{ij}{\left(\mu_{i},\sigma \right)}^{k_{ij}}\}\cdot \phi \;\left(\frac{\mu_{i}-\mu_{0}}{\tau }\right)\cdot p\left(\mu_{0}\right)p\left(\sigma \right)p\left(\tau \right). \end{array}$$

For implementation, we use R Stan(Stan Development Team 2024), which employs Hamiltonian Monte Carlo to sample from the posterior. Recent work on adaptive Hamiltonian methods for uncertainty quantification in lifetime models further illustrates the usefulness of Hamiltonian‐based Bayesian computation when posterior evaluation is analytically intractable (Almetwally 2026b). A numerically stable implementation uses noncentered effects $\mu_{i}=\mu_{0}+\tau \mu_{i}^{\mathrm{raw}}$, with $\mu_{i}^{\mathrm{raw}}∼N\left(0,1\right)$. For each study i:

1. compute the $m_{i}\;+\;1$ interval probabilities.
2. increment the target with the multinomial log probability mass function (pmf) $\log p(k_{i}|\pi_{i})$.

(32) $$\begin{array}{c} \begin{array}{cc} y_{i1} & ∼\mathrm{Binomial}\;\left(n_{i},\;\pi_{i1}\right),\\ y_{i2} & ∼\mathrm{Binomial}\;\left(n_{i}-y_{i1},\;\frac{\pi_{i2}}{1-\pi_{i1}}\right),\\ & \;\vdots \\ y_{i,m_{i}} & ∼\mathrm{Binomial}\;\left(n_{i}-\sum_{j<m_{i}}y_{ij},\;\frac{\pi_{i,m_{i}}}{1-\sum_{j<m_{i}}\pi_{ij}}\right), \end{array} \end{array}$$

$y_{i,1},\text{…}y_{i,m_{i}}$

Because Stan does not support ragged arrays, pass the number of intervals $J_{i}=m_{i}+1$ for each study and loop only over the first $J_{i}$ entries of the p and k vectors. *Do not pad with artificial cut points*. Alternatively, use the exact “stick‐breaking” factorization (Broderick et al. 2012): are number of observations in each interval and $y_{i,m_{i}+1}=n_{i}-\sum_{j\leq m_{i}}y_{ij}$. The sum of these binomial log pmfs equals the multinomial log pmf up to a constant, and is often more stable than a single multinomial call. Then using weakly informative priors on scale parameters, for example, $\sigma ∼\mathrm{half}-\mathrm{Normal}(0,s_{\sigma })$ or half‐Cauchy, and similarly for τ; place a broad normal prior on $\mu_{0}$. Also work on the log‐scale for σ,τ to constrain extreme z values in Φ(z) for numerical safety.

However, the multinomial MCMC method in practice may have worse performance due to few reasons:

1. **Incorrect conversion from cumulative to interval probabilities**. When the reported quantities are right‐tail proportions $p_{ij}^{\left(\mathrm{right}\right)}=\mathrm{Pr}\left(X>c_{ij}\right)$, the corresponding interval probability vector must be constructed as
$$\left(1-p_{i1}^{\left(\mathrm{right}\right)},\;p_{i1}^{\left(\mathrm{right}\right)}-p_{i2}^{\left(\mathrm{right}\right)},\;\cdots ,\;p_{i,m_{i}-1}^{\left(\mathrm{right}\right)}-p_{i,m_{i}}^{\left(\mathrm{right}\right)},\;p_{i,m_{i}}^{\left(\mathrm{right}\right)}\right).$$
  Treating the right‐tail proportions as if they formed a cumulative distribution function increasing from 0 to 1 (e.g., by differencing from 0 upward) results in a misspecified likelihood. Such misspecification can lead to substantial distortion of posterior or likelihood‐based inference.
2. **Artificial padding of cut points**. Extending each study to a common number of cut points K by appending artificially large threshold values may induce severe numerical artifacts. In particular, when a padded cut point lies far in the upper tail, the implied interval probability is nearly 1 under the probit model, while the observed count in that interval is typically zero. This mismatch creates excessive likelihood penalties and can distort parameter estimates. Instead, each study should be modeled using its actual number of intervals $J_{i}$, without artificial augmentation.
3. **Replacing observed counts with simulated counts**. Generating artificial counts $k_{ij}∼\mathrm{Multinomial}\left(n_{i},{\hat{\pi }}_{ij}\right)$ from reported proportions introduces additional Monte Carlo variation directly into the likelihood evaluation. If the true sample sizes $n_{i}$ are unavailable, it is preferable to work with a soft‐count (cross‐entropy) objective or use deterministic integer allocations that preserve the reported proportions exactly, up to minimal rounding adjustment.
4. **Prior specification and parameter scaling**. Overly restrictive priors on scale parameters (e.g., truncated normal priors with small variance on σ or τ) can induce bias toward extreme variance decompositions. Weakly informative priors specified on the log‐scale (or half‐normal / half‐Cauchy priors with appropriate scale) generally provide more stable and robust inference.

Due to these reasons, we used a pragmatic alternative model when ignoring within‐study dependence. If only right‐tail proportions above each cut point are directly available in each study, each study can be considered as binomial and can be multiplied:

(33) $$\begin{array}{c} k_{ij}\;\overset{\text{as if}}{∼}\;\mathrm{Binomial}\;\left(n_{i},\;p_{ij}\left(\mu_{i},\sigma \right)\right),\;p_{ij}\left(\mu_{i},\sigma \right)=1-\Phi \;\left(\frac{c_{ij}-\mu_{i}}{\sigma }\right), \end{array}$$

together with the same Gaussian random effects on $\mu_{i}$ and priors on $\left(\mu_{0},\sigma ,\tau \right)$. Because the $k_{ij}$ built from the same $n_{i}$ are not independent, this defines a composite (pseudo‐) likelihood. An HMC implementation targets the pseudo‐posterior $\;p(\mu_{0},\sigma ,\tau ,\left\{\mu_{i}\right\}|\text{data})$ which proportional to

(34) $$\begin{array}{ccc} & & \prod_{i=1}^{I}\left[\prod_{j=1}^{m_{i}}\mathrm{Binom}\left(k_{ij}|n_{i},\;1-\Phi \left(\frac{c_{ij}-\mu_{i}}{\sigma }\right)\right)\;\times \;N\;\left(\mu_{i}|\mu_{0},\tau^{2}\right)\right]\\ & & \;\times \;\pi (\mu_{0},\sigma ,\tau ). \end{array}$$

For prior, we specified $\mu_{0}∼N\left(0,10^{2}\right)$, $\sigma ∼{\mathrm{Cauchy}}^{+}\left(0,5\right)$, $\tau ∼{\mathrm{Cauchy}}^{+}\left(0,5\right)$. Here, ${\mathrm{Cauchy}}^{+}\left(0,5\right)$ denotes a half‐Cauchy distribution induced by constraining σ and τ to be positive. These priors were selected as generic weakly informative priors rather than as application‐specific elicited distributions. The normal prior on $\mu_{0}$ provides broad support over the location range considered in the simulation study. The half‐Cauchy priors enforce positivity of the two scale parameters while retaining heavy tails that allow values substantially larger than the values considered in the simulation grid. Thus, the prior specification provides mild regularization and a proper posterior without strongly favoring a particular decomposition of total variation into $\sigma^{2}$ and $\tau^{2}$. These priors do not remove the fundamental weak‐identification issue for the separate variance components. Rather, they regularize extreme regions of the parameter space during posterior sampling.

For moderate numbers of cuts and nonextreme probabilities, the composite target has nearly the same mode as the exact multinomial target, so point estimates for $\left(\mu_{0},\sigma ,\tau \right)$ are close; gradients are simple and HMC mixes well. Moreover, if the data truly observed are cumulative right‐tail proportions at each cut (not interval counts), this posterior matches the reported summaries directly. However, information within a study is double‐counted, the composite posterior is too concentrated; credible intervals are anticonservative. To report uncertainty: either fit the exact multinomial model, or calibrate by (i) a study‐level bootstrap (resampling studies) or (ii) sensitivity over an effective study size when only proportions are available.

If only interval proportions ${\hat{\pi }}_{ij}$ are known (and perhaps $n_{i}$ are unknown), one can replace counts by “soft counts” and maximize (or sample from) the cross‐entropy objective:

$$\ell_{i}\left(\mu_{i},\sigma \right)\;=\;N_{\;\mathrm{eff}}\sum_{j=1}^{m_{i}+1}{\hat{\pi }}_{ij}\;\log\pi_{ij}\left(\mu_{i},\sigma \right),$$

with $N_{\;\mathrm{eff}}=n_{i}$ when available, or a common constant otherwise. This yields the same point estimates as using integer counts up to rounding, while avoiding artificial simulation noise, uncertainty scales with $N_{\;\mathrm{eff}}$, so sensitivity analysis or bootstrapping is recommended when $n_{i}$ are unknown.

Because the reported summaries are right‐tail proportions at multiple cut points, treating them as independent binomial margins ignores within‐study dependence and defines a composite likelihood. Algorithm 1 gives a conceptual blockwise Metropolis‐within‐Gibbs representation of the composite pseudo‐posterior in Equation (34). In the actual implementation, however, we use Stan's Hamiltonian Monte Carlo sampler rather than a manually coded Metropolis‐within‐Gibbs algorithm. All reported Bayesian analyses were implemented in Stan using Hamiltonian Monte Carlo under the noncentered parameterization described above.

ALGORITHM 1 **Conceptual blockwise Metropolis‐within‐Gibbs sampler**

**Table:** 

**Require:** Threshold summary data $\left\{k_{ij},n_{i},c_{ij}:i=1,\text{…},I;\;j=1,\text{…},m_{i}\right\}$; initial values $\mu_{0}^{\left(0\right)},\sigma^{\left(0\right)},\tau^{\left(0\right)},{\left\{\mu_{i}^{\left(0\right)}\right\}}_{i=1}^{I}$; priors $\pi \left(\mu_{0}\right),\pi \left(\sigma \right),\pi \left(\tau \right)$; number of MCMC iterations M.	
**Ensure:** Samples from the composite pseudo‐posterior of $\mu_{0},\sigma ,\tau$, and ${\left\{\mu_{i}\right\}}_{i=1}^{I}$.	
1:	Initialize $\mu_{0}^{\left(0\right)},\sigma^{\left(0\right)},\tau^{\left(0\right)},{\left\{\mu_{i}^{\left(0\right)}\right\}}_{i=1}^{I}$.
2:	**for** m=1,…,M **do**
3:	**for** i=1,…,I **do**
4:	Update the study‐specific mean $\mu_{i}^{\left(m\right)}$ using a Metropolis step with target $$p\left(\mu_{i}|\cdot \right)\propto \prod_{j=1}^{m_{i}}\mathrm{Binom}\left(k_{ij}|n_{i},\;1-\Phi \left(\frac{c_{ij}-\mu_{i}}{\sigma^{\left(m-1\right)}}\right)\right)N\left(\mu_{i}|\mu_{0}^{\left(m-1\right)},{\left(\tau^{\left(m-1\right)}\right)}^{2}\right).$$
5:	**end for**
6:	Update the population mean $\mu_{0}^{\left(m\right)}$ from $$p\left(\mu_{0}|\cdot \right)\propto \prod_{i=1}^{I}N\left(\mu_{i}^{\left(m\right)}|\mu_{0},\tau^{2}\right)\pi \left(\mu_{0}\right).$$
7:	Update the between‐study standard deviation $\tau^{\left(m\right)}$ from $$p\left(\tau |\cdot \right)\propto \prod_{i=1}^{I}N\left(\mu_{i}^{\left(m\right)}|\mu_{0}^{\left(m\right)},\tau^{2}\right)\pi \left(\tau \right).$$
8:	Update the within‐study standard deviation $\sigma^{\left(m\right)}$ from $$p\left(\sigma |\cdot \right)\propto \prod_{i=1}^{I}\prod_{j=1}^{m_{i}}\mathrm{Binom}\left(k_{ij}|n_{i},\;1-\Phi \left(\frac{c_{ij}-\mu_{i}^{\left(m\right)}}{\sigma }\right)\right)\pi \left(\sigma \right).$$
9:	**end for**
10:	Return posterior samples ${\left\{\mu_{0}^{\left(m\right)},\sigma^{\left(m\right)},\tau^{\left(m\right)},\mu_{1}^{\left(m\right)},\text{…},\mu_{I}^{\left(m\right)}\right\}}_{m=1}^{M}$.

#### 2.4.3 GLMM Approximation

This approach reformulates the hierarchical normal model in a GLMM framework with a probit link. It is often computationally simpler than performing a full hierarchical likelihood or Bayesian MCMC, yet it can yield comparable parameter estimates for sufficiently large or moderate data sets. As shown below, one can rewrite the exact probability expression to match a linear predictor in a probit regression; however, other components of the GLMM fitting remain approximate (e.g., the reliance on observed proportions and a single random intercept).

For study i=1,⋯,I, let $X_{i}|\mu_{i},\sigma^{2}∼N\left(\mu_{i},\sigma^{2}\right)$ and $\mu_{i}|\mu_{0},\tau^{2}∼N\left(\mu_{0},\tau^{2}\right)$. Given ordered cut points $-\infty =c_{i0}<c_{i1}<\cdots <c_{im_{i}}<c_{i,m_{i}+1}=+\infty$, each observation falls into one of $J_{i}=m_{i}+1$ ordered intervals. Define a categorical variable $Y_{i}\in \left\{1,\cdots ,J_{i}\right\}$ determined by the intervals $\left(c_{i,j-1},c_{ij}\right]$. Define the scaled parameters

(35) $$\alpha_{i}=\frac{\mu_{i}}{\sigma },\;\;\alpha_{0}=\frac{\mu_{0}}{\sigma },\;\;\beta =\frac{1}{\sigma },\;\;\alpha_{i}∼N\left(\alpha_{0},\sigma_{\alpha }^{2}\right),\;\;\sigma_{\alpha }^{2}=\frac{\tau^{2}}{\sigma^{2}}.$$

The cumulative probabilities satisfy the cumulative probit model

(36) $$\begin{array}{ccc} & & F_{ij}\;\equiv \;\mathrm{Pr}\left(Y_{i}\leq j|\alpha_{i},\beta \right)\;=\;\mathrm{Pr}\left(X_{i}\leq c_{ij}\right)\;=\;\Phi \;\left(\beta c_{ij}-\alpha_{i}\right),\\ & & j=1,\cdots ,J_{i}-1. \end{array}$$

Equivalently,

(37) $$\Phi^{-1}\left(F_{ij}\right)\;=\;\beta \;c_{ij}-\alpha_{i},$$

a GLMM with probit link, fixed slope β on the known cut value $c_{ij}$, and a study‐specific random intercept $-\alpha_{i}$. The category probabilities are differences of cumulatives:

(38) $$\begin{array}{c} \pi_{i1}=F_{i1},\;\pi_{ij}=F_{ij}-F_{i,j-1}\;\left(2\leq j\leq J_{i}-1\right),\;\pi_{iJ_{i}}=1-F_{i,J_{i}-1}.\;\;\; \end{array}$$

Let $k_{i}=\left(k_{i1},\cdots ,k_{iJ_{i}}\right)$ be the counts with $\sum_{j}k_{ij}=n_{i}$. Conditional on $\left(\alpha_{i},\beta \right)$,

(39) $$k_{i}\;|\;\alpha_{i},\beta \;∼\;\mathrm{Multinomial}\;\left(n_{i},\;\pi_{i}\left(\alpha_{i},\beta \right)\right).$$

Together with the Gaussian random effects on $\alpha_{i}$ and priors on $\left(\alpha_{0},\beta ,\sigma_{\alpha }^{2}\right)$, this defines a cumulative‐probit GLMM. Then mapping back to $\left(\mu_{0},\sigma ,\tau \right)$. From $\left(\alpha_{0},\beta ,\sigma_{\alpha }^{2}\right)$, we recover:

(40) $$\hat{\sigma }=\frac{1}{\hat{\beta }},\;{\hat{\mu }}_{0}=\frac{{\hat{\alpha }}_{0}}{\hat{\beta }},\;\hat{\tau }=\hat{\sigma }\;\sqrt{\hat{\sigma_{\alpha }^{2}}}.$$

The GLMM formulation corresponds to a McCullagh cumulative link model when the link is probit (McCullagh 1980) with a study‐specific random intercept. The convenient approximation to replace the multinomial likelihood is a product of binomial terms at each individual cuts in each study. Because within‐study dependence among the binomial margins is ignored, and the nominal standard errors can be anticonservative. We therefore recommend a study‐level bootstrap for interval estimation.

Since the shared (fixed) slope parameter β links to the common within‐study standard deviation σ and $\alpha_{i}$ is a study‐specific random intercept when does not directly observe $p_{ij}$ but $p_{ij}^{\mathrm{obs}}=k_{ij}/n_{i}$. So we typically work with $\left(n_{i},k_{ij}\right)$ instead of $\left(n_{i},p_{ij}^{\mathrm{obs}}\right)$, which introduces an approximation step. Specifically, we employ a GLMM with a probit link

(41) $$\begin{array}{c} \frac{k_{ij}}{n_{i}}\;\approx \;\Phi \left(\alpha_{i}-\beta \;c_{ij}\right). \end{array}$$

In practice, standard software (e.g., lme4(Bates et al. 2015), glmmTMB) can fit this via:

$$\begin{array}{cc} \mathrm{cbind}(k_{ij},\;n_{i}-k_{ij}) & \;∼\;1+c_{ij}+\left(1|\mathrm{Study}\right),\\ \mathrm{family} & =\mathrm{binomial}(\mathrm{link}="\mathrm{probit}"). \end{array}$$

The term (1|Study) designates a random intercept $\alpha_{i}$, while $c_{ij}$ appears as a fixed effect with coefficient −β. A typical GLMM fit produces maximum‐likelihood estimates $\left\{{\hat{\alpha }}_{i}\right\},\hat{\beta },\hat{\sigma_{\alpha }^{2}}$, plus a fixed intercept ${\hat{\alpha }}_{0}$. We then recover the normal model parameters via:

- $\hat{\sigma }=1/\hat{\beta }$, since β=1/σ.
- ${\hat{\mu }}_{i}=\hat{\sigma }\;{\hat{\alpha }}_{i}$. Thus, each random intercept ${\hat{\alpha }}_{i}$ maps to an estimated study‐level mean ${\hat{\mu }}_{i}$.
- ${\hat{\mu }}_{0}=\hat{\sigma }\;{\hat{\alpha }}_{0}$, tying the overall intercept $\alpha_{0}$ to the population‐level mean $\mu_{0}$.
- $\tau^{2}=\sigma^{2}\;\sigma_{\alpha }^{2}\;\approx \;{\hat{\sigma }}^{2}\;\hat{\sigma_{\alpha }^{2}}$, representing the between‐study variance of $\mu_{i}$.

Fitting a probit GLMM $\left(\alpha_{0},\beta ,\sigma_{\alpha }^{2}\right)$ to these stacked binary records yields stable and asymptotically well‐behaved point estimates when there are only few cuts per study and no extreme probabilities.

## 3 Simulation Studies

In this section, we propose a comprehensive simulation design to evaluate the performance of the estimation methods under both the *fixed‐mean* and *random‐mean* settings. We focus on data generated at the study level (with either one threshold per study or multiple thresholds per study) and replicate each parameter combination multiple times to assess bias, variance, coverage probability, and overall mean‐squared error (MSE).

### 3.1 Parameter Settings and Simulation Grid

We denote the overall mean by μ (fixed mean) or $\mu_{0}$ (random mean). Without loss of generality, we fix μ=1 or $\mu_{0}=1$ in our simulations, so an MSE of 0.01 implies a typical error of 10% relative to the true mean of 1. We vary:

- I, the number of studies, in {20,50}, capturing smaller versus larger meta‐analytic settings.
- The within‐study standard deviation σ in {0.1,1}, covering high signal‐to‐noise versus moderate/noisy scenarios.
- For the random‐mean setting, we define
$$r\;=\;\frac{\tau^{2}}{\sigma^{2}+\tau^{2}}$$
  and let r∈{0.1,0.5,0.9}. Here, r=0 corresponds to the fixed‐mean setting, and larger r indicates more between‐study variability relative to within‐study variance.
- Three approaches for sampling $\left\{n_{i}\right\}$:
  1. **A (small)**: draw each $n_{i}$ uniformly from [10,150].
  2. **B (mixed)**: draw each $n_{i}$ uniformly from [10,15,000].
  3. **C (large)**: draw each $n_{i}$ uniformly from [10,000,15,000].

We perform R=1000 independent replicates under each parameter combination. Within each replicate, data are generated (Section 3.2), and all relevant estimation methods (MLE, GLM, GLMM, and MCMC) are applied to assess performance. The design is summarized in Table 1.

**TABLE 1:** Summary of simulation design factors. Each row indicates a set of parameters configurations; for each combination, we run R=1000 independent replicates.

Scenario	I	σ	r	$n_{i}$ Approach	R
*Fixed mean*	20 or 50	0.1 or 1	0	A, B, or C	1000
*Random mean*	20 or 50	0.1 or 1	0.1, 0.5, 0.9	A, B, or C	1000

### 3.2 Data Generation

We generate data differently depending on whether each study reports a **single threshold** or **multiple thresholds**.

#### 3.2.1 Single Threshold per Study – Fixed Mean

1. Generate ${\left\{n_{i}\right\}}_{i=1}^{I}$ from the chosen approach (A, B, or C).
2. Fix μ=1 and σ based on the design grid.
3. For each study i:
  - Sample a threshold $c_{i}$ from $\text{Uniform}(q_{1},q_{2})$, where $q_{1}$ and $q_{2}$ are the 20% and 80% quantiles of the study's underlying $N(\mu ,\sigma^{2})$.
  - Compute $p_{i}=1-\Phi \left(\frac{c_{i}-\mu }{\sigma }\right)$.
  - Generate $k_{i}∼\mathrm{Binomial}\left(n_{i},p_{i}\right)$ and set $p_{i}^{\mathrm{obs}}=k_{i}/n_{i}$.

#### 3.2.2 Multiple Thresholds per Study – Random Mean

In the random‐mean scenario, each study i has mean $\mu_{i}∼N\left(\mu_{0},\tau^{2}\right)$ and variance $\sigma^{2}$. Each study reports ${\left\{c_{ij}\right\}}_{j=1}^{m_{i}}$, with $m_{i}$ sampled uniformly from {5,6,7,8,9,10}.

1. Generate $\left\{n_{i}\right\}$ as in the fixed‐mean case but now fix $\mu_{0}=1$ and set σ,τ so that $\tau^{2}/\left(\sigma^{2}+\tau^{2}\right)=r$.
2. For each study i:
  - Draw $\mu_{i}∼N\left(\mu_{0},\;\tau^{2}\right)$.
  - Sample $m_{i}$ from {5,…,10} uniformly, and draw each threshold $c_{ij}∼\text{Uniform}\left(q_{1},q_{2}\right)$ (20%‐80% quantiles of $N(\mu_{i},\sigma^{2})$).
  - For each $j=1,\cdots ,m_{i}$:
$$p_{ij}\;=\;1-\Phi \;\left(\frac{c_{ij}-\mu_{i}}{\sigma }\right),k_{ij}∼\mathrm{Binomial}\left(n_{i},\;p_{ij}\right),p_{ij}^{\mathrm{obs}}=\frac{k_{ij}}{n_{i}}.$$

### 3.3 Estimation Methods and Performance Metrics

For each replicate, we apply:

- **MLE** (binomial likelihood) or **GLM** (probit) for single‐threshold data (fixed mean).
- **MLE with Integration**, **GLMM** (probit random intercept), or **Bayesian** (MCMC) for multiple‐threshold (random‐mean) data.

When the mean is fixed, we estimate μ and σ. In the random‐mean setting with multiple thresholds, we estimate $\mu_{0},\sigma ,\tau$. The performance criteria include:

1. **Bias:** Average of $\hat{\theta }-\theta_{\text{true}}$.
2. **Variance:** The sample variance of $\hat{\theta }$ across replicates, reflecting estimator stability.
3. **MSE/RMSE:** Mean‐squared (or root‐mean‐squared) error of $\hat{\theta }$.
4. **Coverage Probability:** The fraction of 95% intervals that contain the true parameter.

These statistics (bias, variance, MSE, and coverage) will be reported across all simulation configurations. Results shown on supplementary tables can visualize how each method's performance varies with study count, sample‐size distribution, and signal‐to‐noise ratio.

### 3.4 Additional Numerical and Robustness Analyses

#### 3.4.1 Quadrature Sensitivity, Computation Time, and Convergence Diagnostics

To assess numerical sensitivity to the MCAI integration width, each simulated threshold data set in the primary 36‐scenario random‐mean grid was analyzed using K∈{4,6,8,10,12}. Within each replicate, all values of K were applied to the same generated data set. For each parameter $\theta \in \mu_{0},\sigma ,\tau$, the estimate obtained at K=4,6,8, or 10 was compared with the estimate obtained from the same replicate at K=12, the largest integration width investigated: $d_{\theta }\left(K\right)=\frac{\left|\hat{\theta }\left(K\right)-\hat{\theta }\left(12\right)\right|}{\theta_{\mathrm{true}}}$. For each scenario, paired differences were summarized by their median over valid paired fits, and the resulting scenario‐specific summaries were summarized across the 36 scenarios. This design isolates sensitivity to the numerical integration width from ordinary simulation variation and from the weak identification of the separate variance components. Computation time for GH‐MLE, MCAI‐MLE, GLMM, and Bayesian MCMC were summarized across the primary random‐mean scenarios. Elapsed fitting times were evaluated on the Digital Research Alliance of Canada high‐performance computing system under the Slurm scheduler. Jobs were executed on Linux compute nodes equipped with AMD EPYC 7532 32‐Core Processor CPUs (two sockets; 64 CPU cores per node) and approximately 251 GiB of physical memory. Each simulation job requested 10 CPU cores and 20 GB of memory. All analyses were conducted using R version 4.4.0 on the x86_64pclinux‐gnu platform. The same Slurm resource‐request configuration and software environment were used for all four procedures. Therefore, the reported elapsed times are intended as empirical relative comparisons of computational burden within this common environment, rather than as hardware‐independent benchmarks. Also convergence diagnostics were included because successful return of numerical output does not necessarily imply optimizer convergence, valid numerical integration, or satisfactory MCMC mixing.

#### 3.4.2 Limited‐Data Stress Test

The primary random‐mean simulation grid considered I∈20,50 studies and the study‐size designs described above. To examine a more information‐limited setting, we conducted an additional stress‐test simulation in which both the number of studies and the within‐study sample sizes were reduced. Specifically, we considered I∈5,10, $n_{i}∼\mathrm{Uniform}\left(10,50\right)$. The remaining data‐generating mechanism was unchanged. We fixed $\mu_{0}=1$, considered σ∈0.1,1.0 and r∈0.1,0.5,0.9, and generated between‐study variation through $r=\frac{\tau^{2}}{\sigma^{2}+\tau^{2}}$. Each study contributed between 5 and 10 thresholds, as in the primary random‐mean simulation design. This yielded 12 additional scenarios to estimator bias and RMSE, each evaluated using 500 independent replicates. For each replicate, the same simulated threshold data were analyzed using GH‐MLE, MCAI‐MLE with K=10, GLMM, and Bayesian MCMC.

#### 3.4.3 Robustness to Mild Departures From Normality

We additionally assessed the robustness of the random‐mean procedures to mild misspecification of the conditional outcome distribution. Under the primary data‐generating model, the latent outcome can be written as $Y_{ij}=\mu_{i}+\sigma Z_{ij}$, $Z_{ij}∼N\left(0,1\right)$, where $\mu_{i}$ is the study‐specific mean and σ is the within‐study standard deviation. For the robustness analysis, the hierarchical structure, study‐size design, threshold construction, and values of $\mu_{0}$, σ, and τ were retained, but the standardized conditional residual distribution was varied. Three residual distributions were considered. The normal distribution, Z∼N(0,1), served as the correctly specified reference distribution. To assess sensitivity to heavier tails while preserving mean zero and unit variance, we used a standardized t distribution, $Z=\sqrt{\frac{8-2}{8}}\;T_{8}$, where $T_{8}$ denotes a t distribution with 8 degrees of freedom. Since $\mathrm{Var}\left(T_{8}\right)=8/\left(8-2\right)$, this scaling ensures that Var(Z)=1. To assess sensitivity to mild asymmetry, we used a standardized right‐skewed normal mixture. Let U∼0.9N(0,1)+0.1N(0.75,1), for which E(U)=0.075, $\mathrm{Var}\left(U\right)=1+0.1\left(1-0.1\right){\left(0.75\right)}^{2}=1.050625$. We then defined $Z=\frac{U-0.075}{\sqrt{1.050625}}$, so that E(Z)=0 and Var(Z)=1. Consequently, all three scenarios retained $E\left(Y_{ij}|\mu_{i}\right)=\mu_{i}$, $\mathrm{Var}\left(Y_{ij}|\mu_{i}\right)=\sigma^{2}$, and differed only in the shape of the conditional residual distribution. The threshold locations continued to be generated using the same central‐range construction as in the primary simulation. Conditional exceedance probabilities were then evaluated under the corresponding residual distribution. We considered I=20, study‐size design A, σ∈{0.1,1.0}, and r∈{0.1,0.5,0.9}, yielding 18 scenarios. Each scenario was evaluated using 500 independent replicates.

### 3.5 Simulation Result

The fixed‐mean scenario section evaluates the performance of two single‐threshold estimation methods—the MLE and the probit regression estimator (GLM)—under the fixed‐mean binomial‐probit framework, where all studies share a common mean $\mu_{0}$ and between‐study variance $\tau^{2}=0$. This configuration eliminates heterogeneity and isolates the core numerical behavior of both estimators when estimating $\left(\mu_{0},\sigma \right)$ from independent binomial summaries $\left(n_{i},c_{i},p_{i}\right)$. Both methods are derived from the same underlying likelihood $p_{i}=1-\Phi \;\left(\frac{c_{i}-\mu }{\sigma }\right),y_{i}∼\text{Binomial}\left(n_{i},p_{i}\right)$, where Φ(·) denotes the standard normal CDF. The MLE directly maximizes the joint binomial likelihood across all studies using the L‐BFGS‐B algorithm with a small lower bound on σ to ensure numerical stability. The probit approach reformulates the problem as a GLM with probit link by expanding each study's binomial data into individual binary outcomes, and estimates parameters via ordinary least squares on the probit scale. Both approaches optimize equivalent likelihood structures, but through different numerical mechanisms.

**FIGURE 1:**
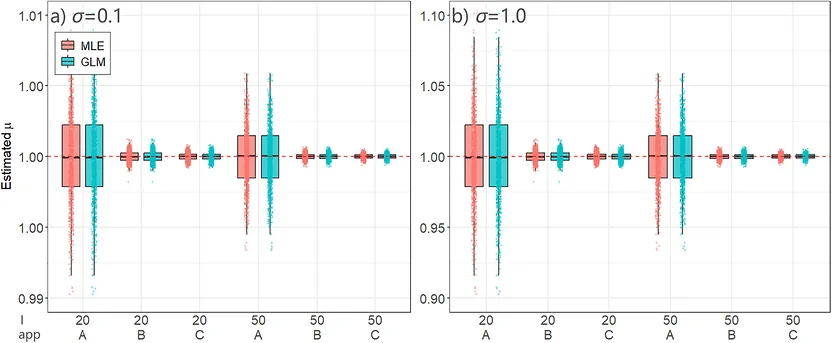
Boxplots summarize 1000 simulation replicates for the likelihood‐based MLE and the probit GLM estimator across combinations of the number of studies (I=20,50), within‐study standard deviation (σ=0.1,1.0), and study‐size allocation designs (A–C). Panels (a) and (b) correspond to σ=0.1 and σ=1.0, respectively. The dashed horizontal line denotes the true value $\mu_{0}=1.0$.

Figure 1 displays the sampling distributions of the estimated common mean ${\hat{\mu }}_{0}$ under the fixed‐mean model across all simulation settings, comparing the likelihood‐based MLE and the probit GLM estimator. Results are shown separately for the two within‐study standard deviation levels (panel a: σ=0.1; panel b: σ=1.0), and within each panel are grouped by the number of studies (I=20 vs. I=50) and the study‐size allocation design (A–C). Across all groups, both estimators remain tightly centered around the dashed reference line at the true value $\mu_{0}=1.0$, and no systematic upward or downward shift is visible for either method. The most prominent change across settings is a reduction in sampling variability as I increases: for each design (A, B, and C), the I=50 boxplots are noticeably narrower than the corresponding I=20 boxplots, indicating more stable estimation with more studies. In addition, the panel comparison shows the expected inflation in dispersion when moving from σ=0.1 to σ=1.0: for fixed I and design, the interquartile ranges and tails are wider in panel (b) than panel (a), while the central locations remain essentially unchanged. Differences among the three allocation designs are comparatively small; design A shows the widest spreads (especially at I=20), whereas designs B and C yield more concentrated distributions, and these design‐related differences diminish further when I=50. Numerical summaries are provided in Tables S1–S4 and align closely with the visual patterns in Figure 1. Table S1 reports empirical bias and indicates that both MLE and GLM exhibit negligible bias across all fixed‐mean scenarios, with values extremely close to zero under every combination of I, σ, and design. Table S2 reports the empirical variance of ${\hat{\mu }}_{0}$ and shows a clear reduction when the number of studies increases from I=20 to I=50 for both methods, consistent with the visibly tighter boxplots at I=50. The variances are also larger when σ=1.0 than when σ=0.1, matching the wider sampling distributions in panel (b). Table S3 reports the empirical MSE and mirrors the same ordering: MSE decreases substantially from I=20 to I=50 and increases with σ, while remaining very similar between the two estimators within each scenario because bias is near zero and performance is dominated by sampling variability. Finally, Table S4 reports empirical coverage probabilities of nominal 0.95 confidence intervals. Coverage remains close to the nominal 0.95 level across all scenarios for both MLE and GLM, with no consistent under‐ or overcoverage pattern across σ, I, or designs A–C, indicating that uncertainty quantification is stable under the fixed‐mean model.

**FIGURE 2:**
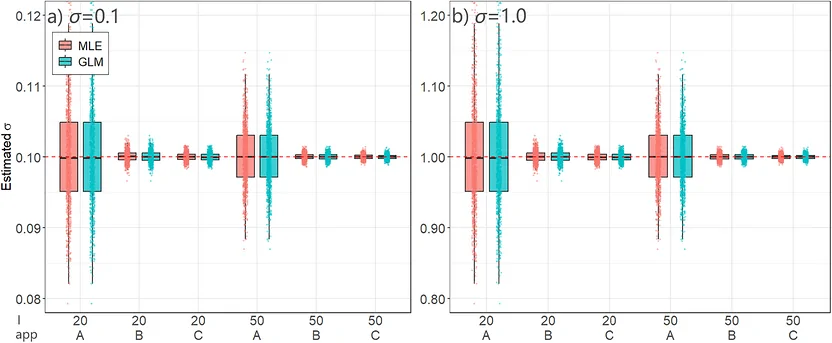
Boxplots summarize 1000 simulation replicates for the likelihood‐based MLE and the probit GLM estimator across combinations of the number of studies (I=20,50), true within‐study standard deviation (σ=0.1,1.0), and study‐size allocation designs (A–C). Panels (a) and (b) correspond to σ=0.1 and σ=1.0, respectively. The dashed horizontal line indicates the true value of σ.

Figure 2 displays the sampling distributions of the within‐study standard deviation estimator $\hat{\sigma }$ under the fixed‐mean model across all simulation scenarios. Results are shown for the likelihood‐based MLE and the probit GLM estimator under varying numbers of studies (I=20,50), true within‐study standard deviation (σ=0.1 or 1.0), and three study‐size allocation designs (A–C). Each boxplot summarizes 1000 simulation replicates, and the dashed horizontal line denotes the true value of σ in each panel. Across all scenarios, both estimators are centered close to the true σ, with no systematic deviation in the median estimates. Sampling variability is largest when I=20 under design A, particularly for the low‐study regime, and is visibly reduced when the number of studies increases to I=50. Differences among study‐size allocation designs are modest for designs B and C, and become negligible as I increases. Comparing panels (a) and (b), the higher‐variance setting (σ=1.0) exhibits substantially wider sampling distributions than the low‐variance setting (σ=0.1), while the central location of the estimates remains stable for both estimators. This pattern is consistent across all designs and both values of I. Numerical summaries of estimator performance are provided in Tables S5–S8. Table S5 reports the empirical bias of $\hat{\sigma }$ and shows that both estimators exhibit small bias across all fixed‐mean scenarios, with larger bias observed under design A and reduced bias when I=50. Tables S6 and S7 summarize the empirical variance and MSE of $\hat{\sigma }$, respectively. Both quantities decrease substantially as the number of studies increases from I=20 to I=50, and are consistently larger when σ=1.0 than when σ=0.1, reflecting the wider sampling distributions observed in Figure 2. Table S8 reports empirical coverage probabilities of nominal 0.95 confidence intervals, which remain close to the nominal level for both MLE and GLM across all scenarios, indicating appropriate uncertainty quantification under the fixed‐mean model.

Across all simulated conditions, the two methods yielded nearly identical point estimates for both $\mu_{0}$ and σ. The boxplots show that ${\hat{\mu }}_{0}$ values cluster tightly around the true value of $\mu_{0}=1.0$ with minimal bias, regardless of sample size (I=20 or 50) or study‐size configuration (A, B, or C). Likewise, $\hat{\sigma }$ estimates are unbiased at both σ=0.1 and σ=1.0, with only minor increases in variability as σ grows. The MLE exhibited slightly narrower dispersion under small‐sample settings, reflecting its optimal efficiency in finite samples, whereas the GLM achieved nearly the same precision at a fraction of the computational cost. The strong concordance between MLE and GLM arises from the fact that the probit GLM is the asymptotic equivalent of the binomial MLE when the latent model is correctly specified. In the fixed‐mean design, there is no random‐effect integration, so both estimators operate on the same well‐conditioned log‐likelihood surface. Consequently, the bias and variance patterns of $\hat{\mu }$ and $\hat{\sigma }$ coincide closely, and both estimators converge rapidly to the true parameters. The MLE benefits from exact likelihood optimization and thus provides the theoretical benchmark, while the GLM offers high numerical stability and speed due to its closed‐form iterative fitting under the canonical probit link. The results confirm that, for homogeneous data sets without between‐study variation, the probit‐based GLM can serve as a reliable and computationally efficient substitute for the full MLE. The MLE remains preferable when exact likelihood inference or small‐sample accuracy is essential, while the GLM is advantageous for large‐scale or simulation‐intensive applications. Overall, both estimators are unbiased, consistent, and efficient in recovering the true $\left(\mu_{0},\sigma \right)$, establishing a solid baseline for extending to hierarchical random‐mean models.

Random‐mean scenario compared three estimation strategies for the hierarchical model with random study effects, namely, the MLE based on GH integration, the GLMM approximation, and the fully Bayesian MCMC estimator implemented in Stan. Although all three methods target the same hierarchical likelihood, their numerical behavior differs markedly across parameter regimes, particularly with respect to the ratio $r=\tau^{2}/\left(\sigma^{2}+\tau^{2}\right)$ and the within‐study sample size $n_{i}$. Analytically, integrating over the study effects $\mu_{i}∼N\left(\mu_{0},\tau^{2}\right)$ yields marginal interval probabilities that depend on the combined probit scale $S=\sqrt{\sigma^{2}+\tau^{2}}$ rather than on σ and τ separately. Consequently, the log‐likelihood $\ell \left(\mu_{0},\sigma ,\tau \right)=f\left(S,\mu_{0}\right)$ possesses a ridge along $\sigma^{2}+\tau^{2}=\text{const}$, implying that the data identify S strongly but provide weak curvature for the decomposition of $S^{2}$ into within‐ and between‐study components. When r is small (τ≪σ), the ridge lies close to the τ=0 axis and σ is well‐identified; when r is large, many (σ,τ) combinations produce nearly identical marginal fits, leading to nonidentifiability unless additional curvature is introduced through model structure, priors, or numerical regularization.

**FIGURE 3:**
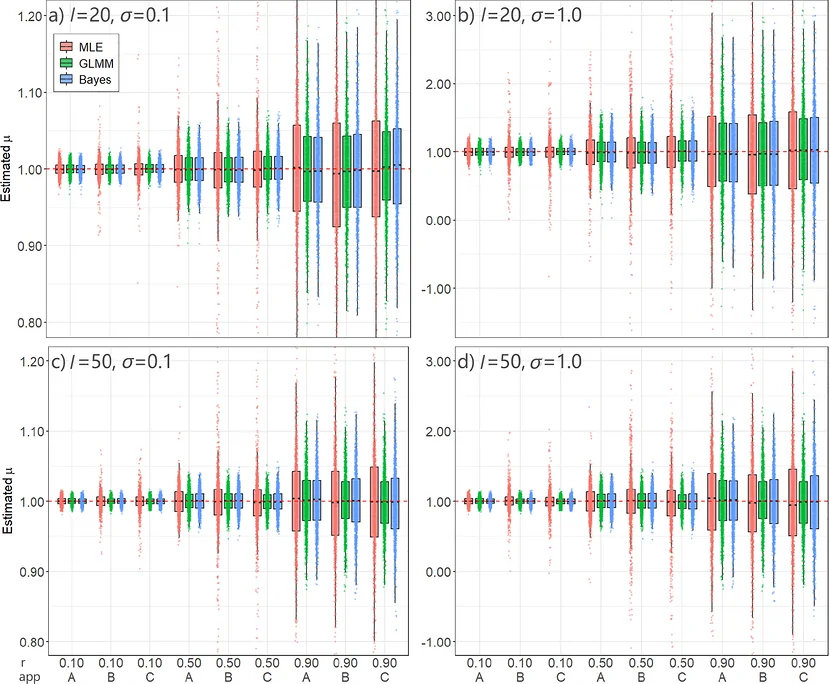
Boxplots summarize 1000 simulation replicates for the integrated likelihood‐based MLE, the probit GLMM estimator, and the Bayesian MCMC approach across combinations of the number of studies (I=20,50), within‐study standard deviation (σ=0.1,1.0), heterogeneity levels (r=0.1,0.5,0.9), and study‐size allocation designs (A–C). Panels (a)–(d) correspond to the four combinations of I and σ. The dashed horizontal line indicates the true value $\mu_{0}=1.0$.

Figure 3 displays the sampling distributions of the estimated population‐level mean ${\hat{\mu }}_{0}$ under the random‐mean model across all simulation scenarios. Results are shown for the integrated likelihood‐based MLE, the probit GLMM, and the Bayesian MCMC approach, under varying numbers of studies (I=20,50), within‐study standard deviation (σ=0.1 or 1.0), heterogeneity levels (r=0.1,0.5,0.9), and three study‐size allocation designs (A–C). Each boxplot summarizes 1000 simulation replicates, and the dashed horizontal line denotes the true value $\mu_{0}=1.0$. Across all panels, the central tendency of ${\hat{\mu }}_{0}$ remains close to the true value for all three methods. However, increasing heterogeneity leads to a pronounced increase in sampling variability. In particular, as r increases from 0.1 to 0.9, the spread of the integrated MLE estimates widens substantially, with visibly heavier tails, especially under design A. The GLMM estimator remains well centered with comparatively stable dispersion across heterogeneity levels, while the Bayesian MCMC results are generally similar to GLMM but exhibit occasional extreme draws in a subset of scenarios. Comparing panels (a) and (b) to (c) and (d), increasing the number of studies from I=20 to I=50 results in a clear reduction in sampling variability for all three methods across all heterogeneity levels. Likewise, comparing low‐variance and high‐variance regimes, scenarios with σ=1.0 exhibit markedly wider sampling distributions than those with σ=0.1, although the median behavior remains stable. Numerical summaries are provided in Tables S9–S12. Table S9 reports empirical bias and shows that biases of ${\hat{\mu }}_{0}$ are generally small in magnitude across all random‐mean scenarios. Tables S10 and S11 summarize the empirical variance and MSE, both of which increase with heterogeneity (r) and within‐study variability (σ), and decrease when the number of studies increases from I=20 to I=50. Table S12 reports empirical coverage probabilities of nominal 0.95 confidence intervals: coverage for the GLMM estimator remains close to the nominal level across scenarios, whereas the integrated MLE exhibits undercoverage in high‐heterogeneity settings, and the Bayesian MCMC approach shows slightly inflated coverage in a small number of scenarios with increased dispersion.

**FIGURE 4:**
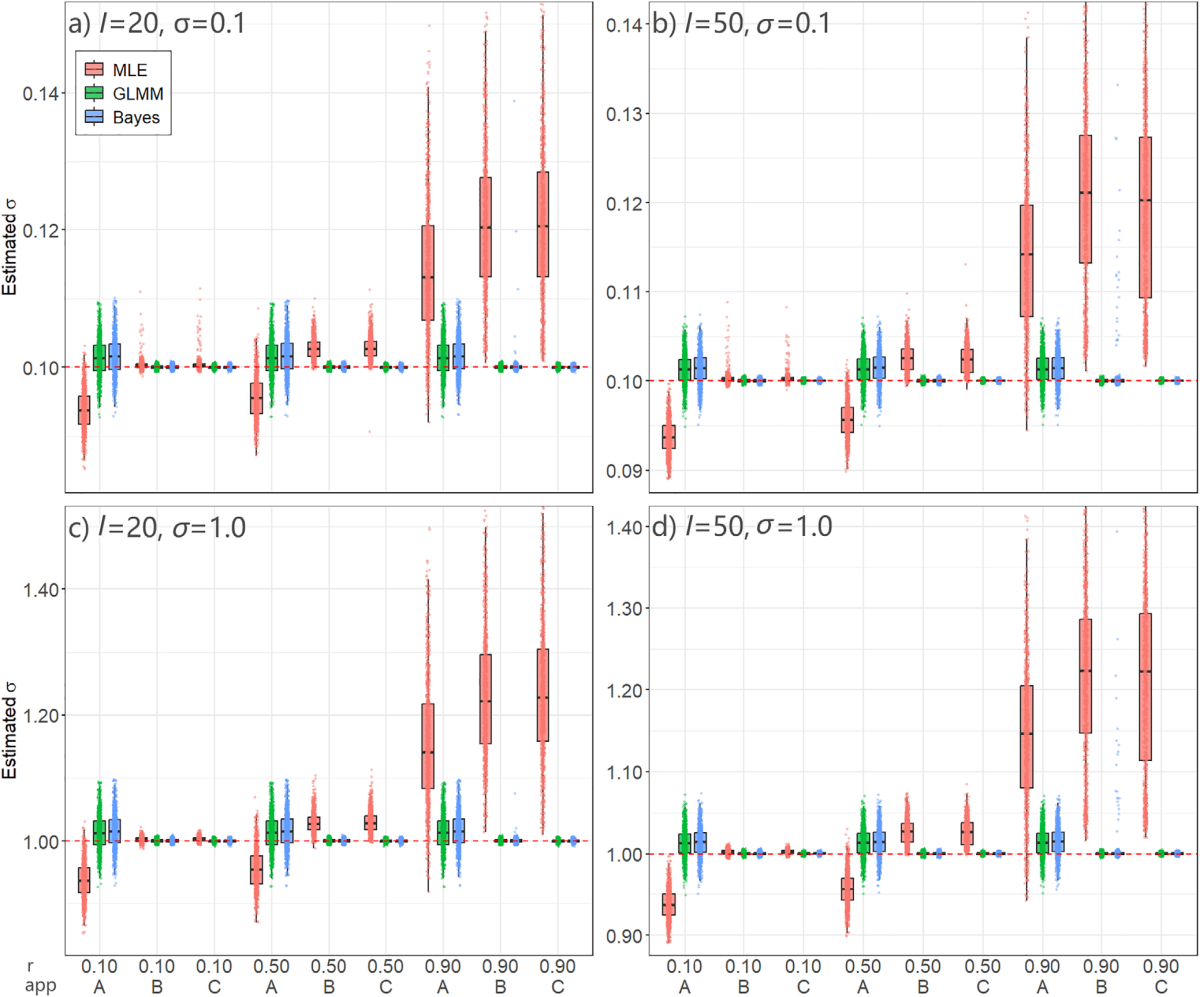
Sampling distributions of the estimated within‐study standard deviation $\hat{\sigma }$ under the random‐mean model. Boxplots summarize 1000 simulation replicates for the likelihood‐based integrated MLE, the probit GLMM, and the Bayesian MCMC estimator across combinations of the number of studies (I=20,50), true within‐study standard deviation (σ=0.1,1.0), heterogeneity levels (r=0.1,0.5,0.9), and study‐size allocation designs (A–C). Panels (a)–(b) correspond to σ=0.1, and panels (c)–(d) to σ=1.0. The dashed horizontal line denotes the true value of σ in each scenario.

Figure 4 displays the sampling distributions of the estimated within‐study standard deviation $\hat{\sigma }$ under the random‐mean model across all simulation scenarios, stratified by the true σ (panels a–b: σ=0.1; panels c–d: σ=1.0) and the number of studies (left: I=20; right: I=50). Each boxplot summarizes 1000 simulation replicates for a given combination of heterogeneity (r=0.1,0.5,0.9) and study‐size allocation design (A–C), and the dashed horizontal line denotes the true value of σ. Across all panels, the probit‐GLMM and Bayesian (MCMC) estimators remain tightly centered around the true value of σ, with relatively stable dispersion as heterogeneity increases. In contrast, the likelihood‐based MLE exhibits a pronounced upward shift and rapidly increasing variability as r increases, particularly at r=0.9, where the sampling distributions show long right tails and substantial overestimation of σ. This behavior is visible under both variance regimes and is most pronounced under design A. Increasing the number of studies from I=20 to I=50 leads to a clear reduction in sampling variability for all three methods. However, while this increase in I substantially stabilizes the GLMM and MCMC estimators, the heterogeneity‐driven inflation of the MLE remains clearly visible even when I=50, especially in the high‐heterogeneity settings. Differences among designs A–C are comparatively minor for GLMM and MCMC, and become noticeable primarily for the MLE under moderate to high heterogeneity, where overall variability dominates design‐specific effects. Numerical summaries corresponding to Figure 4 are reported in Tables S13–S16. Table S13 shows that empirical bias for GLMM and MCMC remains close to zero across all 36 scenarios, whereas the MLE bias increases sharply with heterogeneity. For example, when σ=0.1, the MLE bias grows from near‐zero magnitude at r=0.1 to approximately 0.08–0.10 at r=0.9; when σ=1.0, this pattern scales upward, with MLE bias reaching values on the order of 0.8–1.0 at r=0.9, consistent with the elevated medians observed in panels (c)–(d). Table S14 reports empirical variances, which remain small and stable for GLMM and MCMC across heterogeneity levels and decrease further as I increases, particularly under designs B and C. In contrast, the MLE variance increases rapidly with r. These trends are mirrored in Table S15, where the MLE MSE increases dramatically with heterogeneity, while GLMM and MCMC maintain consistently low MSE across all scenarios. Finally, Table S16 summarizes empirical coverage probabilities of nominal 0.95 confidence intervals. Coverage for GLMM and MCMC remains close to the nominal level across scenarios, whereas the MLE exhibits substantial undercoverage that worsens as heterogeneity increases, reaching very low values in several high‐r settings. Overall, Tables S13–S16 quantitatively confirm the visual patterns in Figure 4: under the random‐mean model, GLMM and MCMC provide stable estimation and reliable uncertainty quantification for σ, while the MLE becomes increasingly biased and unstable as between‐study heterogeneity strengthens.

**FIGURE 5:**
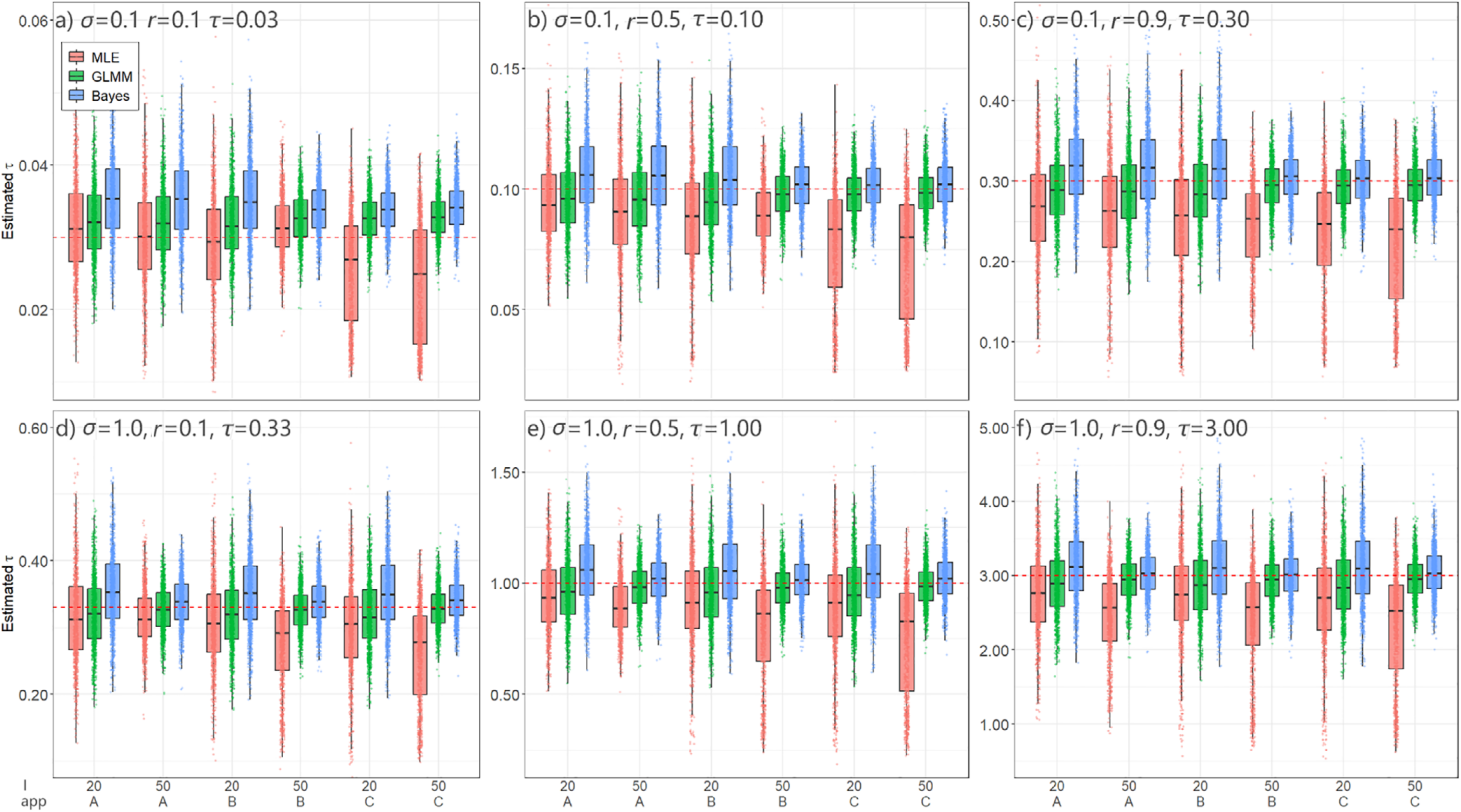
Sampling distributions of the estimated between‐study standard deviation τ under the random‐mean model. Boxplots summarize 1000 simulation replicates for the integrated MLE, probit‐GLMM, and Bayesian MCMC estimators across combinations of number of studies (I=20,50), within‐study standard deviation (σ=0.1,1.0), heterogeneity level (r=0.1,0.5,0.9), and study‐size allocation designs (A–C). The dashed horizontal line indicates the true value of τ implied by (σ,r).

Figure 5 summarizes the sampling distributions of the estimated between‐study standard deviation $\hat{\tau }$ under the random‐mean model across all simulation scenarios. Panels (a)–(f) stratify results by the true within‐study standard deviation (σ=0.1 in panels a–c; σ=1.0 in panels d–f) and heterogeneity level (r=0.1,0.5,0.9), with each boxplot corresponding to a combination of number of studies (I=20,50) and study‐size allocation design (A–C). Each boxplot summarizes 1000 simulation replicates, and the dashed horizontal line indicates the true value of τ implied by (σ,r). Across all panels, increasing heterogeneity leads to substantially wider sampling distributions for all methods, reflecting the growing difficulty of estimating between‐study variability as r increases. When heterogeneity is weak (r=0.1), estimates of τ concentrate near the true value with relatively small dispersion, particularly for σ=0.1. As r increases to 0.5 and 0.9, the variability of $\hat{\tau }$ increases markedly, with much heavier tails visible in the sampling distributions. Clear method‐specific differences emerge as heterogeneity strengthens. The likelihood‐based MLE exhibits a pronounced downward shift in median estimates, especially under moderate to strong heterogeneity. This underestimation becomes most severe when σ=1.0 and r=0.9, where the MLE displays substantial negative bias and very wide right‐skewed distributions. In contrast, the GLMM and Bayesian (MCMC) estimators remain well centered around the reference line across most scenarios, with comparatively stable dispersion as r increases. Increasing the number of studies from I=20 to I=50 reduces sampling variability for all three methods, as seen by narrower boxplots across panels. However, even with I=50, the heterogeneity‐driven bias and dispersion of the MLE remain clearly visible under high r, whereas GLMM and MCMC continue to exhibit stable central behavior. Differences among study‐size allocation designs A–C are generally small relative to the dominant effects of heterogeneity level and estimation method. Numerical summaries of estimator performance are reported in Tables S17–S20. Table S17 confirms that the MLE exhibits consistently negative bias that increases rapidly with heterogeneity, while GLMM and MCMC biases remain small across all 36 scenarios. Tables S18 and S19 show that both the variance and MSE of $\hat{\tau }$ increase with r and with σ, with GLMM typically achieving smaller dispersion and error than the MLE. Finally, Table S20 reports empirical coverage probabilities of nominal 95% confidence intervals, indicating near‐nominal coverage for GLMM and MCMC across scenarios, whereas MLE intervals exhibit substantial undercoverage that worsens as heterogeneity strengthens and the number of studies increases.

The poor variance‐component behavior of the GH‐based likelihood maximization in high‐heterogeneity regimes is best understood as an interaction between weak identification and numerical likelihood evaluation. As shown in the simulation result (via the combined scale $S=\sqrt{\sigma^{2}+\tau^{2}}$), the marginal likelihood exhibits a ridge in the (σ,τ) plane: many (σ,τ) pairs yield nearly indistinguishable fits whenever they preserve S. In this situation, maximizing the *approximated* marginal log‐likelihood can be unstable along the ridge because small approximation errors may induce a nonnegligible tilt in the objective, shifting the maximizer toward different decompositions of $S^{2}$ into $\sigma^{2}$ and $\tau^{2}$. This explains why the GH‐MLE can display systematic inflation of $\hat{\sigma }$ and shrinkage of $\hat{\tau }$ as r increases, and why modifying the approximation can change the direction and magnitude of the bias without eliminating it.

Supplementary Figure 1 shows that the MCAI estimates were numerically insensitive to the integration width over the investigated range. The scenario‐median paired differences decreased sharply from K=4 to K=6 for all three parameters. At K=8 and K=10, the differences from the corresponding K=12 estimates were at, or close to, numerical precision. Thus, increasing the integration domain beyond K=10 did not materially change the reported MCAI estimates. These results support K=10 as a conservative default. They establish numerical stability with respect to the adaptive integration width, but do not imply that increasing K resolves the underlying weak identification of the separate variance components σ and τ.

The following results compare the computational burden and numerical reliability diagnostics for four procedures. Let $J_{i}=m_{i}+1$ denote the number of interval probabilities in study i. For a fixed parameter value, GH‐MLE evaluates the study‐specific integrand at a fixed number of nodes and therefore has a numerical cost proportional to $G\sum_{i}J_{i}$ per likelihood evaluation, where G=20 in the present simulations. MCAI evaluates the same marginal likelihood but additionally performs a study‐specific mode search, local‐curvature calculation, and adaptive numerical integration. Its computational burden is proportional to $\sum_{i}\left(a_{i}+q_{i}\right)J_{i}$, where $a_{i}$ and $q_{i}$ are the integrand evaluations required for mode/curvature calculations and adaptive integration, respectively. These quantities vary with integrand concentration, the integration width K, and numerical tolerances. The GLMM was fitted using an optimized Laplace‐type mixed‐model routine and did not require a separate general‐purpose adaptive integral for each study at every outer optimization step. Bayesian MCMC required repeated likelihood‐gradient evaluations over multiple Hamiltonian Monte Carlo chains and iterations; its total computation therefore depended on warm‐up, retained iterations, and the number of leapfrog steps. For GH‐MLE, MCAI‐MLE, and GLMM, we recorded optimizer convergence status; we additionally recorded penalized or invalid likelihood evaluations for MCAI‐MLE and singular‐fit status for GLMM. For Bayesian MCMC, we recorded runtime errors, the maximum $\hat{R}$ across monitored parameters, the minimum ESS, divergent transitions, and maximum‐treedepth warnings. Table S21 summarizes computation time and fitting diagnostics across the primary 36‐scenario simulation grid. The empirical computational ordering was GLMM < GH‐MLE ≪ MCAI‐MLE < Bayesian MCMC. GLMM was fastest, with a median scenario‐level fitting time of 0.057 s per replicate. GH‐MLE required 2.144 s, reflecting repeated fixed‐node quadrature evaluations during likelihood optimization. MCAI‐MLE required 42.516 s, approximately 19.8 times the GH‐MLE time, because every outer optimization step additionally involved study‐specific mode searches, curvature calculations, and adaptive numerical integrations. Bayesian MCMC required 67.742 s, approximately 31.6 times the GH‐MLE time, because posterior sampling repeatedly evaluated likelihood gradients across chains and iterations. Numerical fitting issues were observed for all optimization‐based procedures. Optimizer nonconvergence occurred in 6.1% of GH‐MLE fits, 5.6% of MCAI‐MLE fits, and 6.7% of GLMM fits. MCAI‐MLE additionally produced an invalid or penalized likelihood in 9.7% of fits, whereas no GLMM singular fits were observed. Bayesian MCMC returned finite output in 99.98% of attempted fits, but standard mixing diagnostics were frequently unsatisfactory in the primary simulation: 91.6% of fits had $\hat{R}>1.01$, 75.4% had minimum ESS below 100, and 65.5% generated maximum‐treedepth warnings. Divergent transitions were rare (0.09%). Thus, successful numerical return should not be interpreted as confirmed MCMC convergence. The Bayesian point estimates are retained as a secondary numerical comparison, but the results do not support a claim that the MCMC implementation was uniformly reliable across all weakly identified variance‐component settings.

The limited‐data stress test was evaluated relative to the primary random‐mean approach A simulations, in which I=20 or 50 studies were available. Because the stress‐test settings reduced both the number of studies to I=5 or 10 and the within‐study sample sizes to $n_{i}∼\mathrm{Uniform}\left(10,50\right)$, the comparison reflects a combined reduction in available information. When heterogeneity was low, estimation of the population mean remained accurate even under the smaller design. For example, when σ=0.1 and r=0.1, the RMSE of ${\hat{\mu }}_{0}$ was approximately 0.01 at I=10, compared with approximately 0.007–0.008 in the corresponding primary I=20 approach A setting. In the same setting, RMSE values for $\hat{\sigma }$ were between 0.008 and 0.012, indicating that the within‐study scale remained reasonably estimable. In contrast, τ was less precisely estimated: when the true value was τ=0.033, RMSE values of approximately 0.009–0.013 at I=10 corresponded to relative errors of roughly 27%–39%. Thus, with 10 studies and low heterogeneity, $\mu_{0}$ and σ can still be estimated with useful accuracy, whereas τ should be interpreted mainly as a rough measure of between‐study variation. The deterioration became substantial when the number of studies was reduced to I=5 or when heterogeneity was high. For example, under σ=0.1 and r=0.9, the RMSE of ${\hat{\mu }}_{0}$ increased from approximately 0.065–0.106 in the primary I=20 approach A setting to 0.132–0.161 for GH‐MLE, MCAI‐MLE, and GLMM at I=5, while the Bayesian RMSE reached 0.925. When σ=1.0 and r=0.9, RMSE for ${\hat{\mu }}_{0}$ exceeded 1 for all methods at I=5, indicating that the population mean was no longer estimated with useful precision. Estimation of σ and τ was even more vulnerable to sparse information, with occasional extreme estimates for MCAI‐MLE and Bayesian MCMC in the most difficult settings. Overall, under the most limited design, GH‐MLE, MCAI‐MLE, and GLMM retained reasonably accurate estimation of $\mu_{0}$, and of σ when heterogeneity was low to moderate; however, τ was not estimated with sufficient precision by any method, while Bayesian MCMC could be highly unstable for σ in the low‐σ settings. When heterogeneity was strong (r=0.9), none of the methods provided reliably precise estimation of the separate variance components σ and τ, and estimation of $\mu_{0}$ also became inaccurate. Complete bias and RMSE results are provided in Table S22.

The normal, standardized $t_{8}$, and mildly right‐skewed mixture scenarios had identical values of I, σ, r, study‐size design, and threshold construction. Therefore, the RMSE ratios in Table S23 directly quantify the effect of changing only the conditional residual distribution: a ratio close to one indicates little loss of accuracy relative to the normal‐reference scenario. The mildly right‐skewed mixture had little effect on GH‐MLE, GLMM, and Bayesian MCMC: their mixture‐to‐normal RMSE ratios ranged from 0.87 to 1.10 across $\mu_{0}$, σ, and τ. MCAI‐MLE showed the same pattern except in the highest‐heterogeneity setting with σ=0.1 and r=0.9, where its RMSE ratios were 1.73 for ${\hat{\mu }}_{0}$ and 2.88 for $\hat{\tau }$. In contrast, the standardized $t_{8}$ distribution affected estimation of the within‐study scale σ more substantially. The $t_{8}$‐to‐normal RMSE ratios for $\hat{\sigma }$ were 2.45–2.81 for GLMM and 2.29–2.64 for Bayesian MCMC across all six settings; GH‐MLE and MCAI‐MLE also showed approximately twofold increases in several low‐ and moderate‐heterogeneity settings, with an extreme MCAI‐MLE ratio of 52.58 when σ=0.1 and r=0.9. Thus, the methods were broadly robust to the mild skewness considered here, and estimation of $\mu_{0}$ and τ was generally less sensitive to heavy tails; however, inference on σ was materially affected by the heavy‐tailed departure, particularly in high‐heterogeneity settings.

## 4 Real Data Application

We illustrate the proposed framework using serum albumin measured at baseline in the UK Biobank (field 30600). Albumin (g/L) is a routinely collected clinical biomarker reflecting liver function, nutritional status, and systemic inflammation, and it provides a suitable continuous phenotype for assessing the practical behavior of probit‐based inference from threshold‐type summaries. After standard quality control and removal of missing values, the analysis included N=431,857 participants, with empirical mean $\bar{y}=45.21$, standard deviation SD=2.63, and between center variance τ=0.23. The raw data have 20 groups of patients (each patient belongs to one of 20 labels), so we treated it as 20 independent studies with nonoverlapping subgroups, denoted as study size I=20.

### 4.1 Distributional Assessment

Within each subgroup and in the pooled sample, the empirical distribution of albumin is close to Gaussian (Supplementary Figure 2). The pooled distribution is nearly symmetric (skewness =−0.036) with moderate tail heaviness (kurtosis =3.71), supporting the working normality assumption for the latent outcome. Minor deviations are confined to the extreme tails and are typical in very large samples; these do not materially affect inference when thresholds are chosen within the central range.

### 4.2 Construction of Threshold Summaries

Although individual‐level measurements are available, we construct threshold‐type summaries to align with the data structures assumed in Scenarios 1 and 3. For Scenario 1 (fixed mean with a single threshold), each study is summarized by one cut point $c_{i}$ and the corresponding exceedance count $k_{i}$. For Scenario 3 (random mean with multiple thresholds), each study is summarized by ordered cut points $\left\{c_{ij}\right\}$ and interval counts $\left\{k_{ij}\right\}$. In this application, thresholds were chosen from Uniform distribution that quantiles between 20% and 80%, ensuring non‐degenerate binomial proportions and numerical stability.

### 4.3 Estimation Results

Table 2 shows that the latent distribution recovered from the threshold summaries closely reproduced the patient‐level benchmark. Under the fixed‐mean specification, the MLE and probit GLM gave an estimated mean of 45.25 g/L, only 0.04 g/L above the empirical mean of 45.21 g/L. Under the random‐mean specification, the estimated population mean ranged from 45.16 to 45.24 g/L across the three methods. Thus, dichotomized threshold summaries recovered the central location of the albumin distribution with an absolute discrepancy of no more than 0.05 g/L. The estimated scales have a similarly direct interpretation. For the GH‐MLE and GLMM, the within‐group standard deviation was 2.59 and 2.57 g/L, respectively, and the corresponding between‐group standard deviation was only 0.23 and 0.22 g/L. The resulting marginal scale, $S=\sqrt{\sigma^{2}+\tau^{2}}$, was 2.60 g/L for GH‐MLE and 2.58 g/L for GLMM, close to the pooled patient‐level standard deviation of 2.63 g/L. The estimated between‐group variance share, $\tau^{2}/\left(\sigma^{2}+\tau^{2}\right)$, was approximately 0.8% for GH‐MLE and 0.7% for GLMM. Therefore, nearly all albumin variation in this application occurred among individuals within the 20 groups, while systematic differences in mean albumin level between groups were small. Equivalently, a one‐standard‐deviation group‐level shift was about 0.22–0.23 g/L, less than one‐tenth of the within‐group standard deviation. The Bayesian MCMC analysis produced comparable estimates of the population mean and within‐group scale, but a smaller estimate of τ (0.08 g/L). This difference should be interpreted cautiously: although the total sample size is large, the variance decomposition is informed by only 20 independent groups, so the separate values of σ and τ are less precisely identified than the population mean and total scale. Nevertheless, all approaches support the same practical conclusion: threshold summaries can accurately recover the overall albumin distribution in this large application, and the residual between‐group heterogeneity is small.

**TABLE 2:** UK Biobank albumin application: parameter estimates under fixed‐mean (Scenario 1) and random‐mean (Scenario 3) models. In the fixed‐mean model, the between‐study variance is fixed at τ=0.

Model/Method	$\mu \;\text{or}\;\mu_{0}$	σ	τ
Fixed‐mean MLE	45.25	2.66	0
Fixed‐mean GLM	45.25	2.66	0
Random‐mean GH‐MLE	45.16	2.59	0.23
Random‐mean GLMM	45.19	2.57	0.22
Random‐mean Bayesian MCMC	45.24	2.58	0.08
Patient level data Estimate (ref/truth)	45.21	2.63	0.23

## 5 Discussion

**Fixed‐mean designs: equivalence of GLM and binomial MLE**. Under the fixed‐mean setting ($\tau^{2}=0$), both the binomial maximum likelihood estimator and the probit GLM provide essentially identical inference for $\left(\mu_{0},\sigma \right)$. This agreement is expected because both procedures target the same binomial‐probit likelihood and differ primarily in numerical implementation. In particular, under correct model specification and standard regularity conditions, probit GLM fitting is asymptotically equivalent to maximizing the corresponding binomial likelihood on the probit scale, while benefiting from robust and optimized GLM routines. Consistent with this, our simulations show that both estimators are unbiased and stable across the study‐count and sample‐size regimes considered, with the MLE offering a slight small‐sample efficiency advantage and the GLM providing comparable accuracy at substantially lower computational cost. These results serve as a baseline: when heterogeneity is negligible, likelihood geometry is well‐conditioned and method choice is mostly computational.

**Random effects: ridge geometry and the interaction of weak identification with likelihood approximation**. When study‐specific means are modeled as random effects, inference is shaped by the identifiability structure created by thresholding and marginalization. Integrating over $\mu_{i}∼N\left(\mu_{0},\tau^{2}\right)$ collapses within‐ and between‐study variation into the combined probit scale $S=\sqrt{\sigma^{2}+\tau^{2}}$, inducing a ridge in the marginal likelihood along $\sigma^{2}+\tau^{2}=\text{const}$. Consequently, $\mu_{0}$ and S are typically well‐identified, whereas the decomposition of $S^{2}$ into $\left(\sigma^{2},\tau^{2}\right)$ can be weakly identified unless the threshold configuration provides sufficient within‐study information (e.g., multiple cut points spanning the central probit range) and the design yields nondegenerate binomial proportions.

Ridge geometry alone does not imply that inference must fail. The practical instability observed for likelihood maximization arises from the *interaction* between ridge‐like geometry (i.e., weak curvature in the (σ,τ) direction) and numerical approximation of the marginal likelihood. In high‐heterogeneity regimes (large $r=\tau^{2}/\left(\sigma^{2}+\tau^{2}\right)$), many (σ,τ) pairs fit the data nearly equally well as long as S is preserved. When the marginal likelihood is evaluated through numerical integration, small deterministic integration errors can introduce a slight tilt along the ridge; if this perturbation is comparable to the (weak) curvature in the ridge direction, the maximizer can move substantially in (σ,τ) while leaving $\hat{S}$ nearly unchanged. This mechanism explains the systematic pattern seen in our simulations: as r increases, the GH MLE tends to drift toward inflated $\hat{\sigma }$ and attenuated $\hat{\tau }$, despite still achieving the maximum of the GH‐approximated objective.

**Numerical sensitivity of marginal‐likelihood evaluation**. To reduce this sensitivity, we introduced an MCAI strategy that recenters and adapts the quadrature to the region contributing most to the marginal likelihood (Supplementary Section 1). Empirically, MCAI can reduce directional bias relative to fixed‐node GH in regimes where the dominant mass of the integrand is not well covered by a fixed set of quadrature nodes, and it can improve numerical stability when $n_{i}$ is large (Supplementary Figures 3–5). However, MCAI does not eliminate the fundamental ridge: it mitigates one source of approximation error but does not create new information for separating σ and τ. As a result, variance‐component bias can persist, and its direction may depend on the approximation strategy.

A useful diagnostic perspective is to view each approximation as defining its own objective function. Let ℓ(θ) denote the (intractable) marginal log‐likelihood with $\theta =(\mu_{0},\sigma ,\tau )$, and let ${\tilde{\ell }}_{\mathrm{GH}}\left(\theta \right)$ and ${\tilde{\ell }}_{\mathrm{MCAI}}\left(\theta \right)$ be the corresponding numerically evaluated approximations. Each approximation has its own maximizer, ${\hat{\theta }}_{\mathrm{GH}}=\arg\max{\tilde{\ell }}_{\mathrm{GH}}\left(\theta \right)$ and ${\hat{\theta }}_{\mathrm{MCAI}}=\arg\max{\tilde{\ell }}_{\mathrm{MCAI}}\left(\theta \right)$. By construction, ${\tilde{\ell }}_{\mathrm{GH}}\left({\hat{\theta }}_{\mathrm{GH}}\right)\geq {\tilde{\ell }}_{\mathrm{GH}}\left(\theta \right)$ and ${\tilde{\ell }}_{\mathrm{MCAI}}\left({\hat{\theta }}_{\mathrm{MCAI}}\right)\geq {\tilde{\ell }}_{\mathrm{MCAI}}\left(\theta \right)$ for all θ, including the simulation truth. The key empirical finding is that ${\hat{\theta }}_{\mathrm{GH}}$ and ${\hat{\theta }}_{\mathrm{MCAI}}$ can differ meaningfully in $\left(\hat{\sigma },\hat{\tau }\right)$ while agreeing closely on $\hat{S}=\sqrt{{\hat{\sigma }}^{2}+{\hat{\tau }}^{2}}$, indicating that approximation error influences which point on the ridge is selected. This clarifies why the MLE can be theoretically optimal *for the exact likelihood* yet practically biased when the likelihood must be approximated in a weakly identified regime.

**Why GLMM and Bayesian inference can be more robust under the same ridge**. The superior behavior of GLMM and Bayesian estimators in high‐r scenarios is consistent with the same ridge‐based theory: these methods stabilize variance decomposition in ways that are typically less sensitive to deterministic quadrature error. The probit GLMM fit (e.g., via Laplace approximation around the conditional mode) performs a mode‐centered integration that couples the random‐effect structure with the observed binomial counts, which tends to regularize variance components in finite samples. This does not remove the ridge in principle, but it can reduce the extent to which small numerical perturbations displace the optimizer along the ridge, yielding more stable $\left(\hat{\sigma },\hat{\tau }\right)$ across designs. Bayesian inference adds regularization through the prior, converting a ridge‐like likelihood into a posterior that is better behaved for variance decomposition and providing uncertainty quantification that directly reflects weak identification. This robustness is not automatic: when r is extremely small, the posterior can exhibit funnel geometry and require careful parameterization and prior choice to avoid poor mixing. The overarching message is that GLMM and Bayesian approaches can outperform direct marginal‐likelihood maximization in regimes where (i) (σ,τ) are weakly identified by the threshold summaries and (ii) likelihood evaluation is necessarily approximate.

**Practical implications**. Study count is an additional practical determinant of reliability. The primary simulation grid with I=20 and I=50 was intended to represent mature‐evidence settings, whereas the separate stress test mapped performance under joint sparse evidence. In the stress‐test design, which combined I=5 or 10 studies with $n_{i}∼\mathrm{Uniform}\left(10,50\right)$ and 5–10 thresholds per study, I=10 with low‐to‐moderate heterogeneity could still provide useful inference on $\mu_{0}$ and sometimes σ. However, τ was substantially less precise, and at I=5 or under strong heterogeneity the decomposition of total variation into $\sigma^{2}$ and $\tau^{2}$ was not reliable. Therefore, when the literature contains only a few studies, the framework is more appropriately used to generate plausible ranges for sensitivity analyses in future‐study planning than to provide a single confirmatory plug‐in estimate of the separate variance components. Method selection should be guided jointly by the available threshold information and the anticipated heterogeneity. With only one threshold per study, the data primarily identify the combined scale S and do not support reliable separation of σ and τ, so reporting and interpreting S (and $\mu_{0}$) is typically more defensible than emphasizing variance components. With multiple thresholds per study, separation becomes feasible, but high heterogeneity can still yield ridge‐like likelihoods that make pure likelihood maximization sensitive to integration error; in such settings, GLMM or Bayesian approaches with appropriate regularization are often more reliable in practice. Conversely, when heterogeneity is weak and sample sizes are moderate to large, simpler probit GLM formulations remain attractive due to their stability and minimal computational overhead.

**Limitations and future directions**. Several limitations merit attention. First, the framework assumes normal latent outcomes and normally distributed random study effects; heavy tails or systematic departures from normality may alter both identifiability and numerical behavior. Second, inference depends on accurate reporting of thresholds and sample sizes; rounding, coarse cut‐point reporting, or extreme‐tail thresholds can exacerbate weak identification and amplify numerical sensitivity. Third, while MCAI improves integration robustness relative to fixed‐node GH in some regimes, it cannot resolve weak identification and may remain sensitive when the ridge is pronounced. Future work will explore parameterizations that directly model (S,r), adaptive strategies that explicitly target ridge‐stable objectives, and extensions to alternative links or non‐Gaussian random effects. Incorporating study‐level covariates and covariate‐dependent thresholds is also a natural next step for applied meta‐analytic settings.

## 6 Conclusion

We developed and evaluated inference procedures for recovering latent normal distribution parameters from threshold‐type summaries under binomial‐probit models. In fixed‐mean designs ($\tau^{2}=0$), the binomial‐likelihood MLE and the probit GLM yield nearly identical inference for $\left(\mu_{0},\sigma \right)$, reflecting a well‐conditioned likelihood surface and the close relationship between probit regression fitting and likelihood maximization under correct specification.

In random‐mean designs with study effects $\mu_{i}∼N\left(\mu_{0},\tau^{2}\right)$, what can be learned from threshold summaries depends critically on the number and placement of cut points within each study. With only one threshold per study, the marginal model primarily identifies the combined scale $S=\sqrt{\sigma^{2}+\tau^{2}}$ and does not support reliable separation of σ and τ. With multiple thresholds per study, the additional within‐study information can render (σ,τ) identifiable, although variance‐component estimation may remain practically sensitive in high‐heterogeneity regimes where the likelihood is weakly informative about the σ–τ decomposition.

Across the hierarchical estimators we studied, differences in performance are driven mainly by how the marginal likelihood is evaluated and stabilized: GH MLE maximizes a quadrature‐approximated likelihood and can become sensitive when weak identification amplifies integration error, whereas GLMM (Laplace‐type) and Bayesian approaches often provide more stable variance decomposition through mode‐based regularization or prior regularization, respectively. These results provide guidance for applied analyses of privacy‐restricted or aggregated threshold data, clarifying when inference should focus on $\left(\mu_{0},S\right)$ and when richer threshold information supports estimation of $\left(\mu_{0},\sigma ,\tau \right)$.

## Author Contributions

**Xuekui Zhang:** conceptualization, funding acquisition, supervision, methodology, writing – review and editing. **Zhaoze Liu:** investigation, formal analysis, methodology, writing – original draft. **Longwen Shang:** data curation (UK BioBank), writing – review and editing. **Mary Lesperance:** writing – review and editing, supervision. **Shuiqing Zhou:** writing – review and editing.

## Conflicts of Interest

The authors declare no conflicts of interest.

## Open Research Badges

This article has earned an Open Data badge for making publicly available the digitally‐shareable data necessary to reproduce the reported results. The data is available in the Supporting Information section.

This article has earned an open data badge “**Reproducible Research**” for making publicly available the code necessary to reproduce the reported results. The results reported in this article could fully be reproduced.

## Supporting information


**Supporting File 1:** bimj70173‐sup‐0001‐SuppMat.pdf.

**Supporting File 2:** bimj70173‐sup‐0002‐Data.zip.

## Data Availability

The code used for the analysis has been uploaded to GitHub: https://github.com/zhaozeliu/Estimation‐of‐Underlying‐Normal‐Distribution‐Parameters‐from‐Dichotomized‐Data. Real data used in this work can be accessed from UK Biobank https://www.ukbiobank.ac.uk/. The R package bin2norm has already been submitted to R CRAN.
