# Supplementary material for: Estimation of Underlying Normal Distribution Parameters From Dichotomized Data
Source: Biom J. 2026 Sep 15;68(5):e70173. doi: 10.1002/bimj.70173 (PMC13576801; doi:10.1002/bimj.70173)
Supplement: Supplementary file 1 [file BIMJ-68-e70173-s001.pdf]

Received: Added at production | Revised: Added at production | Accepted: Added at production

DOI: xxx/xxxx

ARTICLE TYPE

Supplementary Document – Estimation of Underlying Normal Distribution Parameters from Dichotomized Data

Zhaoze Liu[1] | Longwen Shang[1] | Mary Lesperance[1] | Shuiqing Zhou[2] | Xuekui Zhang*[1,2]

[1]Department of Mathematics and Statistics, University of Victoria, British Columbia, Canada

[2]Innovation Research Institute of Zhejiang University of Technology, Shengzhou, China

Correspondence

*Correspondence to: Xuekui Zhang, Department of Mathematics and Statistics, University of Victoria, BC, Canada.
Email: xuekui@uvic.ca

KEYWORDS

hierarchical model, dichotomized data, MLE, GLM, GLMM, Bayesian MCMC

 © 2023 Copyright Holder Name

1 | MARGINAL LIKELIHOOD EVALUATION AND COMPARISON OF GH VS. MODE-CENTERED ADAPTIVE INTEGRATION

1.1 | Model and data structure

Consider study $i = 1, \ldots, I$. Let n_i denote the sample size and let

$$c_{i1} < c_{i2} < \cdots < c_{im_i}$$

be a set of ordered thresholds. Let Y_{ij} denote the latent continuous outcome for subject j in study i. We assume the random-mean probit model

$$Y_{ij} \mid \mu_i \sim N(\mu_i, \sigma^2), \qquad \mu_i \sim N(\mu_0, \tau^2),$$

where μ_i is a study-specific random mean.

Define the right-tail probabilities

$$p_{ik}(\mu_i) = \Pr(Y_{ij} > c_{ik} \mid \mu_i) = \Phi\left(\frac{\mu_i - c_{ik}}{\sigma}\right), \quad k = 1, \ldots, m_i.$$

1.2 | Multinomial representation induced by thresholds

The thresholds partition the real line into $m_i + 1$ disjoint intervals:

$$(-\infty, c_{i1}],\ (c_{i1}, c_{i2}],\ \ldots,\ (c_{i,m_i-1}, c_{im_i}],\ (c_{im_i}, \infty).$$

Define the corresponding interval probabilities

$$\begin{aligned}
\pi_{i1}(\mu_i) &= 1 - p_{i1}(\mu_i), \\
\pi_{ik}(\mu_i) &= p_{i,k-1}(\mu_i) - p_{ik}(\mu_i), \quad k = 2, \ldots, m_i, \\
\pi_{i,m_i+1}(\mu_i) &= p_{im_i}(\mu_i).
\end{aligned}$$

Let

$$\mathbf{K}_i = (K_{i1}, \ldots, K_{i,m_i+1})$$

denote the counts of observations falling into each interval, satisfying $\sum_{k=1}^{m_i+1} K_{ik} = n_i$. Conditional on μ_i,

$$\mathbf{K}_i \mid \mu_i \sim \text{Multinomial}\big(n_i; \pi_{i1}(\mu_i), \ldots, \pi_{i,m_i+1}(\mu_i)\big).$$

The conditional log-likelihood is

$$\ell_i(\mu_i) = \log \Pr(\mathbf{K}_i \mid \mu_i) = \log \frac{n_i!}{\prod_k K_{ik}!} + \sum_{k=1}^{m_i+1} K_{ik} \log \pi_{ik}(\mu_i).$$

1.3 | Marginal likelihood (non-analytic integral)

The marginal likelihood for study i is obtained by integrating out the random mean μ_i:

$$L_i(\theta) = \int_{-\infty}^{\infty} \exp\{\ell_i(\mu)\}\ \phi\left(\frac{\mu - \mu_0}{\tau}\right) \frac{1}{\tau}\, d\mu, \qquad \theta = (\mu_0, \sigma, \tau).$$

Because $\pi_{ik}(\mu)$ involves probit functions and appears inside the log-multinomial likelihood, this integral has no closed-form expression and must be evaluated numerically.

1.4 | Standardized integral form

Apply the change of variables

$$\mu = \mu_0 + \sqrt{2}\,\tau x.$$

Then the marginal likelihood becomes

$$L_i(\theta) = \frac{1}{\sqrt{\pi}} \int_{-\infty}^{\infty} \exp\Big\{ \ell_i(\mu_0 + \sqrt{2}\tau x) - x^2 \Big\}\, dx.$$

Define

$$h_i(x) = \ell_i(\mu_0 + \sqrt{2}\tau x) - x^2.$$

Thus,

$$\log L_i(\theta) = -\tfrac{1}{2}\log \pi + \log\left(\int_{-\infty}^{\infty} e^{h_i(x)}\, dx \right).$$

1.5 | Gauss–Hermite quadrature (fixed-node approximation)

Gauss–Hermite (GH) quadrature approximates integrals of the form

$$\int_{-\infty}^{\infty} e^{-x^2} f(x)\, dx \approx \sum_{g=1}^{G} w_g f(x_g),$$

where $\{x_g, w_g\}$ are fixed quadrature nodes and weights.

Applying GH quadrature to the marginal likelihood yields

$$\int_{-\infty}^{\infty} e^{h_i(x)}\, dx = \int e^{-x^2} \exp\{\ell_i(\mu_0 + \sqrt{2}\tau x)\}\, dx \approx \sum_{g=1}^{G} w_g \exp\{\ell_i(\mu_0 + \sqrt{2}\tau x_g)\}.$$

Hence,

$$\log L_i^{\mathrm{GH}}(\theta) = -\tfrac{1}{2}\log \pi + \log\left(\sum_{g=1}^{G} w_g \exp\{\ell_i(\mu_0 + \sqrt{2}\tau x_g)\} \right).$$

The GH-based marginal log-likelihood is therefore a *fixed-node numerical approximation* to the true marginal likelihood.

1.6 | Mode-centered adaptive integration (what it is, exactly)

The integrand $e^{h_i(x)}$ can be highly concentrated (sharp) when n_i is large or when multiple thresholds are used. To improve numerical accuracy, we evaluate the *same* marginal likelihood integral using a mode-centered adaptive integration strategy. Importantly, this procedure does *not* modify the statistical model; it only changes how the non-analytic integral is computed.

1.6.0.1 | *Step 1: Locate the mode of $h_i(x)$.*

Define the mode

$$x_i^* = \arg\max_{x \in \mathbb{R}} h_i(x), \qquad h_i^* = h_i(x_i^*).$$

In implementation, x_i^* is obtained by a one-dimensional maximization of $h_i(x)$ over a sufficiently wide interval (e.g., $[-B, B]$ with $B > 0$ large enough to contain the dominant mass).

1.6.0.2 | *Step 2: Estimate local curvature and the effective width.*

Assume h_i is twice differentiable in a neighborhood of x_i^* with $h_i''(x_i^*) < 0$. A numerical second derivative is used:

$$h_i''(x_i^*) \approx \frac{h_i(x_i^* + \varepsilon) - 2h_i(x_i^*) + h_i(x_i^* - \varepsilon)}{\varepsilon^2}.$$

Define the local scale (effective peak width)

$$s_i = \frac{1}{\sqrt{-h_i''(x_i^*)}}.$$

1.6.0.3 | *Step 3: Choose an adaptive integration domain.*
We integrate on the data- and parameter-adaptive interval

$$[L_i, U_i] = [x_i^* - Ks_i,\ x_i^* + Ks_i],$$

where $K > 0$ controls how many local standard-widths are included. Lower and upper bounds on Ks_i can be imposed for numerical stability in extreme cases (e.g., when curvature estimation is unreliable).

1.6.0.4 | *Step 4: Center the integrand at the mode for numerical stability.*
Using the identity

$$\int_{L_i}^{U_i} e^{h_i(x)}\, dx = e^{h_i^*} \int_{L_i}^{U_i} e^{h_i(x)-h_i^*}\, dx,$$

the centered integrand satisfies

$$0 \le e^{h_i(x)-h_i^*} \le 1,$$

which prevents overflow/underflow when $\ell_i(\cdot)$ is large in magnitude.

1.6.0.5 | *Step 5: Evaluate the integral with an adaptive quadrature routine.*
The remaining integral $\int_{L_i}^{U_i} e^{h_i(x)-h_i^*}\, dx$ is evaluated by a general-purpose adaptive numerical integrator (e.g., Gauss–Kronrod as implemented in `integrate()`), to specified absolute/relative tolerances.

Therefore, the resulting adaptive marginal log-likelihood is

$$\log L_i^{\text{adapt}}(\theta; K) = -\tfrac{1}{2}\log\pi + h_i^* + \log\left(\int_{L_i}^{U_i} e^{h_i(x)-h_i^*}\, dx\right), \quad [L_i, U_i] = [x_i^* - Ks_i,\ x_i^* + Ks_i].$$

The total log-likelihood is $\log L(\theta) = \sum_{i=1}^{I} \log L_i(\theta)$, and the negative log-likelihood is minimized to obtain the MLE.

1.7 | Why the mode-centered adaptive integration can be more accurate than GH

Both GH quadrature and mode-centered adaptive integration approximate the same non-analytic marginal integral

$$\int_{-\infty}^{\infty} e^{h_i(x)}\, dx.$$

However, their numerical behavior differs in a way that can be characterized mathematically.

1.7.1 | Local sharpness and concentration of the integrand

Let x_i^* be the maximizer of $h_i(x)$ and suppose $h_i''(x_i^*) < 0$. A second-order Taylor expansion around the mode gives

$$h_i(x) \approx h_i^* - \frac{(x - x_i^*)^2}{2s_i^2}, \qquad s_i = \frac{1}{\sqrt{-h_i''(x_i^*)}}.$$

Thus the effective width of the integrand $e^{h_i(x)}$ is of order s_i. In large-sample or multi-threshold settings, $\ell_i(\mu)$ can be highly curved, and consequently s_i can be very small (the integrand becomes sharply peaked). In such cases, accurately evaluating the integral requires concentrating numerical effort on the neighborhood $x_i^* \pm O(s_i)$ that contains the dominant mass.

1.7.2 | Why fixed-node GH can underestimate sharply peaked integrals

GH quadrature uses a fixed set of nodes $\{x_g\}$ that does not adapt to either the location x_i^* or the width s_i. Let

$$\delta_i = \min_{1 \le g \le G} |x_g - x_i^*|$$

be the distance from the mode to the nearest quadrature node. Under the local Gaussian approximation above,

$$\frac{e^{h_i(x_i^* - \delta_i)}}{e^{h_i^*}} \approx \exp\left(-\frac{\delta_i^2}{2s_i^2}\right).$$

When s_i is small, even a modest node misalignment δ_i yields an exponentially small ratio, implying that the dominant contribution to the integral may be missed by the fixed node set. Consequently,

$$\sum_{g=1}^{G} w_g \exp\{\ell_i(\mu_0 + \sqrt{2}\tau x_g)\} \ll \int e^{-x^2} \exp\{\ell_i(\mu_0 + \sqrt{2}\tau x)\}\, dx,$$

so the GH approximation can systematically underestimate the marginal likelihood in sharply peaked or displaced-mode scenarios.

1.7.3 | Why mode-centering avoids missing the dominant mass

The adaptive approach explicitly locates the mode x_i^* and scales the integration domain according to the local width s_i. Therefore, the dominant mass is always centered within the integration domain, preventing the "missed-peak" phenomenon that can occur with fixed-node quadrature.

1.8 | The role of K and its impact on the approximation

The parameter K does not enter the statistical model. Instead, it controls the numerical truncation of the integration domain in the mode-centered adaptive approximation,

$$[L_i, U_i] = [x_i^* - Ks_i,\; x_i^* + Ks_i].$$

Hence K plays the same role as the number of quadrature points G in GH or the absolute/relative tolerances in an adaptive integrator: it is an *accuracy parameter* for numerical integration.

1.8.1 | Truncation error bound (explicit and interpretable)

Assume a concavity (Gaussian-dominance) condition of the form

$$h_i(x) \le h_i^* - \frac{(x - x_i^*)^2}{2s_i^2} \quad \text{for all } x \in \mathbb{R}.$$

Then the truncated tail mass satisfies

$$\int_{|x - x_i^*| > Ks_i} e^{h_i(x)}\, dx \le e^{h_i^*} \int_{|u| > K} e^{-u^2/2}\, s_i\, du = e^{h_i^*} s_i \sqrt{2\pi}\, 2\Phi(-K).$$

Under the same local-Gaussian approximation, the full integral is of order

$$\int_{-\infty}^{\infty} e^{h_i(x)}\, dx \approx e^{h_i^*} s_i \sqrt{2\pi}.$$

Therefore, the relative truncation error is bounded by

$$\frac{\int_{|x-x_i^*|>Ks_i} e^{h_i(x)}\,dx}{\int_{-\infty}^{\infty} e^{h_i(x)}\,dx} \lesssim 2\Phi(-K).$$

This bound depends only on K, is independent of (μ_0, σ, τ), and becomes negligible for moderate values of K (e.g., $K \geq 6$). Consequently, once K is sufficiently large, the adaptive likelihood and the resulting MLE are insensitive to further increases in K, indicating numerical convergence of the integral approximation.

1.8.2 | Interpretation of choosing K

Because K controls truncation accuracy, selecting a larger K reduces truncation error at the expense of additional computation. This is analogous to increasing the number of GH points G to improve accuracy. Supplementary Figure 1 evaluates this sensitivity over the 36 primary random-mean simulation scenarios. Within each simulation replicate, MCAI estimates obtained using $K = 4, 6, 8$, and 10 were compared with the estimate from the same generated dataset using $K = 12$, the largest integration width evaluated. The paired comparison removes ordinary simulation variation and directly measures the extent to which the numerical integration domain changes the resulting estimates. The maximum scenario-specific median paired differences decreased rapidly with increasing K and were numerically negligible for $K \geq 8$. These results support $K = 10$ as a conservative default for the reported MCAI analyses.

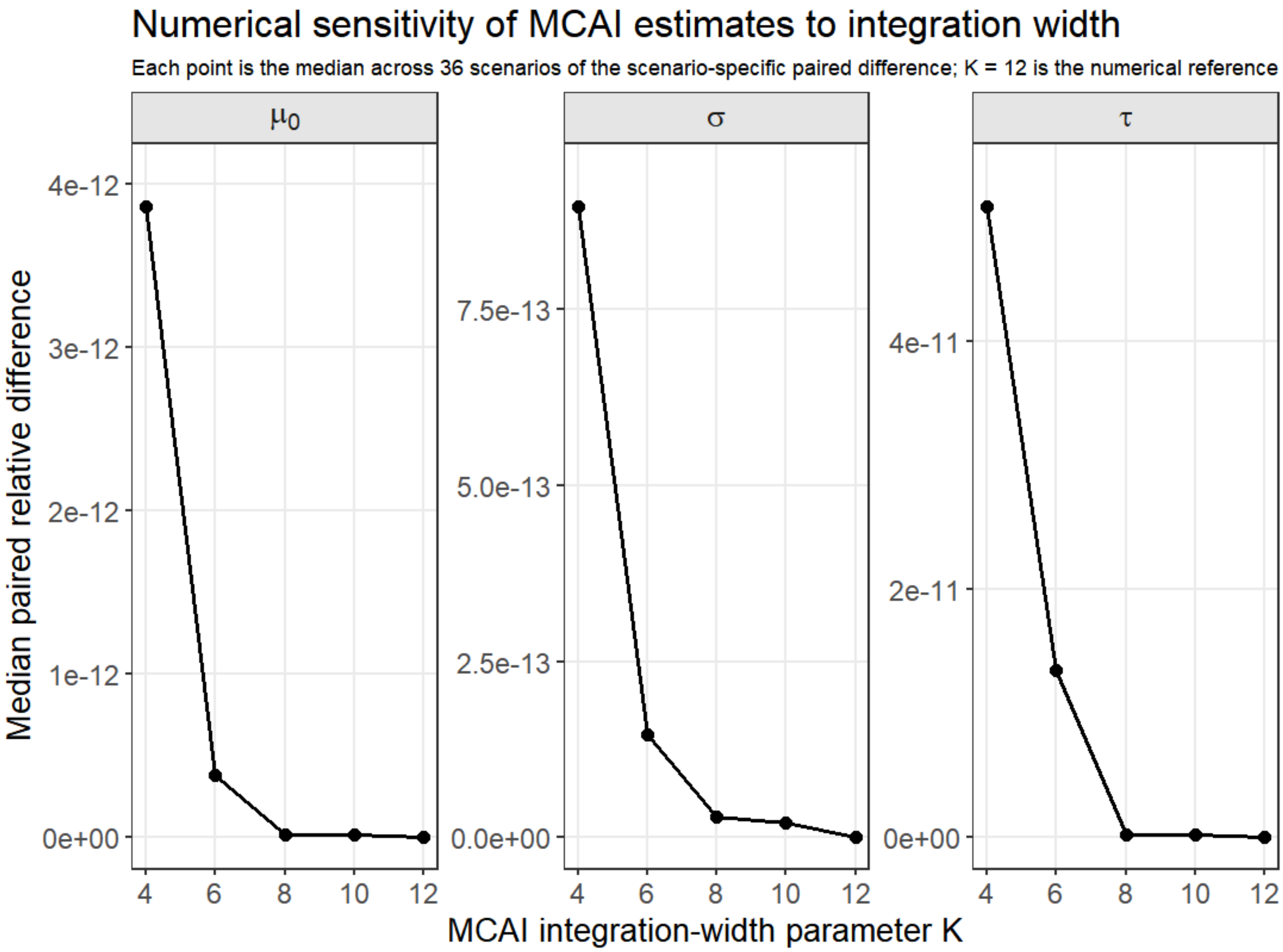

FIGURE S1 Numerical sensitivity of the mode-centered adaptive-integration estimates to the integration-width parameter K. Within each simulation replicate, estimates obtained using $K = 4, 6, 8$, and 10 were compared with the estimates from the same generated dataset using $K = 12$, the largest integration width investigated. For each parameter and scenario, paired relative differences were calculated as $\frac{|\hat{\theta}(K)-\hat{\theta}(12)|}{\theta_{\text{true}}}$, $\theta \in \mu_0, \sigma, \tau$. For each scenario, these paired differences were summarized by their median across valid paired fits. Each plotted point is the median across the 36 primary random-mean scenarios of these scenario-specific median differences. The value at $K = 12$ is zero by construction. Smaller values indicate lower sensitivity to the numerical integration width.

TABLE S1 Empirical bias of the estimated $\hat{\mu}_0$ under all 12 fixed-mean simulation scenarios for each combination of $I \in \{20, 50\}$, $\sigma \in \{0.1, 1.0\}$, and approach (A–C). Bias is computed as the average of $\hat{\mu}_0 - \mu_{\text{true}}$ across 1,000 simulation replicates, reported for both MLE and GLM.

I	σ	r	App.	**MLE**	**GLM**
20	0.1	0	A	-5.63×10^{-5}	-5.63×10^{-5}
20	0.1	0	B	-4.72×10^{-6}	-4.77×10^{-6}
20	0.1	0	C	-3.19×10^{-6}	-3.20×10^{-6}
50	0.1	0	A	-1.92×10^{-5}	-1.92×10^{-5}
50	0.1	0	B	-5.05×10^{-6}	-5.05×10^{-6}
50	0.1	0	C	2.41×10^{-6}	2.46×10^{-6}
20	1.0	0	A	-5.63×10^{-4}	-5.64×10^{-4}
20	1.0	0	B	-4.77×10^{-5}	-4.77×10^{-5}
20	1.0	0	C	-3.20×10^{-5}	-3.20×10^{-5}
50	1.0	0	A	-1.92×10^{-4}	-1.92×10^{-4}
50	1.0	0	B	-5.05×10^{-5}	-5.05×10^{-5}
50	1.0	0	C	2.46×10^{-5}	2.46×10^{-5}

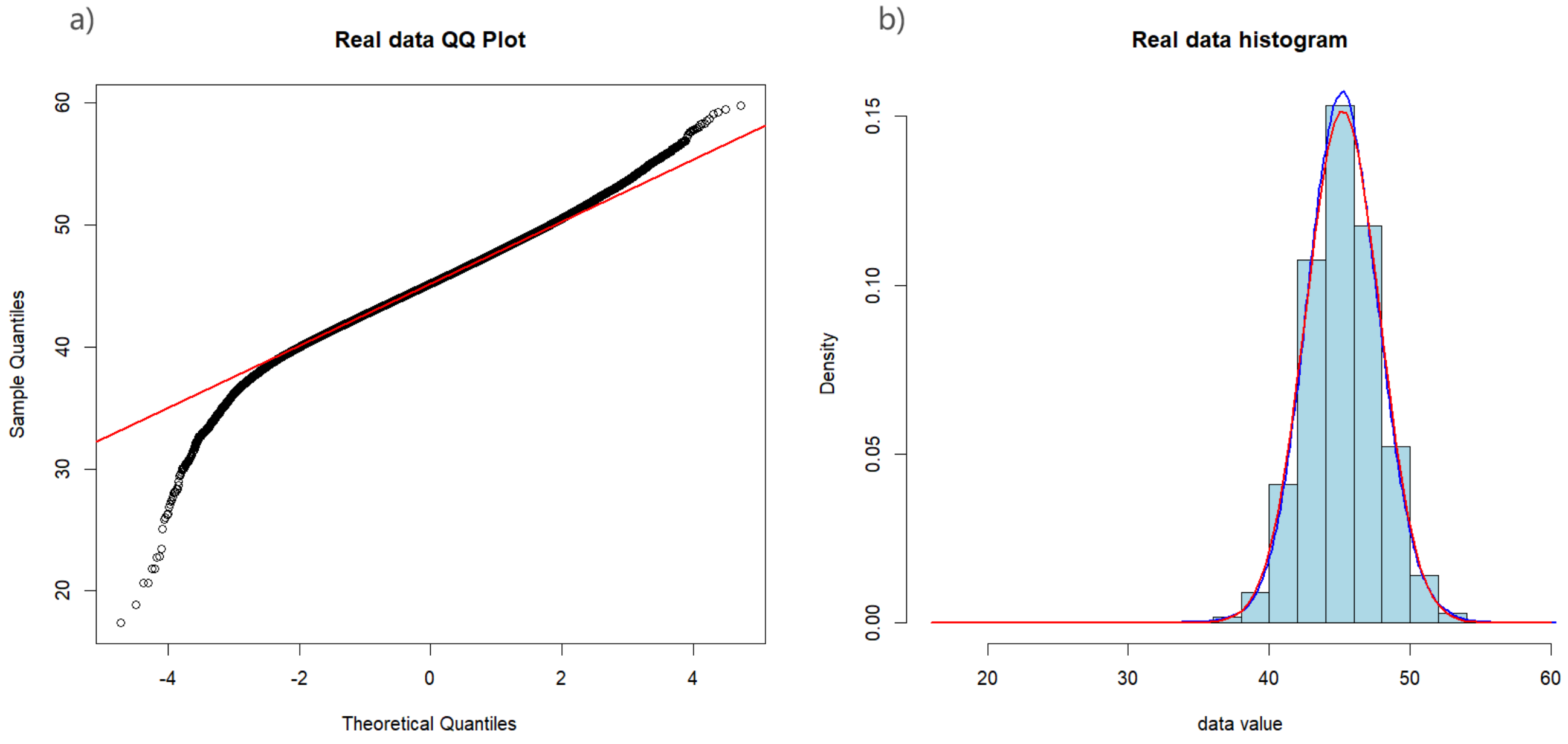

FIGURE S2 Diagnostic plots for the empirical distribution of the real data. (a) Normal Q–Q plot comparing the sample quantiles with theoretical normal quantiles. The points closely follow the reference line across most of the distribution, with only minor deviations in the tails. (b) Histogram of the observed data with an overlaid kernel density estimate (blue curve) and the fitted normal density (red curve). Both curves align closely over the bulk of the distribution. Overall, the diagnostics indicate that the data are approximately normally distributed, with no strong evidence against the normality assumption.

TABLE S2 Empirical variance of the estimated $\hat{\mu}_0$ under all 12 fixed-mean simulation scenarios for each combination of $I \in \{20, 50\}$, $\sigma \in \{0.1, 1.0\}$, and approach (A–C). Var($\hat{\mu}_0$) is sample variance of $\hat{\mu}_0$ across 1,000 simulation replicates, reported for both MLE and GLM.

I	σ	r	App.	**MLE**	**GLM**
20	0.1	0	A	1.03×10^{-5}	1.03×10^{-5}
20	0.1	0	B	1.36×10^{-7}	1.36×10^{-7}
20	0.1	0	C	7.29×10^{-8}	7.29×10^{-8}
50	0.1	0	A	4.67×10^{-6}	4.67×10^{-6}
50	0.1	0	B	4.72×10^{-8}	4.72×10^{-8}
50	0.1	0	C	2.80×10^{-8}	2.80×10^{-8}
20	1.0	0	A	1.03×10^{-3}	1.03×10^{-3}
20	1.0	0	B	1.36×10^{-5}	1.36×10^{-5}
20	1.0	0	C	7.29×10^{-6}	7.29×10^{-6}
50	1.0	0	A	4.67×10^{-4}	4.67×10^{-4}
50	1.0	0	B	4.72×10^{-6}	4.72×10^{-6}
50	1.0	0	C	2.80×10^{-6}	2.80×10^{-6}

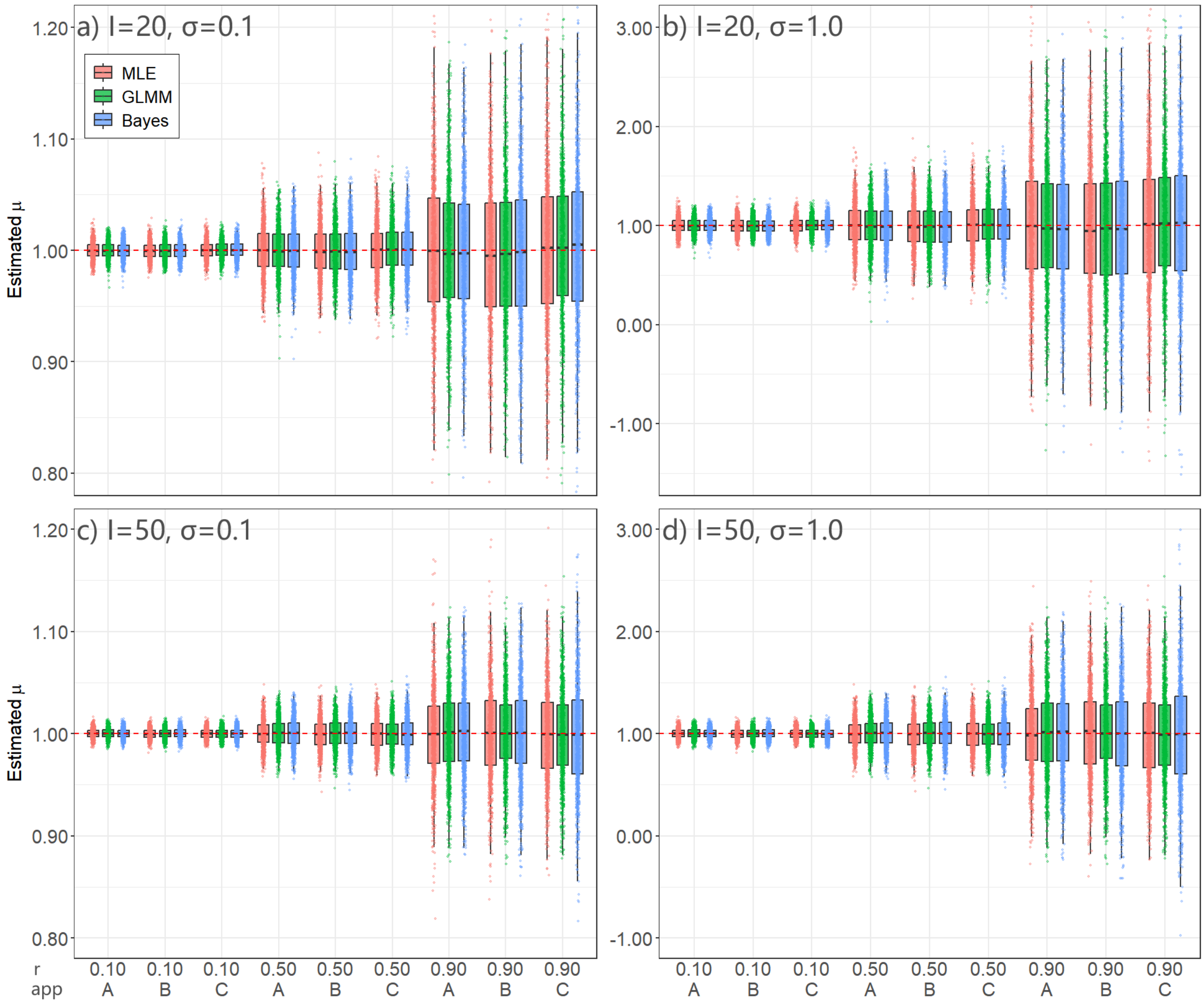

FIGURE S3 Boxplots summarize 1,000 simulation replicates for the integrated likelihood-based MLE using adaptive likelihood, the probit GLMM estimator, and the Bayesian MCMC approach across combinations of the number of studies ($I = 20, 50$), within-study standard deviation ($\sigma = 0.1, 1.0$), heterogeneity levels ($r = 0.1, 0.5, 0.9$), and study-size allocation designs (A–C). Panels (a)–(d) correspond to the four combinations of I and σ. The dashed horizontal line indicates the true value $\mu_0 = 1.0$.

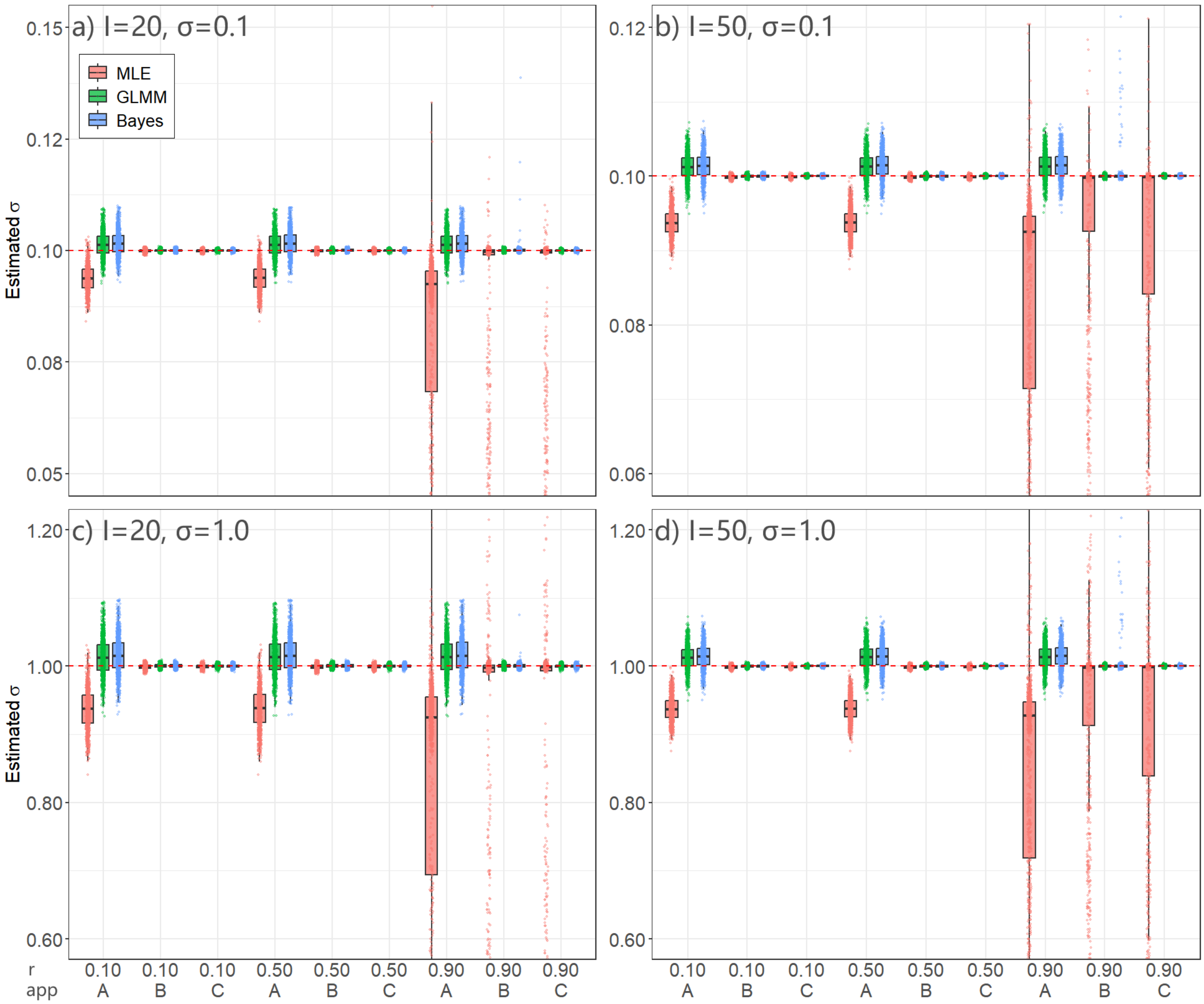

FIGURE S4 Sampling distributions of the estimated within-study standard deviation $\hat{\sigma}$ under the random-mean model. Boxplots summarize 1,000 simulation replicates for the likelihood-based integrated MLE using adaptive likelihood, the probit GLMM, and the Bayesian MCMC estimator across combinations of the number of studies ($I = 20, 50$), true within-study standard deviation ($\sigma = 0.1, 1.0$), heterogeneity levels ($r = 0.1, 0.5, 0.9$), and study-size allocation designs (A–C). Panels (a)–(b) correspond to $\sigma = 0.1$, and panels (c)–(d) to $\sigma = 1.0$. The dashed horizontal line denotes the true value of σ in each scenario.

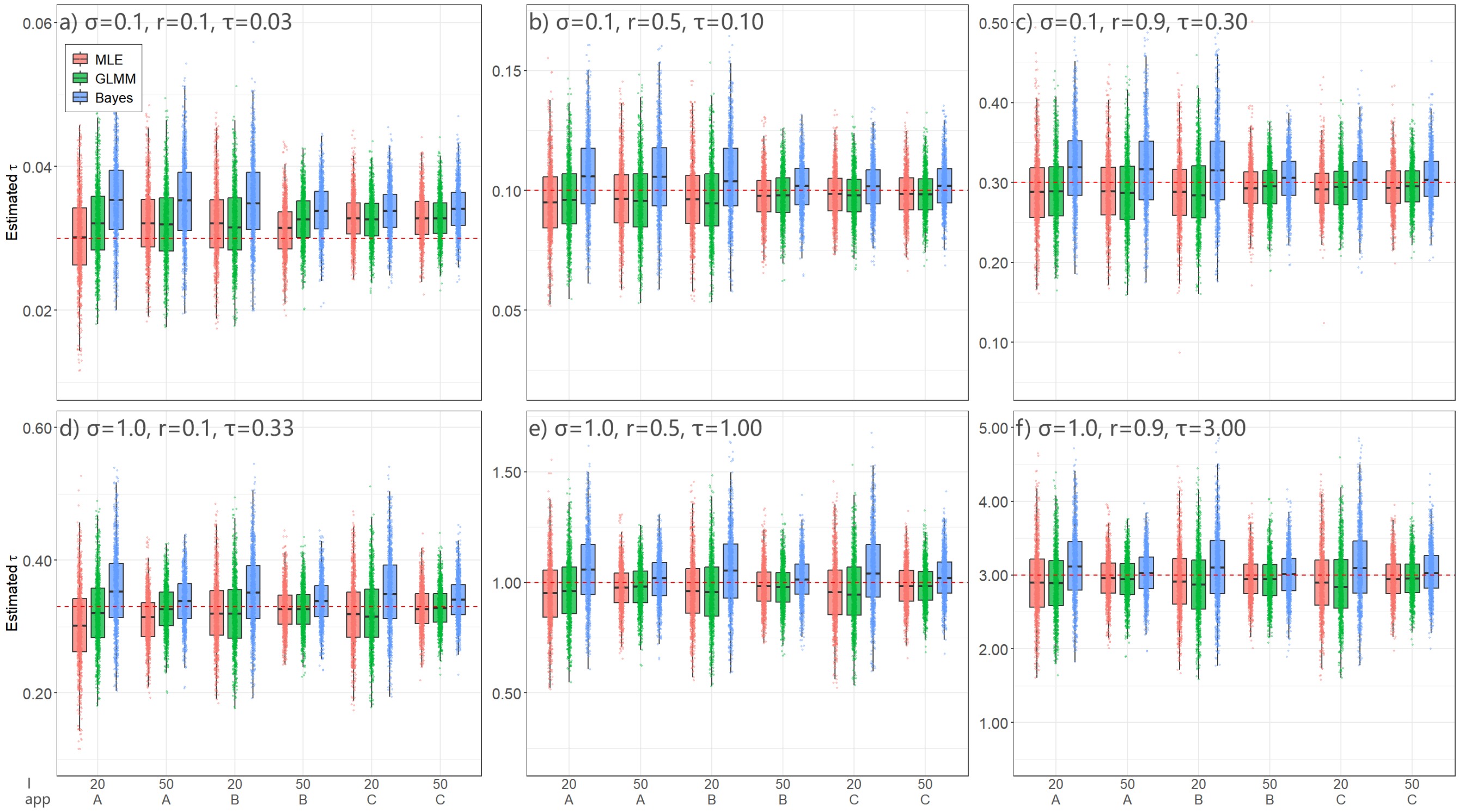

FIGURE S5 Sampling distributions of the estimated between-study standard deviation τ under the random-mean model. Boxplots summarize 1,000 simulation replicates for the integrated MLE using adaptive likelihood, probit-GLMM, and Bayesian MCMC estimators across combinations of number of studies ($I = 20, 50$), within-study standard deviation ($\sigma = 0.1, 1.0$), heterogeneity level ($r = 0.1, 0.5, 0.9$), and study-size allocation designs (A–C). The dashed horizontal line indicates the true value of τ implied by (σ, r).

TABLE S3 Empirical Mean squared error of the estimated $\hat{\mu}_0$ under all 12 fixed-mean simulation scenarios for each combination of $I \in \{20, 50\}$, $\sigma \in \{0.1, 1.0\}$, and approach (A–C). MSE computed as $\mathbb{E}\left[(\hat{\mu}_0 - \mu_{\text{true}})^2\right]$ across 1,000 simulation replicates, reported for both MLE and GLM.

I	σ	r	App.	MLE	GLM
20	0.1	0	A	1.03×10^{-5}	1.03×10^{-5}
20	0.1	0	B	1.35×10^{-7}	1.36×10^{-7}
20	0.1	0	C	7.29×10^{-8}	7.28×10^{-8}
50	0.1	0	A	4.66×10^{-6}	4.66×10^{-6}
50	0.1	0	B	4.72×10^{-8}	4.72×10^{-8}
50	0.1	0	C	2.80×10^{-8}	2.79×10^{-8}
20	1.0	0	A	1.03×10^{-3}	1.03×10^{-3}
20	1.0	0	B	1.36×10^{-5}	1.35×10^{-5}
20	1.0	0	C	7.28×10^{-6}	7.29×10^{-6}
50	1.0	0	A	4.66×10^{-4}	4.66×10^{-4}
50	1.0	0	B	4.71×10^{-6}	4.71×10^{-6}
50	1.0	0	C	2.80×10^{-6}	2.80×10^{-6}

TABLE S4 Empirical coverage probabilities of nominal 95% confidence intervals of the estimated $\hat{\mu}_0$ under all 12 fixed-mean simulation scenarios for each combination of $I \in \{20, 50\}$, $\sigma \in \{0.1, 1.0\}$, and approach (A–C). Coverage is defined as the proportion of 1,000 simulation replicates in which the interval contains the true value, reported for both MLE and GLM.

I	σ	r	App.	**MLE**	**GLM**
20	0.1	0	A	0.95	0.95
20	0.1	0	B	0.96	0.95
20	0.1	0	C	0.95	0.95
50	0.1	0	A	0.95	0.95
50	0.1	0	B	0.96	0.96
50	0.1	0	C	0.96	0.96
20	1.0	0	A	0.95	0.95
20	1.0	0	B	0.96	0.95
20	1.0	0	C	0.95	0.95
50	1.0	0	A	0.95	0.95
50	1.0	0	B	0.96	0.96
50	1.0	0	C	0.96	0.96

TABLE S5 Empirical bias of the estimated $\hat{\sigma}$ under all 12 fixed-mean simulation scenarios for each combination of $I \in \{20, 50\}$, $\sigma \in \{0.1, 1.0\}$, and approach (A–C). Bias is computed as the average of $\hat{\sigma} - \sigma_{\text{true}}$ across 1,000 simulation replicates, reported for both MLE and GLM.

I	σ	r	App.	MLE	GLM
20	0.1	0	A	3.98×10^{-4}	3.89×10^{-4}
20	0.1	0	B	6.97×10^{-5}	6.05×10^{-5}
20	0.1	0	C	-2.85×10^{-6}	-1.08×10^{-5}
50	0.1	0	A	1.32×10^{-4}	1.24×10^{-4}
50	0.1	0	B	4.09×10^{-5}	3.21×10^{-5}
50	0.1	0	C	2.13×10^{-6}	-5.79×10^{-6}
20	1.0	0	A	3.90×10^{-3}	3.89×10^{-3}
20	1.0	0	B	6.06×10^{-4}	6.05×10^{-4}
20	1.0	0	C	-1.07×10^{-4}	-1.08×10^{-4}
50	1.0	0	A	1.24×10^{-3}	1.24×10^{-3}
50	1.0	0	B	3.21×10^{-4}	3.21×10^{-4}
50	1.0	0	C	-5.71×10^{-5}	-5.79×10^{-5}

TABLE S6 Empirical variance of the estimated $\hat{\sigma}$ under all 12 fixed-mean simulation scenarios for each combination of $I \in \{20, 50\}$, $\sigma \in \{0.1, 1.0\}$, and approach (A–C). Var($\hat{\sigma}$) is sample variance of $\hat{\sigma}$ across 1,000 simulation replicates, reported for both MLE and GLM.

I	σ	r	App.	MLE	GLM
20	0.1	0	A	5.91×10^{-5}	5.91×10^{-5}
20	0.1	0	B	6.06×10^{-7}	6.06×10^{-7}
20	0.1	0	C	3.10×10^{-7}	3.10×10^{-7}
50	0.1	0	A	1.95×10^{-5}	1.95×10^{-5}
50	0.1	0	B	2.15×10^{-7}	2.15×10^{-7}
50	0.1	0	C	1.24×10^{-7}	1.24×10^{-7}
20	1.0	0	A	5.91×10^{-3}	5.91×10^{-3}
20	1.0	0	B	6.06×10^{-5}	6.06×10^{-5}
20	1.0	0	C	3.10×10^{-5}	3.10×10^{-5}
50	1.0	0	A	1.95×10^{-3}	1.95×10^{-3}
50	1.0	0	B	2.15×10^{-5}	2.15×10^{-5}
50	1.0	0	C	1.24×10^{-5}	1.24×10^{-5}

TABLE S7 Empirical Mean squared error of the estimated $\hat{\sigma}$ under all 12 fixed-mean simulation scenarios for each combination of $I \in \{20, 50\}$, $\sigma \in \{0.1, 1.0\}$, and approach (A–C). MSE computed as $\mathbb{E}\left[(\hat{\sigma} - \sigma_{\text{true}})^2\right]$ across 1,000 simulation replicates, reported for both MLE and GLM.

I	σ	r	App.	MLE	GLM
20	0.1	0	A	5.91×10^{-5}	5.92×10^{-5}
20	0.1	0	B	6.10×10^{-7}	6.09×10^{-7}
20	0.1	0	C	3.10×10^{-7}	3.10×10^{-7}
50	0.1	0	A	1.95×10^{-5}	1.95×10^{-5}
50	0.1	0	B	2.17×10^{-7}	2.16×10^{-7}
50	0.1	0	C	1.24×10^{-7}	1.24×10^{-7}
20	1.0	0	A	5.92×10^{-3}	5.92×10^{-3}
20	1.0	0	B	6.09×10^{-5}	6.09×10^{-5}
20	1.0	0	C	3.10×10^{-5}	3.10×10^{-5}
50	1.0	0	A	1.95×10^{-3}	1.95×10^{-3}
50	1.0	0	B	2.16×10^{-5}	2.16×10^{-5}
50	1.0	0	C	1.24×10^{-5}	1.24×10^{-5}

TABLE S8 Empirical coverage probabilities of nominal 95% confidence intervals of the estimated $\hat{\sigma}$ under all 12 fixed-mean simulation scenarios for each combination of $I \in \{20, 50\}$, $\sigma \in \{0.1, 1.0\}$, and approach (A–C). Coverage is defined as the proportion of 1,000 simulation replicates in which the interval contains the true value, reported for both MLE and GLM.

I	σ	r	App.	MLE	GLM
20	0.1	0	A	0.95	0.95
20	0.1	0	B	0.94	0.94
20	0.1	0	C	0.95	0.95
50	0.1	0	A	0.95	0.95
50	0.1	0	B	0.94	0.94
50	0.1	0	C	0.96	0.96
20	1.0	0	A	0.95	0.95
20	1.0	0	B	0.94	0.94
20	1.0	0	C	0.95	0.95
50	1.0	0	A	0.95	0.95
50	1.0	0	B	0.94	0.94
50	1.0	0	C	0.96	0.96

TABLE S9 Empirical bias of the estimated $\hat{\mu}_0$ under all 36 random-mean simulation scenarios for each combination of $I \in \{20, 50\}$, $\sigma \in \{0.1, 1.0\}$, $r \in \{0.1, 0.5, 0.9\}$, and approach (A–C). Bias is computed as the average of $\hat{\mu}_0 - \mu_{\text{true}}$ across 1,000 simulation replicates, reported for MLE, GLMM and MCMC.

I	σ	r	App.	**MLE**	**GLMM**	**MCMC**
20	0.1	0.1	A	-1.03×10^{-4}	-1.57×10^{-4}	-1.53×10^{-4}
20	0.1	0.1	B	-4.75×10^{-4}	-3.21×10^{-4}	1.04×10^{-2}
20	0.1	0.1	C	-2.70×10^{-4}	2.99×10^{-4}	2.59×10^{-4}
20	0.1	0.5	A	2.72×10^{-6}	-3.93×10^{-4}	-3.42×10^{-4}
20	0.1	0.5	B	-3.05×10^{-3}	-9.74×10^{-4}	-9.77×10^{-4}
20	0.1	0.5	C	-1.69×10^{-3}	9.02×10^{-4}	4.09×10^{-3}
20	0.1	0.9	A	8.87×10^{-4}	-1.09×10^{-3}	-1.32×10^{-3}
20	0.1	0.9	B	-1.14×10^{-2}	-2.93×10^{-3}	1.13×10^{-2}
20	0.1	0.9	C	1.99×10^{-3}	2.71×10^{-3}	3.66×10^{-3}
50	0.1	0.1	A	-4.09×10^{-5}	-4.66×10^{-5}	-5.67×10^{-5}
50	0.1	0.1	B	-1.69×10^{-4}	-1.03×10^{-4}	-1.18×10^{-4}
50	0.1	0.1	C	2.17×10^{-4}	2.11×10^{-4}	2.09×10^{-4}
50	0.1	0.5	A	-6.32×10^{-4}	5.88×10^{-4}	6.08×10^{-4}
50	0.1	0.5	B	-1.80×10^{-3}	-1.46×10^{-3}	-1.45×10^{-3}
50	0.1	0.5	C	-8.92×10^{-4}	-8.93×10^{-4}	-8.72×10^{-4}
50	0.1	0.9	A	-7.44×10^{-4}	5.70×10^{-3}	5.65×10^{-3}
50	0.1	0.9	B	-1.48×10^{-2}	-2.22×10^{-3}	-4.44×10^{-3}
50	0.1	0.9	C	-3.65×10^{-3}	-6.69×10^{-3}	-1.33×10^{-2}
20	1.0	0.1	A	-1.40×10^{-3}	-1.57×10^{-3}	-1.44×10^{-3}
20	1.0	0.1	B	-1.44×10^{-3}	-1.10×10^{-3}	-1.13×10^{-3}
20	1.0	0.1	C	-6.05×10^{-4}	-8.19×10^{-4}	-7.63×10^{-4}
20	1.0	0.5	A	-2.51×10^{-3}	-2.16×10^{-3}	-2.18×10^{-3}
20	1.0	0.5	B	-1.58×10^{-2}	-3.14×10^{-3}	-2.73×10^{-3}
20	1.0	0.5	C	-2.07×10^{-3}	1.58×10^{-3}	2.63×10^{-3}
20	1.0	0.9	A	1.52×10^{-2}	-9.99×10^{-3}	-1.40×10^{-2}
20	1.0	0.9	B	-6.93×10^{-3}	-3.53×10^{-2}	8.75×10^{-2}
20	1.0	0.9	C	-1.48×10^{-2}	1.72×10^{-2}	1.75×10^{-2}
50	1.0	0.1	A	5.40×10^{-4}	4.81×10^{-4}	4.77×10^{-4}
50	1.0	0.1	B	-1.06×10^{-4}	-1.46×10^{-5}	-1.53×10^{-5}
50	1.0	0.1	C	-5.07×10^{-4}	-4.40×10^{-4}	-4.41×10^{-4}
50	1.0	0.5	A	1.34×10^{-3}	1.21×10^{-3}	1.20×10^{-3}
50	1.0	0.5	B	-5.63×10^{-3}	-1.92×10^{-3}	-1.83×10^{-3}
50	1.0	0.5	C	1.14×10^{-2}	5.65×10^{-3}	5.59×10^{-3}
50	1.0	0.9	A	7.00×10^{-3}	1.49×10^{-2}	1.49×10^{-2}
50	1.0	0.9	B	-3.01×10^{-2}	6.96×10^{-3}	-8.68×10^{-3}
50	1.0	0.9	C	-3.29×10^{-2}	-5.71×10^{-3}	-1.79×10^{-2}

TABLE S10 Empirical variance of the estimated $\hat{\mu}_0$ under all 36 random-mean simulation scenarios for each combination of $I \in \{20, 50\}$, $\sigma \in \{0.1, 1.0\}$, $r \in \{0.1, 0.5, 0.9\}$, and approach (A–C). Var($\hat{\mu}_0$) is sample variance of $\hat{\mu}_0$ across 1,000 simulation replicates, reported for MLE, GLMM and MCMC.

I	σ	r	App.	**MLE**	**GLMM**	**MCMC**
20	0.1	0.1	A	6.49×10^{-5}	5.52×10^{-5}	5.57×10^{-5}
20	0.1	0.1	B	2.17×10^{-4}	5.62×10^{-5}	0.114
20	0.1	0.1	C	2.60×10^{-4}	5.60×10^{-5}	5.73×10^{-5}
20	0.1	0.5	A	8.61×10^{-4}	4.73×10^{-4}	4.76×10^{-4}
20	0.1	0.5	B	8.03×10^{-3}	5.05×10^{-4}	5.20×10^{-4}
20	0.1	0.5	C	6.54×10^{-3}	5.05×10^{-4}	0.0108
20	0.1	0.9	A	0.0113	4.22×10^{-3}	4.29×10^{-3}
20	0.1	0.9	B	0.0500	4.55×10^{-3}	0.138
20	0.1	0.9	C	0.0459	4.54×10^{-3}	5.40×10^{-3}
20	1.0	0.1	A	6.46×10^{-3}	5.52×10^{-3}	5.52×10^{-3}
20	1.0	0.1	B	0.0117	5.62×10^{-3}	5.57×10^{-3}
20	1.0	0.1	C	9.26×10^{-3}	5.60×10^{-3}	5.73×10^{-3}
20	1.0	0.5	A	0.0749	0.0473	0.0476
20	1.0	0.5	B	0.241	0.0505	0.0520
20	1.0	0.5	C	0.185	0.0505	0.0108
20	1.0	0.9	A	0.360	0.422	0.429
20	1.0	0.9	B	0.250	0.455	0.138
20	1.0	0.9	C	0.105	0.454	0.318
50	0.1	0.1	A	1.96×10^{-5}	2.34×10^{-5}	2.40×10^{-5}
50	0.1	0.1	B	2.12×10^{-5}	2.21×10^{-5}	2.31×10^{-5}
50	0.1	0.1	C	2.10×10^{-5}	2.20×10^{-5}	2.31×10^{-5}
50	0.1	0.5	A	1.32×10^{-4}	1.99×10^{-4}	2.17×10^{-4}
50	0.1	0.5	B	3.00×10^{-4}	2.00×10^{-4}	2.17×10^{-4}
50	0.1	0.5	C	2.95×10^{-4}	1.99×10^{-4}	2.17×10^{-4}
50	0.1	0.9	A	1.09×10^{-3}	1.79×10^{-3}	1.94×10^{-3}
50	0.1	0.9	B	5.49×10^{-3}	1.81×10^{-3}	0.0608
50	0.1	0.9	C	5.33×10^{-3}	1.81×10^{-3}	0.113
50	1.0	0.1	A	1.96×10^{-3}	2.34×10^{-3}	2.40×10^{-3}
50	1.0	0.1	B	2.12×10^{-3}	2.21×10^{-3}	2.31×10^{-3}
50	1.0	0.1	C	2.10×10^{-3}	2.20×10^{-3}	2.31×10^{-3}
50	1.0	0.5	A	0.0132	0.0199	0.0217
50	1.0	0.5	B	0.0300	0.0200	0.0217
50	1.0	0.5	C	0.0295	0.0199	0.0217
50	1.0	0.9	A	0.109	0.179	0.194
50	1.0	0.9	B	0.549	0.181	0.0608
50	1.0	0.9	C	0.533	0.181	0.113

TABLE S11 Empirical Mean squared error of the estimated $\hat{\mu}_0$ under all 36 random-mean simulation scenarios for each combination of $I \in \{20, 50\}$, $\sigma \in \{0.1, 1.0\}$, $r \in \{0.1, 0.5, 0.9\}$, and approach (A–C). MSE computed as $\mathbb{E}\left[(\hat{\mu}_0 - \mu_{\text{true}})^2\right]$ across 1,000 simulation replicates, reported for MLE, GLMM and MCMC.

I	σ	r	App.	**MLE**	**GLMM**	**MCMC**
20	0.1	0.1	A	6.49×10^{-5}	5.52×10^{-5}	5.57×10^{-5}
20	0.1	0.1	B	2.17×10^{-4}	5.62×10^{-5}	0.114
20	0.1	0.1	C	2.60×10^{-4}	5.61×10^{-5}	5.73×10^{-5}
20	0.1	0.5	A	8.60×10^{-4}	4.73×10^{-4}	4.76×10^{-4}
20	0.1	0.5	B	8.03×10^{-3}	5.06×10^{-4}	5.21×10^{-4}
20	0.1	0.5	C	6.54×10^{-3}	5.05×10^{-4}	0.0108
20	0.1	0.9	A	0.0112	4.22×10^{-3}	4.29×10^{-3}
20	0.1	0.9	B	0.0501	4.55×10^{-3}	0.138
20	0.1	0.9	C	0.0458	4.55×10^{-3}	5.41×10^{-3}
20	1.0	0.1	A	6.46×10^{-3}	5.52×10^{-3}	5.52×10^{-3}
20	1.0	0.1	B	0.0117	5.62×10^{-3}	5.57×10^{-3}
20	1.0	0.1	C	9.26×10^{-3}	5.61×10^{-3}	5.73×10^{-3}
20	1.0	0.5	A	0.0749	0.0473	0.0476
20	1.0	0.5	B	0.241	0.0506	0.0521
20	1.0	0.5	C	0.185	0.0505	0.0108
20	1.0	0.9	A	0.360	0.422	0.429
20	1.0	0.9	B	0.250	0.455	0.138
20	1.0	0.9	C	0.105	0.454	0.318
50	0.1	0.1	A	1.96×10^{-5}	2.35×10^{-5}	2.40×10^{-5}
50	0.1	0.1	B	2.12×10^{-5}	2.22×10^{-5}	2.33×10^{-5}
50	0.1	0.1	C	2.10×10^{-5}	2.22×10^{-5}	2.33×10^{-5}
50	0.1	0.5	A	1.32×10^{-4}	1.99×10^{-4}	2.17×10^{-4}
50	0.1	0.5	B	3.00×10^{-4}	2.00×10^{-4}	2.17×10^{-4}
50	0.1	0.5	C	2.95×10^{-4}	2.00×10^{-4}	2.17×10^{-4}
50	0.1	0.9	A	1.09×10^{-3}	1.79×10^{-3}	1.94×10^{-3}
50	0.1	0.9	B	5.49×10^{-3}	1.81×10^{-3}	0.0608
50	0.1	0.9	C	5.33×10^{-3}	1.81×10^{-3}	0.113
50	1.0	0.1	A	1.96×10^{-3}	2.35×10^{-3}	2.40×10^{-3}
50	1.0	0.1	B	2.12×10^{-3}	2.22×10^{-3}	2.33×10^{-3}
50	1.0	0.1	C	2.10×10^{-3}	2.22×10^{-3}	2.33×10^{-3}
50	1.0	0.5	A	0.0132	0.0200	0.0217
50	1.0	0.5	B	0.0300	0.0200	0.0217
50	1.0	0.5	C	0.0295	0.0200	0.0217
50	1.0	0.9	A	0.109	0.179	0.194
50	1.0	0.9	B	0.549	0.181	0.0608
50	1.0	0.9	C	0.533	0.181	0.113

TABLE S12 Empirical coverage probabilities of nominal 95% confidence intervals of the estimated $\hat{\mu}_0$ under all 36 random-mean simulation scenarios for each combination of $I \in \{20, 50\}$, $\sigma \in \{0.1, 1.0\}$, $r \in \{0.1, 0.5, 0.9\}$, and approach (A–C). Coverage is defined as the proportion of 1,000 simulation replicates in which the interval contains the true value, reported for MLE, GLMM and MCMC.

I	σ	r	App.	**MLE**	**GLMM**	**MCMC**
20	0.1	0.1	A	0.949	0.951	0.956
20	0.1	0.1	B	0.959	0.949	0.999
20	0.1	0.1	C	0.96	0.948	0.947
20	0.1	0.5	A	0.954	0.954	0.949
20	0.1	0.5	B	0.931	0.948	0.953
20	0.1	0.5	C	0.945	0.948	0.999
20	0.1	0.9	A	0.957	0.955	0.951
20	0.1	0.9	B	0.943	0.948	0.998
20	0.1	0.9	C	0.948	0.949	0.955
20	1.0	0.1	A	0.948	0.951	0.95
20	1.0	0.1	B	0.95	0.949	0.956
20	1.0	0.1	C	0.961	0.948	0.947
20	1.0	0.5	A	0.951	0.954	0.949
20	1.0	0.5	B	0.938	0.948	0.953
20	1.0	0.5	C	0.944	0.948	0.999
20	1.0	0.9	A	0.953	0.955	0.951
20	1.0	0.9	B	0.944	0.948	0.998
20	1.0	0.9	C	0.952	0.949	0.955
50	0.1	0.1	A	0.945	0.95	0.953
50	0.1	0.1	B	0.947	0.95	0.949
50	0.1	0.1	C	0.949	0.95	0.952
50	0.1	0.5	A	0.955	0.95	0.952
50	0.1	0.5	B	0.954	0.95	0.949
50	0.1	0.5	C	0.95	0.95	0.952
50	0.1	0.9	A	0.953	0.95	0.952
50	0.1	0.9	B	0.949	0.95	0.999
50	0.1	0.9	C	0.947	0.95	0.999
50	1.0	0.1	A	0.945	0.95	0.953
50	1.0	0.1	B	0.947	0.95	0.949
50	1.0	0.1	C	0.949	0.95	0.952
50	1.0	0.5	A	0.955	0.95	0.952
50	1.0	0.5	B	0.954	0.95	0.949
50	1.0	0.5	C	0.95	0.95	0.952
50	1.0	0.9	A	0.953	0.95	0.952
50	1.0	0.9	B	0.949	0.95	0.999
50	1.0	0.9	C	0.947	0.95	0.999

TABLE S13 Empirical bias of the estimated $\hat{\sigma}$ under all 36 random-mean simulation scenarios for each combination of $I \in \{20, 50\}$, $\sigma \in \{0.1, 1.0\}$, $r \in \{0.1, 0.5, 0.9\}$, and approach (A–C). Bias is computed as the average of $\hat{\sigma} - \sigma_{\text{true}}$ across 1,000 simulation replicates, reported for MLE, GLMM and MCMC.

I	σ	r	App.	**MLE**	**GLMM**	**MCMC**
20	0.1	0.1	A	-6.21×10^{-3}	1.34×10^{-3}	1.62×10^{-3}
20	0.1	0.1	B	2.71×10^{-4}	2.45×10^{-5}	2.72×10^{-5}
20	0.1	0.1	C	3.57×10^{-4}	1.25×10^{-7}	1.49×10^{-6}
20	0.1	0.5	A	-4.39×10^{-3}	1.40×10^{-3}	1.67×10^{-3}
20	0.1	0.5	B	2.76×10^{-3}	2.44×10^{-5}	2.76×10^{-5}
20	0.1	0.5	C	2.46×10^{-3}	1.24×10^{-7}	1.52×10^{-6}
20	0.1	0.9	A	1.62×10^{-2}	1.39×10^{-3}	1.67×10^{-3}
20	0.1	0.9	B	1.98×10^{-2}	2.44×10^{-5}	2.47×10^{-3}
20	0.1	0.9	C	1.96×10^{-2}	1.24×10^{-7}	1.44×10^{-6}
20	1.0	0.1	A	-6.22×10^{-2}	1.34×10^{-2}	1.62×10^{-2}
20	1.0	0.1	B	2.71×10^{-3}	2.45×10^{-4}	2.72×10^{-4}
20	1.0	0.1	C	3.57×10^{-3}	1.25×10^{-6}	1.49×10^{-5}
20	1.0	0.5	A	-4.39×10^{-2}	1.40×10^{-2}	1.67×10^{-2}
20	1.0	0.5	B	2.76×10^{-2}	2.44×10^{-4}	2.76×10^{-4}
20	1.0	0.5	C	2.46×10^{-2}	1.24×10^{-6}	1.52×10^{-5}
20	1.0	0.9	A	1.62×10^{-1}	1.39×10^{-2}	1.67×10^{-2}
20	1.0	0.9	B	1.98×10^{-1}	2.44×10^{-4}	2.47×10^{-2}
20	1.0	0.9	C	1.96×10^{-1}	1.24×10^{-6}	1.44×10^{-5}
50	0.1	0.1	A	-5.83×10^{-3}	1.22×10^{-3}	1.35×10^{-3}
50	0.1	0.1	B	1.04×10^{-4}	2.22×10^{-5}	2.28×10^{-5}
50	0.1	0.1	C	4.37×10^{-4}	-2.64×10^{-7}	2.53×10^{-6}
50	0.1	0.5	A	-4.22×10^{-3}	1.25×10^{-3}	1.39×10^{-3}
50	0.1	0.5	B	3.23×10^{-3}	2.22×10^{-5}	2.29×10^{-5}
50	0.1	0.5	C	2.33×10^{-3}	-2.64×10^{-7}	2.58×10^{-6}
50	0.1	0.9	A	3.21×10^{-2}	1.24×10^{-3}	1.38×10^{-3}
50	0.1	0.9	B	5.85×10^{-2}	2.22×10^{-5}	2.79×10^{-3}
50	0.1	0.9	C	5.80×10^{-2}	-2.64×10^{-7}	2.40×10^{-6}
50	1.0	0.1	A	-5.83×10^{-2}	1.22×10^{-2}	1.35×10^{-2}
50	1.0	0.1	B	1.04×10^{-3}	2.22×10^{-4}	2.28×10^{-4}
50	1.0	0.1	C	4.37×10^{-3}	-2.64×10^{-6}	2.53×10^{-5}
50	1.0	0.5	A	-4.22×10^{-2}	1.25×10^{-2}	1.39×10^{-2}
50	1.0	0.5	B	3.23×10^{-2}	2.22×10^{-4}	2.29×10^{-4}
50	1.0	0.5	C	2.33×10^{-2}	-2.64×10^{-6}	2.58×10^{-5}
50	1.0	0.9	A	3.21×10^{-1}	1.24×10^{-2}	1.38×10^{-2}
50	1.0	0.9	B	5.85×10^{-1}	2.22×10^{-4}	2.79×10^{-2}
50	1.0	0.9	C	5.80×10^{-1}	-2.64×10^{-6}	2.40×10^{-5}

TABLE S14 Empirical variance of the estimated $\hat{\sigma}$ under all 36 random-mean simulation scenarios for each combination of $I \in \{20, 50\}$, $\sigma \in \{0.1, 1.0\}$, $r \in \{0.1, 0.5, 0.9\}$, and approach (A–C). Var($\hat{\sigma}$) is sample variance of $\hat{\sigma}$ across 1,000 simulation replicates, reported for MLE, GLMM and MCMC.

I	σ	r	App.	**MLE**	**GLMM**	**MCMC**
20	0.1	0.1	A	8.73×10^{-6}	7.72×10^{-6}	7.86×10^{-6}
20	0.1	0.1	B	5.54×10^{-7}	8.00×10^{-8}	8.05×10^{-8}
20	0.1	0.1	C	7.90×10^{-7}	4.51×10^{-8}	4.51×10^{-8}
20	0.1	0.5	A	1.01×10^{-5}	7.73×10^{-6}	7.85×10^{-6}
20	0.1	0.5	B	2.37×10^{-6}	7.99×10^{-8}	8.01×10^{-8}
20	0.1	0.5	C	2.22×10^{-6}	4.51×10^{-8}	4.51×10^{-8}
20	0.1	0.9	A	3.28×10^{-5}	7.84×10^{-6}	7.83×10^{-6}
20	0.1	0.9	B	3.94×10^{-5}	8.00×10^{-8}	2.37×10^{-5}
20	0.1	0.9	C	3.97×10^{-5}	4.51×10^{-8}	4.51×10^{-8}
20	1.0	0.1	A	8.73×10^{-4}	7.72×10^{-4}	7.86×10^{-4}
20	1.0	0.1	B	5.54×10^{-5}	8.00×10^{-6}	8.05×10^{-6}
20	1.0	0.1	C	7.90×10^{-5}	4.51×10^{-6}	4.51×10^{-6}
20	1.0	0.5	A	1.01×10^{-3}	7.73×10^{-4}	7.85×10^{-4}
20	1.0	0.5	B	2.37×10^{-4}	7.99×10^{-6}	8.01×10^{-6}
20	1.0	0.5	C	2.22×10^{-4}	4.51×10^{-6}	4.51×10^{-6}
20	1.0	0.9	A	3.28×10^{-3}	7.84×10^{-4}	7.83×10^{-4}
20	1.0	0.9	B	3.94×10^{-3}	8.00×10^{-6}	2.37×10^{-3}
20	1.0	0.9	C	3.97×10^{-3}	4.51×10^{-6}	4.51×10^{-6}
50	0.1	0.1	A	3.91×10^{-6}	3.23×10^{-6}	3.25×10^{-6}
50	0.1	0.1	B	3.25×10^{-7}	3.02×10^{-8}	3.05×10^{-8}
50	0.1	0.1	C	4.01×10^{-7}	1.84×10^{-8}	1.84×10^{-8}
50	0.1	0.5	A	2.84×10^{-6}	3.28×10^{-6}	3.26×10^{-6}
50	0.1	0.5	B	3.05×10^{-6}	3.02×10^{-8}	3.05×10^{-8}
50	0.1	0.5	C	3.08×10^{-6}	1.84×10^{-8}	1.84×10^{-8}
50	0.1	0.9	A	7.84×10^{-5}	3.26×10^{-6}	3.26×10^{-6}
50	0.1	0.9	B	1.06×10^{-4}	3.02×10^{-8}	2.40×10^{-5}
50	0.1	0.9	C	1.05×10^{-4}	1.84×10^{-8}	1.84×10^{-8}
50	1.0	0.1	A	3.91×10^{-4}	3.23×10^{-4}	3.25×10^{-4}
50	1.0	0.1	B	3.25×10^{-5}	3.02×10^{-6}	3.05×10^{-6}
50	1.0	0.1	C	4.01×10^{-5}	1.84×10^{-6}	1.84×10^{-6}
50	1.0	0.5	A	2.84×10^{-4}	3.28×10^{-4}	3.26×10^{-4}
50	1.0	0.5	B	3.05×10^{-4}	3.02×10^{-6}	3.05×10^{-6}
50	1.0	0.5	C	3.08×10^{-4}	1.84×10^{-6}	1.84×10^{-6}
50	1.0	0.9	A	7.84×10^{-3}	3.26×10^{-4}	3.26×10^{-4}
50	1.0	0.9	B	1.06×10^{-2}	3.02×10^{-6}	2.40×10^{-3}
50	1.0	0.9	C	1.05×10^{-2}	1.84×10^{-6}	1.84×10^{-6}

TABLE S15 Empirical Mean squared error of the estimated $\hat{\sigma}$ under all 36 random-mean simulation scenarios for each combination of $I \in \{20, 50\}$, $\sigma \in \{0.1, 1.0\}$, $r \in \{0.1, 0.5, 0.9\}$, and approach (A–C). MSE computed as $\mathbb{E}\left[(\hat{\sigma} - \sigma_{\text{true}})^2\right]$ across 1,000 simulation replicates, reported for MLE, GLMM and MCMC.

I	σ	r	App.	**MLE**	**GLMM**	**MCMC**
20	0.1	0.1	A	4.72×10^{-5}	9.52×10^{-6}	1.05×10^{-5}
20	0.1	0.1	B	6.27×10^{-7}	8.05×10^{-8}	8.12×10^{-8}
20	0.1	0.1	C	9.17×10^{-7}	4.50×10^{-8}	4.51×10^{-8}
20	0.1	0.5	A	2.94×10^{-5}	9.67×10^{-6}	1.06×10^{-5}
20	0.1	0.5	B	9.98×10^{-6}	8.04×10^{-8}	8.08×10^{-8}
20	0.1	0.5	C	8.27×10^{-6}	4.50×10^{-8}	4.51×10^{-8}
20	0.1	0.9	A	2.94×10^{-4}	9.78×10^{-6}	1.06×10^{-5}
20	0.1	0.9	B	4.30×10^{-4}	8.05×10^{-8}	2.43×10^{-3}
20	0.1	0.9	C	4.25×10^{-4}	4.50×10^{-8}	4.51×10^{-8}
20	1.0	0.1	A	4.72×10^{-3}	9.52×10^{-4}	1.05×10^{-3}
20	1.0	0.1	B	6.27×10^{-5}	8.05×10^{-6}	8.12×10^{-6}
20	1.0	0.1	C	9.17×10^{-5}	4.50×10^{-6}	4.51×10^{-6}
20	1.0	0.5	A	2.94×10^{-3}	9.67×10^{-4}	1.06×10^{-3}
20	1.0	0.5	B	9.98×10^{-4}	8.04×10^{-6}	8.08×10^{-6}
20	1.0	0.5	C	8.27×10^{-4}	4.50×10^{-6}	4.51×10^{-6}
20	1.0	0.9	A	2.94×10^{-2}	9.78×10^{-4}	1.06×10^{-3}
20	1.0	0.9	B	4.30×10^{-2}	8.05×10^{-6}	2.43×10^{-2}
20	1.0	0.9	C	4.25×10^{-2}	4.50×10^{-6}	4.51×10^{-6}
50	0.1	0.1	A	3.79×10^{-5}	4.71×10^{-6}	5.08×10^{-6}
50	0.1	0.1	B	3.36×10^{-7}	7.95×10^{-8}	8.00×10^{-8}
50	0.1	0.1	C	5.92×10^{-7}	1.84×10^{-8}	1.84×10^{-8}
50	0.1	0.5	A	2.62×10^{-5}	4.87×10^{-6}	5.19×10^{-6}
50	0.1	0.5	B	1.35×10^{-5}	7.95×10^{-8}	8.01×10^{-8}
50	0.1	0.5	C	8.48×10^{-6}	1.84×10^{-8}	1.84×10^{-8}
50	0.1	0.9	A	1.11×10^{-3}	4.80×10^{-6}	5.16×10^{-6}
50	0.1	0.9	B	3.53×10^{-3}	7.95×10^{-8}	2.40×10^{-3}
50	0.1	0.9	C	3.47×10^{-3}	1.84×10^{-8}	1.84×10^{-8}
50	1.0	0.1	A	3.79×10^{-3}	4.71×10^{-4}	5.08×10^{-4}
50	1.0	0.1	B	3.36×10^{-5}	7.95×10^{-6}	8.00×10^{-6}
50	1.0	0.1	C	5.92×10^{-5}	1.84×10^{-6}	1.84×10^{-6}
50	1.0	0.5	A	2.62×10^{-3}	4.87×10^{-4}	5.19×10^{-4}
50	1.0	0.5	B	1.35×10^{-3}	7.95×10^{-6}	8.01×10^{-6}
50	1.0	0.5	C	8.48×10^{-4}	1.84×10^{-6}	1.84×10^{-6}
50	1.0	0.9	A	1.11×10^{-1}	4.80×10^{-4}	5.16×10^{-4}
50	1.0	0.9	B	3.53×10^{-1}	7.95×10^{-6}	2.40×10^{-2}
50	1.0	0.9	C	3.47×10^{-1}	1.84×10^{-6}	1.84×10^{-6}

TABLE S16 Empirical coverage probabilities of nominal 95% confidence intervals of the estimated $\hat{\sigma}$ under all 36 random-mean simulation scenarios for each combination of $I \in \{20, 50\}$, $\sigma \in \{0.1, 1.0\}$, $r \in \{0.1, 0.5, 0.9\}$, and approach (A–C). Coverage is defined as the proportion of 1,000 simulation replicates in which the interval contains the true value, reported for MLE, GLMM and MCMC.

I	σ	r	App.	**MLE**	**GLMM**	**MCMC**
20	0.1	0.1	A	0.44	0.92	0.91
20	0.1	0.1	B	0.98	0.95	0.95
20	0.1	0.1	C	0.98	0.94	0.94
20	0.1	0.5	A	0.70	0.92	0.91
20	0.1	0.5	B	0.61	0.95	0.95
20	0.1	0.5	C	0.63	0.94	0.94
20	0.1	0.9	A	0.35	0.92	0.91
20	0.1	0.9	B	0.22	0.95	0.99
20	0.1	0.9	C	0.18	0.94	0.94
20	1.0	0.1	A	0.44	0.92	0.91
20	1.0	0.1	B	0.98	0.95	0.95
20	1.0	0.1	C	0.98	0.94	0.94
20	1.0	0.5	A	0.70	0.92	0.91
20	1.0	0.5	B	0.61	0.95	0.95
20	1.0	0.5	C	0.63	0.94	0.94
20	1.0	0.9	A	0.35	0.92	0.91
20	1.0	0.9	B	0.22	0.95	0.99
20	1.0	0.9	C	0.18	0.94	0.94
50	0.1	0.1	A	0.34	0.92	0.92
50	0.1	0.1	B	0.99	0.95	0.95
50	0.1	0.1	C	0.99	0.95	0.95
50	0.1	0.5	A	0.77	0.92	0.92
50	0.1	0.5	B	0.55	0.95	0.95
50	0.1	0.5	C	0.65	0.95	0.95
50	0.1	0.9	A	0.19	0.92	0.92
50	0.1	0.9	B	0.05	0.95	0.99
50	0.1	0.9	C	0.04	0.95	0.95
50	1.0	0.1	A	0.34	0.92	0.92
50	1.0	0.1	B	0.99	0.95	0.95
50	1.0	0.1	C	0.99	0.95	0.95
50	1.0	0.5	A	0.77	0.92	0.92
50	1.0	0.5	B	0.55	0.95	0.95
50	1.0	0.5	C	0.65	0.95	0.95
50	1.0	0.9	A	0.19	0.92	0.92
50	1.0	0.9	B	0.05	0.95	0.99
50	1.0	0.9	C	0.04	0.95	0.95

TABLE S17 Empirical bias of the estimated $\hat{\tau}$ under all 36 random-mean simulation scenarios for each combination of $I \in \{20, 50\}$, $\sigma \in \{0.1, 1.0\}$, $r \in \{0.1, 0.5, 0.9\}$, and approach (A–C). Bias is computed as the average of $\hat{\tau} - \tau_{\text{true}}$ across 1,000 simulation replicates, reported for MLE, GLMM and MCMC.

I	σ	r	App.	**MLE**	**GLMM**	**MCMC**
20	0.1	0.1	A	1.590×10^{-3}	2.110×10^{-3}	5.410×10^{-3}
20	0.1	0.1	B	-5.670×10^{-5}	1.970×10^{-3}	1.650×10^{-2}
20	0.1	0.1	C	-1.270×10^{-3}	1.970×10^{-3}	5.230×10^{-3}
20	0.1	0.5	A	-5.250×10^{-3}	-3.510×10^{-3}	6.240×10^{-3}
20	0.1	0.5	B	-9.850×10^{-3}	-4.060×10^{-3}	5.630×10^{-3}
20	0.1	0.5	C	-1.310×10^{-2}	-4.090×10^{-3}	8.380×10^{-3}
20	0.1	0.9	A	-3.230×10^{-2}	-1.050×10^{-2}	1.860×10^{-2}
20	0.1	0.9	B	-4.170×10^{-2}	-1.220×10^{-2}	2.770×10^{-2}
20	0.1	0.9	C	-5.050×10^{-2}	-1.230×10^{-2}	1.690×10^{-2}
50	0.1	0.1	A	-8.740×10^{-4}	6.140×10^{-4}	3.020×10^{-3}
50	0.1	0.1	B	-8.950×10^{-4}	6.040×10^{-4}	3.010×10^{-3}
50	0.1	0.1	C	-2.040×10^{-3}	4.950×10^{-4}	3.050×10^{-3}
50	0.1	0.5	A	-4.950×10^{-3}	-1.830×10^{-3}	5.080×10^{-3}
50	0.1	0.5	B	-4.930×10^{-3}	-1.830×10^{-3}	5.070×10^{-3}
50	0.1	0.5	C	-9.920×10^{-3}	-1.950×10^{-3}	4.750×10^{-3}
50	0.1	0.9	A	-1.900×10^{-2}	-5.540×10^{-3}	1.070×10^{-2}
50	0.1	0.9	B	-1.900×10^{-2}	-5.520×10^{-3}	1.060×10^{-2}
50	0.1	0.9	C	-3.810×10^{-2}	-5.880×10^{-3}	1.130×10^{-2}
20	1.0	0.1	A	-1.430×10^{-2}	-8.870×10^{-3}	2.440×10^{-2}
20	1.0	0.1	B	-1.600×10^{-2}	-9.140×10^{-3}	2.420×10^{-2}
20	1.0	0.1	C	-2.000×10^{-2}	-9.240×10^{-3}	2.580×10^{-2}
20	1.0	0.5	A	-5.160×10^{-2}	-3.430×10^{-2}	6.310×10^{-2}
20	1.0	0.5	B	-8.550×10^{-2}	-3.980×10^{-2}	5.840×10^{-2}
20	1.0	0.5	C	-1.120×10^{-1}	-4.010×10^{-2}	6.510×10^{-2}
20	1.0	0.9	A	-2.510×10^{-1}	-1.020×10^{-1}	1.600×10^{-1}
20	1.0	0.9	B	-3.690×10^{-1}	-1.190×10^{-1}	1.370×10^{-1}
20	1.0	0.9	C	-4.750×10^{-1}	-1.200×10^{-1}	1.160×10^{-1}
50	1.0	0.1	A	-4.840×10^{-3}	-4.060×10^{-4}	1.040×10^{-2}
50	1.0	0.1	B	-5.060×10^{-3}	-3.820×10^{-4}	1.040×10^{-2}
50	1.0	0.1	C	-1.140×10^{-2}	-4.940×10^{-4}	1.060×10^{-2}
50	1.0	0.5	A	-2.380×10^{-2}	-1.850×10^{-2}	1.900×10^{-2}
50	1.0	0.5	B	-2.370×10^{-2}	-1.840×10^{-2}	1.910×10^{-2}
50	1.0	0.5	C	-4.850×10^{-2}	-1.920×10^{-2}	2.100×10^{-2}
50	1.0	0.9	A	-1.030×10^{-1}	-5.540×10^{-2}	4.240×10^{-2}
50	1.0	0.9	B	-1.030×10^{-1}	-5.520×10^{-2}	4.160×10^{-2}
50	1.0	0.9	C	-2.060×10^{-1}	-5.880×10^{-2}	4.700×10^{-2}

TABLE S18 Empirical variance of the estimated $\hat{\tau}$ under all 36 random-mean simulation scenarios for each combination of $I \in \{20, 50\}$, $\sigma \in \{0.1, 1.0\}$, $r \in \{0.1, 0.5, 0.9\}$, and approach (A–C). Var($\hat{\tau}$) is sample variance of $\hat{\tau}$ across 1,000 simulation replicates, reported for MLE, GLMM and MCMC.

I	σ	r	App.	**MLE**	**GLMM**	**MCMC**
20	0.1	0.1	A	5.000×10^{-5}	2.890×10^{-5}	3.610×10^{-5}
20	0.1	0.1	B	5.200×10^{-5}	2.810×10^{-5}	1.280×10^{-1}
20	0.1	0.1	C	6.100×10^{-5}	2.810×10^{-5}	3.390×10^{-5}
20	0.1	0.5	A	3.060×10^{-4}	2.410×10^{-4}	2.930×10^{-4}
20	0.1	0.5	B	4.810×10^{-4}	2.510×10^{-4}	3.130×10^{-4}
20	0.1	0.5	C	5.320×10^{-4}	2.500×10^{-4}	8.840×10^{-3}
20	0.1	0.9	A	4.110×10^{-3}	2.130×10^{-3}	2.600×10^{-3}
20	0.1	0.9	B	5.120×10^{-3}	2.260×10^{-3}	8.880×10^{-2}
20	0.1	0.9	C	5.780×10^{-3}	2.250×10^{-3}	2.960×10^{-3}
50	0.1	0.1	A	1.710×10^{-5}	1.150×10^{-5}	1.370×10^{-5}
50	0.1	0.1	B	1.660×10^{-5}	1.130×10^{-5}	1.380×10^{-5}
50	0.1	0.1	C	2.060×10^{-5}	1.090×10^{-5}	1.370×10^{-5}
50	0.1	0.5	A	1.140×10^{-4}	9.950×10^{-5}	1.160×10^{-4}
50	0.1	0.5	B	1.140×10^{-4}	9.880×10^{-5}	1.160×10^{-4}
50	0.1	0.5	C	2.030×10^{-4}	9.780×10^{-5}	1.190×10^{-4}
50	0.1	0.9	A	9.970×10^{-4}	8.900×10^{-4}	1.030×10^{-3}
50	0.1	0.9	B	9.980×10^{-4}	8.880×10^{-4}	1.030×10^{-3}
50	0.1	0.9	C	1.900×10^{-3}	8.790×10^{-4}	1.160×10^{-3}
20	1.0	0.1	A	4.970×10^{-3}	2.950×10^{-3}	3.590×10^{-3}
20	1.0	0.1	B	5.280×10^{-3}	2.870×10^{-3}	3.630×10^{-3}
20	1.0	0.1	C	6.240×10^{-3}	2.870×10^{-3}	3.710×10^{-3}
20	1.0	0.5	A	3.110×10^{-2}	2.490×10^{-2}	2.960×10^{-2}
20	1.0	0.5	B	5.190×10^{-2}	2.590×10^{-2}	3.220×10^{-2}
20	1.0	0.5	C	6.020×10^{-2}	2.580×10^{-2}	3.040×10^{-2}
20	1.0	0.9	A	2.570×10^{-1}	2.210×10^{-1}	5.950×10^{-1}
20	1.0	0.9	B	4.100×10^{-1}	2.340×10^{-1}	6.920×10^{-1}
20	1.0	0.9	C	4.970×10^{-1}	2.330×10^{-1}	5.890×10^{-1}
50	1.0	0.1	A	1.520×10^{-3}	1.130×10^{-3}	1.320×10^{-3}
50	1.0	0.1	B	1.470×10^{-3}	1.110×10^{-3}	1.320×10^{-3}
50	1.0	0.1	C	1.950×10^{-3}	1.090×10^{-3}	1.320×10^{-3}
50	1.0	0.5	A	1.130×10^{-2}	9.950×10^{-3}	1.150×10^{-2}
50	1.0	0.5	B	1.130×10^{-2}	9.880×10^{-3}	1.150×10^{-2}
50	1.0	0.5	C	2.050×10^{-2}	9.780×10^{-3}	1.200×10^{-2}
50	1.0	0.9	A	1.030×10^{-1}	8.900×10^{-2}	1.020×10^{-1}
50	1.0	0.9	B	1.030×10^{-1}	8.880×10^{-2}	1.020×10^{-1}
50	1.0	0.9	C	2.000×10^{-1}	8.790×10^{-2}	1.050×10^{-1}

TABLE S19 Empirical Mean squared error of the estimated $\hat{\tau}$ under all 36 random-mean simulation scenarios for each combination of $I \in \{20, 50\}$, $\sigma \in \{0.1, 1.0\}$, $r \in \{0.1, 0.5, 0.9\}$, and approach (A–C). MSE computed as $\mathbb{E}\left[(\hat{\tau} - \tau_{\text{true}})^2\right]$ across 1,000 simulation replicates, reported for MLE, GLMM and MCMC.

I	σ	r	App.	**MLE**	**GLMM**	**MCMC**
20	0.1	0.1	A	5.250×10^{-5}	3.340×10^{-5}	6.530×10^{-5}
20	0.1	0.1	B	5.200×10^{-5}	3.200×10^{-5}	1.280×10^{-1}
20	0.1	0.1	C	6.260×10^{-5}	3.200×10^{-5}	6.120×10^{-5}
20	0.1	0.5	A	3.330×10^{-4}	2.530×10^{-4}	3.320×10^{-4}
20	0.1	0.5	B	5.780×10^{-4}	2.680×10^{-4}	3.440×10^{-4}
20	0.1	0.5	C	7.040×10^{-4}	2.660×10^{-4}	8.900×10^{-3}
20	0.1	0.9	A	5.150×10^{-3}	2.240×10^{-3}	2.940×10^{-3}
20	0.1	0.9	B	6.860×10^{-3}	2.410×10^{-3}	8.950×10^{-2}
20	0.1	0.9	C	8.330×10^{-3}	2.400×10^{-3}	3.240×10^{-3}
50	0.1	0.1	A	1.790×10^{-5}	1.190×10^{-5}	2.280×10^{-5}
50	0.1	0.1	B	1.740×10^{-5}	1.170×10^{-5}	2.290×10^{-5}
50	0.1	0.1	C	2.470×10^{-5}	1.110×10^{-5}	2.300×10^{-5}
50	0.1	0.5	A	1.390×10^{-4}	1.030×10^{-4}	1.420×10^{-4}
50	0.1	0.5	B	1.380×10^{-4}	1.020×10^{-4}	1.420×10^{-4}
50	0.1	0.5	C	3.020×10^{-4}	1.010×10^{-4}	1.420×10^{-4}
50	0.1	0.9	A	1.360×10^{-3}	9.210×10^{-4}	1.140×10^{-3}
50	0.1	0.9	B	1.360×10^{-3}	9.190×10^{-4}	1.140×10^{-3}
50	0.1	0.9	C	3.360×10^{-3}	9.140×10^{-4}	1.290×10^{-3}
20	1.0	0.1	A	5.170×10^{-3}	3.030×10^{-3}	4.180×10^{-3}
20	1.0	0.1	B	5.530×10^{-3}	2.950×10^{-3}	4.210×10^{-3}
20	1.0	0.1	C	6.640×10^{-3}	2.960×10^{-3}	4.370×10^{-3}
20	1.0	0.5	A	3.380×10^{-2}	2.610×10^{-2}	3.360×10^{-2}
20	1.0	0.5	B	5.920×10^{-2}	2.750×10^{-2}	3.560×10^{-2}
20	1.0	0.5	C	7.280×10^{-2}	2.740×10^{-2}	3.460×10^{-2}
20	1.0	0.9	A	3.190×10^{-1}	2.310×10^{-1}	6.210×10^{-1}
20	1.0	0.9	B	5.470×10^{-1}	2.480×10^{-1}	7.110×10^{-1}
20	1.0	0.9	C	7.220×10^{-1}	2.470×10^{-1}	6.020×10^{-1}
50	1.0	0.1	A	1.540×10^{-3}	1.130×10^{-3}	1.430×10^{-3}
50	1.0	0.1	B	1.500×10^{-3}	1.110×10^{-3}	1.430×10^{-3}
50	1.0	0.1	C	2.080×10^{-3}	1.090×10^{-3}	1.440×10^{-3}
50	1.0	0.5	A	1.190×10^{-2}	1.030×10^{-2}	1.190×10^{-2}
50	1.0	0.5	B	1.190×10^{-2}	1.020×10^{-2}	1.190×10^{-2}
50	1.0	0.5	C	2.290×10^{-2}	1.010×10^{-2}	1.230×10^{-2}
50	1.0	0.9	A	1.140×10^{-1}	9.210×10^{-2}	1.040×10^{-1}
50	1.0	0.9	B	1.140×10^{-1}	9.190×10^{-2}	1.040×10^{-1}
50	1.0	0.9	C	2.420×10^{-1}	9.140×10^{-2}	1.080×10^{-1}

TABLE S20 Empirical coverage probabilities of nominal 95% confidence intervals of the estimated $\hat{\tau}$ under all 36 random-mean simulation scenarios for each combination of $I \in \{20, 50\}$, $\sigma \in \{0.1, 1.0\}$, $r \in \{0.1, 0.5, 0.9\}$, and approach (A–C). Coverage is defined as the proportion of 1,000 simulation replicates in which the interval contains the true value, reported for MLE, GLMM and MCMC.

I	σ	r	App.	**MLE**	**GLMM**	**MCMC**
20	0.1	0.1	A	0.94	0.93	0.87
20	0.1	0.1	B	0.94	0.94	1.00
20	0.1	0.1	C	0.93	0.92	0.85
20	0.1	0.5	A	0.95	0.94	0.93
20	0.1	0.5	B	0.93	0.94	0.94
20	0.1	0.5	C	0.91	0.95	1.00
20	0.1	0.9	A	0.92	0.95	0.93
20	0.1	0.9	B	0.90	0.94	1.00
20	0.1	0.9	C	0.87	0.95	0.93
50	0.1	0.1	A	0.95	0.95	0.88
50	0.1	0.1	B	0.94	0.95	0.88
50	0.1	0.1	C	0.94	0.95	0.88
50	0.1	0.5	A	0.94	0.95	0.91
50	0.1	0.5	B	0.94	0.95	0.91
50	0.1	0.5	C	0.93	0.95	0.90
50	0.1	0.9	A	0.92	0.95	0.90
50	0.1	0.9	B	0.92	0.95	0.90
50	0.1	0.9	C	0.91	0.95	0.91
20	1.0	0.1	A	0.95	0.95	0.93
20	1.0	0.1	B	0.94	0.94	0.93
20	1.0	0.1	C	0.94	0.94	0.93
20	1.0	0.5	A	0.95	0.95	0.93
20	1.0	0.5	B	0.94	0.95	0.93
20	1.0	0.5	C	0.94	0.95	0.93
20	1.0	0.9	A	0.95	0.95	0.99
20	1.0	0.9	B	0.94	0.95	0.99
20	1.0	0.9	C	0.94	0.95	0.99
50	1.0	0.1	A	0.95	0.95	0.93
50	1.0	0.1	B	0.95	0.95	0.93
50	1.0	0.1	C	0.94	0.95	0.93
50	1.0	0.5	A	0.95	0.95	0.94
50	1.0	0.5	B	0.95	0.95	0.94
50	1.0	0.5	C	0.94	0.95	0.94
50	1.0	0.9	A	0.94	0.95	0.94
50	1.0	0.9	B	0.94	0.95	0.94
50	1.0	0.9	C	0.94	0.95	0.94

TABLE S21 Computational burden, elapsed fitting time, and numerical diagnostics for the four methods across the 36 primary random-mean simulation scenarios. For each method and scenario, the median elapsed fitting time over simulation replicates was first calculated; the table reports the median and range of these 36 scenario-specific medians. All diagnostic entries are percentages unless otherwise stated.

Method	Main computational step	Median time (s) and (range)	Fitting and convergence diagnostics
GLMM	Laplace-type mixed-model optimization with a random intercept	0.057 (0.049–0.077)	Optimizer nonconvergence: 6.7%; singular fits: 0.0%.
GH-MLE	Fixed $G = 20$ Gauss–Hermite evaluations within outer likelihood optimization	2.144 (0.838–7.933)	Optimizer nonconvergence: 6.1%; invalid or penalty likelihood: 0.0%.
MCAI-MLE ($K = 10$)	Study-specific mode search, curvature calculation, and adaptive numerical integration within outer optimization	42.516 (11.488–173.153)	Optimizer nonconvergence: 5.6%; invalid or penalty likelihood: 9.7%.
Bayesian MCMC	Repeated Hamiltonian Monte Carlo likelihood-gradient evaluations across chains and iterations	67.742 (43.779–135.922)	Runtime error: 0.02%. Median maximum $\widehat{R}$: 1.054 (95th percentile: 2.203); median minimum ESS: 40.8 (5th percentile: 1.55). $\widehat{R} > 1.01$: 91.6%; ESS < 100: 75.4%; divergent transitions: 0.09%; maximum-treedepth warnings: 65.5%.

TABLE S22 Limited-data stress-test performance for all four methods. Entries are empirical bias (RMSE) across 500 replicates per scenario. Each scenario used $n_i \sim \text{Uniform}(10, 50)$ and 5–10 thresholds per study.

Panel A: μ_0; entries are Bias (RMSE)

I	σ	r	τ	GH-MLE	MCAI-MLE	GLMM	Bayesian MCMC
5	0.1	0.1	0.033	6.16×10^{-4} (0.016)	3.81×10^{-4} (0.016)	-5.27×10^{-5} (0.015)	-0.0021 (0.300)
5	0.1	0.5	0.100	3.80×10^{-4} (0.059)	-0.0019 (0.069)	-2.70×10^{-4} (0.049)	-0.020 (0.532)
5	0.1	0.9	0.300	-0.0043 (0.161)	-0.0018 (0.134)	-0.0011 (0.132)	-0.030 (0.925)
5	1.0	0.1	0.333	0.0052 (0.174)	0.0053 (0.173)	0.0029 (0.166)	0.0012 (0.173)
5	1.0	0.5	1.000	-0.0033 (0.531)	0.022 (0.442)	0.017 (0.444)	0.016 (0.453)
5	1.0	0.9	3.000	-0.134 (1.413)	-0.134 (1.326)	-0.139 (1.326)	-0.152 (1.284)
10	0.1	0.1	0.033	-2.90×10^{-4} (0.011)	-1.96×10^{-4} (0.011)	-2.71×10^{-4} (0.011)	-2.08×10^{-4} (0.011)
10	0.1	0.5	0.100	-0.0016 (0.038)	2.71×10^{-4} (0.033)	-5.37×10^{-5} (0.033)	6.06×10^{-5} (0.033)
10	0.1	0.9	0.300	0.0020 (0.114)	-3.99×10^{-4} (0.118)	-2.16×10^{-5} (0.095)	0.0083 (0.278)
10	1.0	0.1	0.333	-0.0058 (0.119)	-0.0066 (0.118)	-0.0056 (0.114)	-0.0060 (0.115)
10	1.0	0.5	1.000	0.022 (0.377)	0.016 (0.323)	0.014 (0.322)	0.014 (0.322)
10	1.0	0.9	3.000	-0.022 (1.093)	0.177 (4.593)	-0.019 (0.936)	-0.028 (0.927)

Panel B: σ; entries are Bias (RMSE)

I	σ	r	τ	GH-MLE	MCAI-MLE	GLMM	Bayesian MCMC
5	0.1	0.1	0.033	-0.011 (0.014)	-0.011 (0.014)	0.0043 (0.012)	5.564 (37.43)
5	0.1	0.5	0.100	-0.012 (0.015)	-0.013 (0.018)	0.0046 (0.012)	17.67 (167.8)
5	0.1	0.9	0.300	-0.0034 (0.016)	-0.018 (0.030)	0.0045 (0.012)	51.43 (263.0)
5	1.0	0.1	0.333	-0.106 (0.137)	-0.106 (0.137)	0.051 (0.122)	0.088 (0.158)
5	1.0	0.5	1.000	-0.127 (0.150)	-0.121 (0.157)	0.037 (0.108)	0.071 (0.135)
5	1.0	0.9	3.000	-0.020 (0.168)	-0.159 (0.260)	0.057 (0.126)	0.107 (0.334)
10	0.1	0.1	0.033	-0.011 (0.012)	-0.011 (0.012)	0.0038 (0.0078)	0.0054 (0.0091)
10	0.1	0.5	0.100	-0.012 (0.013)	-0.011 (0.013)	0.0044 (0.0089)	0.0060 (0.010)
10	0.1	0.9	0.300	0.0025 (0.013)	-0.017 (0.026)	0.0043 (0.0085)	6.129 (116.1)
10	1.0	0.1	0.333	-0.117 (0.130)	-0.117 (0.130)	0.032 (0.081)	0.048 (0.092)
10	1.0	0.5	1.000	-0.115 (0.129)	-0.114 (0.128)	0.039 (0.083)	0.054 (0.094)
10	1.0	0.9	3.000	0.043 (0.146)	-0.165 (0.697)	0.041 (0.086)	0.057 (0.098)

Panel C: τ; entries are Bias (RMSE)

I	σ	r	τ	GH-MLE	MCAI-MLE	GLMM	Bayesian MCMC
5	0.1	0.1	0.033	-0.0097 (0.019)	-0.010 (0.018)	-0.0058 (0.014)	0.122 (0.577)
5	0.1	0.5	0.100	-0.0058 (0.041)	-0.017 (0.037)	-0.015 (0.035)	0.176 (0.716)
5	0.1	0.9	0.300	-0.047 (0.118)	-0.051 (0.138)	-0.058 (0.106)	0.549 (1.382)
5	1.0	0.1	0.333	-0.116 (0.188)	-0.121 (0.182)	-0.068 (0.133)	0.139 (0.234)
5	1.0	0.5	1.000	-0.074 (0.365)	-0.171 (0.360)	-0.159 (0.344)	0.342 (0.565)
5	1.0	0.9	3.000	-0.281 (1.095)	-0.497 (1.046)	-0.515 (1.032)	0.510 (1.260)
10	0.1	0.1	0.033	-0.0078 (0.013)	-0.0079 (0.013)	-0.0033 (0.0090)	0.0043 (0.011)
10	0.1	0.5	0.100	-0.0040 (0.025)	-0.0091 (0.024)	-0.0080 (0.023)	0.014 (0.030)
10	0.1	0.9	0.300	-0.038 (0.088)	-0.025 (0.079)	-0.027 (0.068)	0.068 (0.315)
10	1.0	0.1	0.333	-0.071 (0.129)	-0.074 (0.126)	-0.033 (0.089)	0.042 (0.110)
10	1.0	0.5	1.000	-0.041 (0.256)	-0.101 (0.254)	-0.086 (0.244)	0.120 (0.303)
10	1.0	0.9	3.000	-0.223 (0.786)	-0.043 (4.525)	-0.264 (0.693)	0.215 (0.741)

TABLE S23 RMSE ratios under mild departures from normality relative to the matched normal reference scenario. Values larger than one indicate higher RMSE under the corresponding non-normal distribution. Each scenario used I = 20, study-size design A, and 500 replicates.

Panel A: μ_0

		t_8/Normal RMSE ratio				Mixture/Normal RMSE ratio			
σ	r	GH	MCAI	GLMM	MCMC	GH	MCAI	GLMM	MCMC
0.1	0.1	1.052	1.039	1.039	1.044	1.052	1.089	1.086	1.084
0.1	0.5	1.029	0.993	0.999	0.995	1.039	0.958	0.957	0.956
0.1	0.9	1.002	3.286	1.012	1.018	0.996	1.726	1.030	1.028
1.0	0.1	1.076	1.078	1.096	1.098	1.004	1.063	1.073	1.072
1.0	0.5	0.891	0.906	0.911	0.914	0.890	0.928	0.929	0.928
1.0	0.9	0.991	0.925	0.922	0.922	0.871	0.935	0.923	0.921

Panel B: σ

		t_8/Normal RMSE ratio				Mixture/Normal RMSE ratio			
σ	r	GH	MCAI	GLMM	MCMC	GH	MCAI	GLMM	MCMC
0.1	0.1	1.951	1.963	2.680	2.489	0.986	0.987	0.949	0.947
0.1	0.5	2.099	1.953	2.658	2.484	0.988	0.995	0.944	0.948
0.1	0.9	0.800	52.58	2.447	2.288	1.101	1.036	0.885	0.888
1.0	0.1	1.902	1.914	2.697	2.533	0.981	0.982	1.020	1.024
1.0	0.5	2.157	1.971	2.621	2.448	1.079	1.057	1.028	1.025
1.0	0.9	0.877	1.076	2.813	2.640	1.095	1.013	1.062	1.065

Panel C: τ

		t_8/Normal RMSE ratio				Mixture/Normal RMSE ratio			
σ	r	GH	MCAI	GLMM	MCMC	GH	MCAI	GLMM	MCMC
0.1	0.1	0.992	1.003	1.037	1.033	1.014	1.049	1.025	0.952
0.1	0.5	1.053	1.025	1.039	1.073	1.068	1.087	1.100	1.085
0.1	0.9	1.010	1.397	0.974	0.944	0.935	2.876	1.018	1.085
1.0	0.1	0.941	0.961	0.987	0.959	1.005	1.022	1.034	1.015
1.0	0.5	1.185	1.151	1.142	1.028	1.065	1.056	1.056	0.996
1.0	0.9	0.953	0.988	0.991	1.013	0.904	0.958	0.957	0.997
